# Turning *Escherichia coli* into a Frataxin-Dependent Organism

**DOI:** 10.1371/journal.pgen.1005134

**Published:** 2015-05-21

**Authors:** Béatrice Roche, Rym Agrebi, Allison Huguenot, Sandrine Ollagnier de Choudens, Frédéric Barras, Béatrice Py

**Affiliations:** 1 Laboratoire de Chimie Bactérienne, UMR 7283, Aix-Marseille Université-CNRS, Institut de Microbiologie de la Méditerranée, Marseille, France; 2 De Duve Institute, Université Catholique de Louvain, Brussels, Belgium; 3 DSV/iRTSV/CBM, UMR 5249 CEA-Université de Grenoble I-CNRS/Equipe Biocatalyse, CEA-Grenoble, Grenoble, France; Universidad de Sevilla, Spain

## Abstract

Fe-S bound proteins are ubiquitous and contribute to most basic cellular processes. A defect in the ISC components catalyzing Fe-S cluster biogenesis leads to drastic phenotypes in both eukaryotes and prokaryotes. In this context, the Frataxin protein (FXN) stands out as an exception. In eukaryotes, a defect in FXN results in severe defects in Fe-S cluster biogenesis, and in humans, this is associated with Friedreich’s ataxia, a neurodegenerative disease. In contrast, prokaryotes deficient in the FXN homolog CyaY are fully viable, despite the clear involvement of CyaY in ISC-catalyzed Fe-S cluster formation. The molecular basis of the differing importance in the contribution of FXN remains enigmatic. Here, we have demonstrated that a single mutation in the scaffold protein IscU rendered *E*. *coli* viability strictly dependent upon a functional CyaY. Remarkably, this mutation changed an Ile residue, conserved in prokaryotes at position 108, into a Met residue, conserved in eukaryotes. We found that in the double mutant *IscU_IM_ ΔcyaY*, the ISC pathway was completely abolished, becoming equivalent to the *ΔiscU* deletion strain and recapitulating the drastic phenotype caused by FXN deletion in eukaryotes. Biochemical analyses of the “eukaryotic-like” IscU_IM_ scaffold revealed that it exhibited a reduced capacity to form Fe-S clusters. Finally, bioinformatic studies of prokaryotic IscU proteins allowed us to trace back the source of FXN-dependency as it occurs in present-day eukaryotes. We propose an evolutionary scenario in which the current mitochondrial Isu proteins originated from the IscU_IM_ version present in the ancestor of the *Rickettsiae*. Subsequent acquisition of SUF, the second Fe-S cluster biogenesis system, in bacteria, was accompanied by diminished contribution of CyaY in prokaryotic Fe-S cluster biogenesis, and increased tolerance to change in the amino acid present at the 108^th^ position of the scaffold.

## Author Summary

Iron sulfur (Fe-S) clusters are ubiquitous cofactors found in proteins which function in very diverse pathways ranging from respiration to DNA repair. The mitochondrial Fe-S biogenesis machinery ISC was inherited from the bacterial ancestor of mitochondria. In both prokaryotes and eukaryotes, deficiency of core ISC components is associated with drastic decrease in Fe-S proteins activities and causes severe phenotypes. In this context, the case of frataxin, an ISC associated component, is surprising since the lack of frataxin in prokaryotes leads to very mild phenotypes in comparison to eukaryotes. Here, we showed that in an *E*. *coli* strain, a single mutation in a key component of the Fe-S cluster biogenesis pathway, namely the scaffold protein, was sufficient to impose a strict frataxin dependency. Remarkably, this mutation substituted an Ile residue that is conserved in prokaryotic scaffolds, for one Met residue that is conserved in eukaryotic scaffolds. These results provide a lead towards understanding the differences between otherwise highly related prokaryotic and eukaryotic ISC Fe-S cluster biogenesis machineries, and provide a new entry point into deciphering the molecular role of frataxin.

## Introduction

Fe-S bound proteins are ubiquitous and involved in a wide variety of cellular processes such as respiration, regulation of gene expression and central metabolism [1,2]. Maturation of Fe-S proteins is an essential cellular process for both eukaryotic and prokaryotic organisms. The mitochondrial ISC Fe-S biogenesis machinery has been proposed to be inherited from a bacterial ancestor, and they function in a similar way by utilizing two major steps: (i) an assembly step in which the cluster forms transiently on a scaffold protein, and (ii) a delivery step in which the cluster is transferred to apotargets *via* dedicated carriers [3–5]. The ISC scaffold (Isu for eukaryotes / IscU for prokaryotes) contains three conserved cysteine residues that are essential for Fe-S cluster binding and a conserved motif that is specifically recognized by DnaKJ related chaperones/co-chaperones to facilitate cluster release [6–10]. Sulfur is produced from L-cysteine by the cysteine desulfurase, (Nfs1 for eukaryotes / IscS for prokaryotes) a pyridoxal-5’-phosphate (PLP)-dependent enzyme [11–15]. The sulfur is bound in the form of a persulfide to an active-site cysteine residue of the cysteine desulfurase and is subsequently transferred to the scaffold [15–18]. Frataxin (FXN in human, Yfh1 in yeast and CyaY in bacteria) is a protein present in mammals, plants and bacteria [19]. FXN interacts with the cysteine desulfurase/scaffold complex [20–26]. In both prokaryotes and eukaryotes, deficiency of core ISC components including the ISC scaffold or cysteine desulfurase is associated with severely defective Fe-S cluster biogenesis that translates into drastic phenotypes [12–14,26–29]. In contrast, the consequences resulting from deficiency in FXN differ in eukaryotes or prokaryotes. In yeast, deficiency in frataxin (Yfh1) results in defective growth, mitochondrial iron accumulation, decreased heme synthesis, loss of Fe-S cluster protein activity and hypersensitivity to oxidants [30–34]. In humans, altered levels of FXN lead to a drastic decrease in Fe-S protein activities and cause the neurodegenerative disease Friedreich’s ataxia [35–39]. We and others recently established the participation of *E*. *coli* frataxin (CyaY) in ISC-assisted biogenesis of Fe-S clusters. Accordingly, Δ*cyaY* mutants exhibit pleiotropic but mild phenotypes [40–44]. Both the physiological advantage and the molecular reasons underlying this apparent loss in importance of CyaY in prokaryotes remain obscure.

Recently, in their analysis in *Saccharomyces cerevisiae*, the Dancis lab reported that a point mutation in the scaffold protein Isu1 could bypass a Yfh1 deletion [45]. This demonstrated that a single mutation could make survival of a eukaryote independent of FXN. In the present study, we investigated whether the reverse was true, *i*.*e*. could *E*. *coli* be turned into a CyaY(FXN)-dependent organism. This proved to be possible and required a single amino acid change in the IscU scaffold as well. Genetic, physiological, biochemical, bioinformatic and phylogenomic approaches were carried out to characterize this *E*. *coli* variant. The results of these studies led us to propose an evolutionary scenario according to which frataxin is an ISC-associated factor that appeared in Proteobacteria. It was then acquired by eukaryotes via endosymbiotic mitochondrial event where it became essential. Meanwhile its importance diminished in bacteria possibly because these later contained other Fe-S cluster biogenesis systems, such as SUF in many instances.

## Results

### CyaY is essential in a “eukaryotized” *E*. *coli*

**Fig 1:**
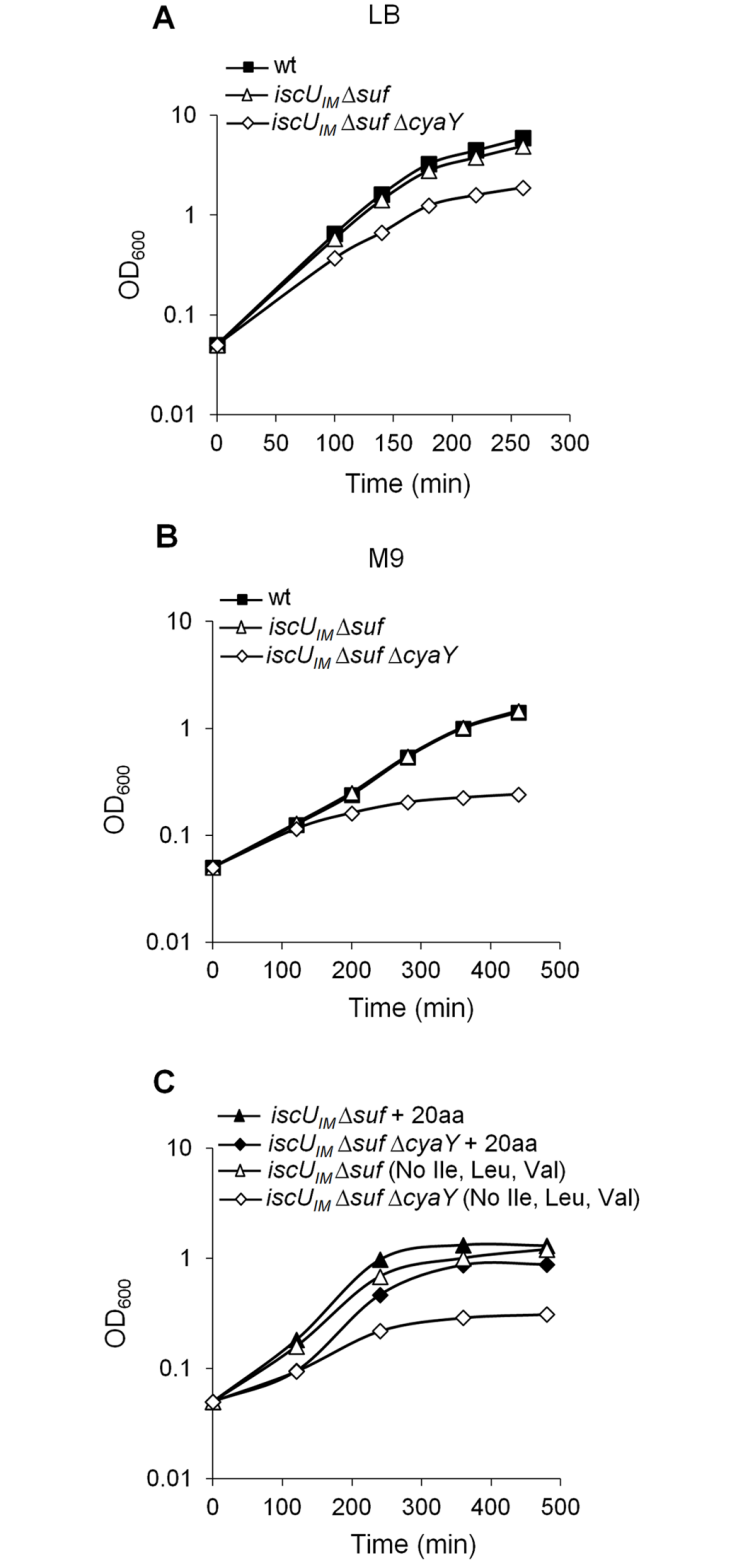
The *iscU*_*IM*_ Δ*suf* Δ*cyaY* strain exhibits growth defect. Growth of wt (DV901), *iscU*_*IM*_ **Δ** *suf* (BR763) and *iscU*_*IM*_ **Δ** *suf* *Δ* *cyaY* (BR767) strains in LB (A). The wt (DV901), *iscU*_*IM*_ **Δ** *suf* (BR763) and *iscU*_*IM*_ *Δ* *suf* *ΔcyaY* (BR767) strains were grown overnight in glucose M9 minimal medium supplemented with all 20 amino acids. Cultures were then diluted into fresh glucose M9 minimal medium (B). Strains *iscU*_*IM*_ **Δ** *suf* (BR763) and *iscU*_*IM*_ **Δ** *suf* *ΔcyaY* (BR767) were grown overnight in glucose M9 minimal medium supplemented with all 20 amino acids. Cultures were then diluted into a fresh glucose M9 minimal medium supplemented with all amino acids or with all except Ile, Leu and Val (C). Growth was monitored at 600 nm. The experiment was repeated at least three times. One representative experiment is shown.

The studies by the Dancis group revealed that the contribution of frataxin to Fe-S cluster biogenesis might depend on the identity of the residue present at position 108 in IscU [45]. To test this hypothesis, we exchanged the 108^th^ ATT Ile codon with an ATG Met codon in the *iscU* sequence, and the cognate *iscU*_*I108M*_ allele was introduced into the *E*. *coli* chromosome, giving rise to the BR755 (*iscU*_*IM*_) strain. Moreover, in order to make this strain more eukaryotic-like, we deleted the *suf* operon encoding the second *E*. *coli* Fe-S cluster biogenesis system, giving rise to the BR763 (*iscU*_*IM*_ Δ*suf*) strain. Growth of the BR763 strain in LB or in minimal M9 medium was similar to the reference strain DV901 (Fig 1A and 1B). In contrast, introduction of the *cyaY* deletion in the *iscU*_*IM*_ Δ*suf* strain had a drastic negative impact on growth in glucose M9 minimal medium (Fig 1B). To test whether the growth defect of the *iscU*_*IM*_ Δ*suf* Δ*cyaY* strain in minimal medium was related to defects in Fe-S proteins, we tested whether it was auxotrophic for the amino acids Ile, Leu, and Val whose synthesis depends on Fe-S enzymes. Addition of all of the 20 amino acids restored growth, whereas omitting Ile, Leu, and Val failed to rescue growth (Fig 1C). However, adding only Ile, Leu, and Val failed to restore growth showing that Ile, Leu, and Val were necessary but not sufficient. Adding Cys and Met in addition to Ile, Leu, and Val, did not rescue growth of the *iscU*_*IM*_ Δ*suf* Δ*cyaY* indicating that other processes must also be impaired in this strain. Addition of vitamins improved marginally growth of the *iscU*_*IM*_ Δ*suf* Δ*cyaY* strain (S1 Fig). In rich medium, the growth defect of the *iscU*_*IM*_ Δ*suf* Δ*cyaY* strain indicated that, in addition to nutritional requirements, this strain was also impaired in other processes (Fig 1A).

**Fig 2:**
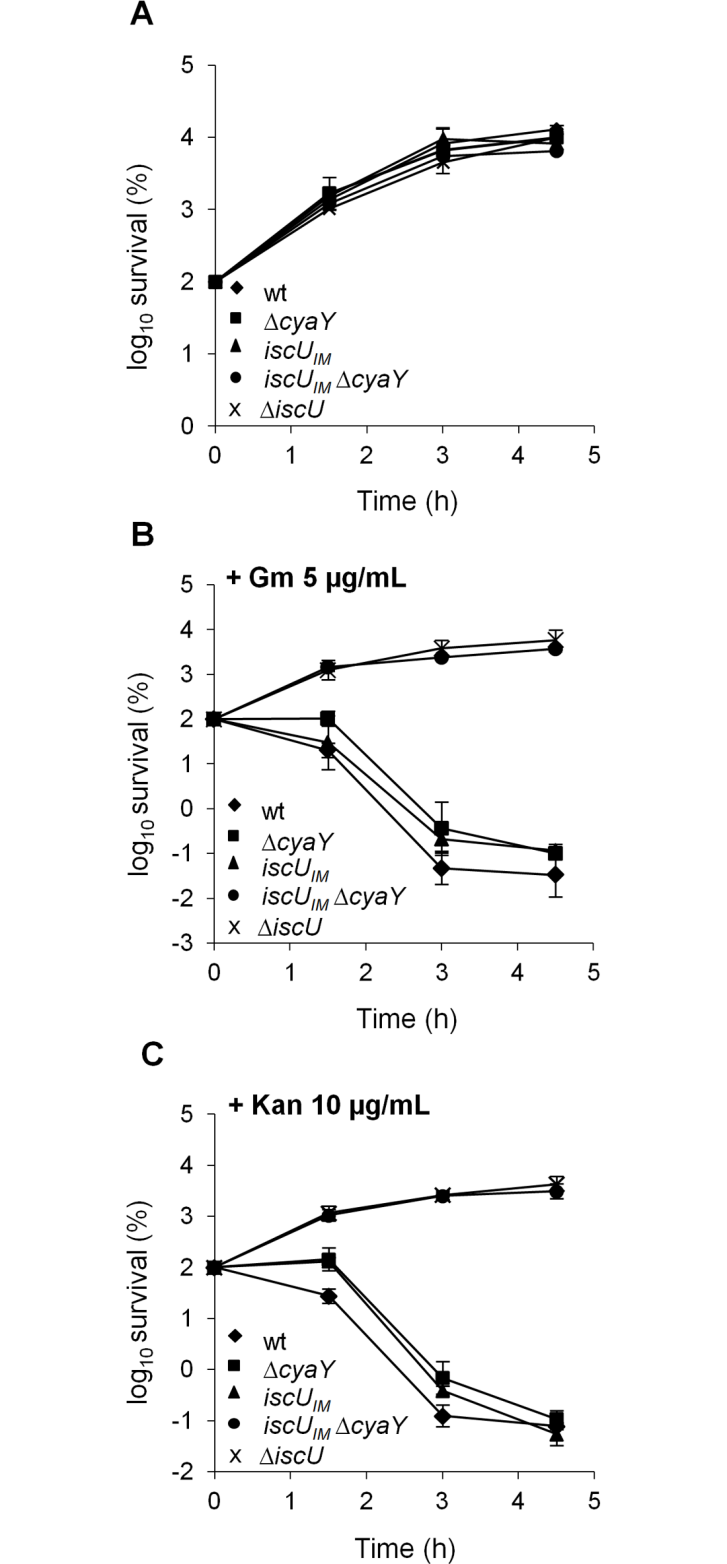
The *iscU*_*IM*_ Δ*cyaY* strain is resistant to aminoglycosides. Survival of wt (DV901), *iscU*_*IM*_ (BR755) and their Δ*cyaY* derivatives (DV925 and BR756) without antibiotic (A) and after (B) Gentamicin (Gm) (5 μg/mL) and Kanamycin (Kan) (10 μg/mL) (C) treatment. Survival, measured by colony-forming units (CFU) per mL, was normalized relative to time zero at which the antibiotic was added (midexponential phase cells; ~5 ×10^7^ CFU/mL) and was plotted as log_*10*_ of % survival. Error bars represent the standard error from three independent experiments.

A second assay measuring killing efficiency by aminoglycosides (gentamicin, Gm, and kanamycin, Kan) was used. This assay is an indirect read-out of ISC-mediated Fe-S cluster biogenesis efficiency but is independent of SUF functioning. Indeed, uptake of aminoglycosides is dependent upon proton motive force (p.m.f) at the cytoplasmic membrane, which depends upon the activity of Nuo (also called Complex I), a multi-protein complex containing 9 Fe-S clusters, whose maturation depends predominantly on the ISC system and only marginally on the SUF system [46]. The *iscU*_*IM*_ strain was found to exhibit wild-type sensitivity to Gm and Kan, whereas the Δ*cyaY* derivative *iscU*_*IM*_ Δ*cyaY* showed enhanced resistance, again suggesting that ISC dependent Fe-S cluster biogenesis was compromised in the absence of CyaY in this background (Fig 2). As a matter of fact, the *iscU*_*IM*_ Δ*cyaY* strain exhibited a level of resistance similar to that of a Δ*iscU* strain, illustrating the important contribution of CyaY in a background using a eukaryotic-like IscU_IM_ scaffold. In contrast, the wt strain remained sensitive to Gm and Kan whether or not CyaY was present (Fig 2).

**Fig 3:**
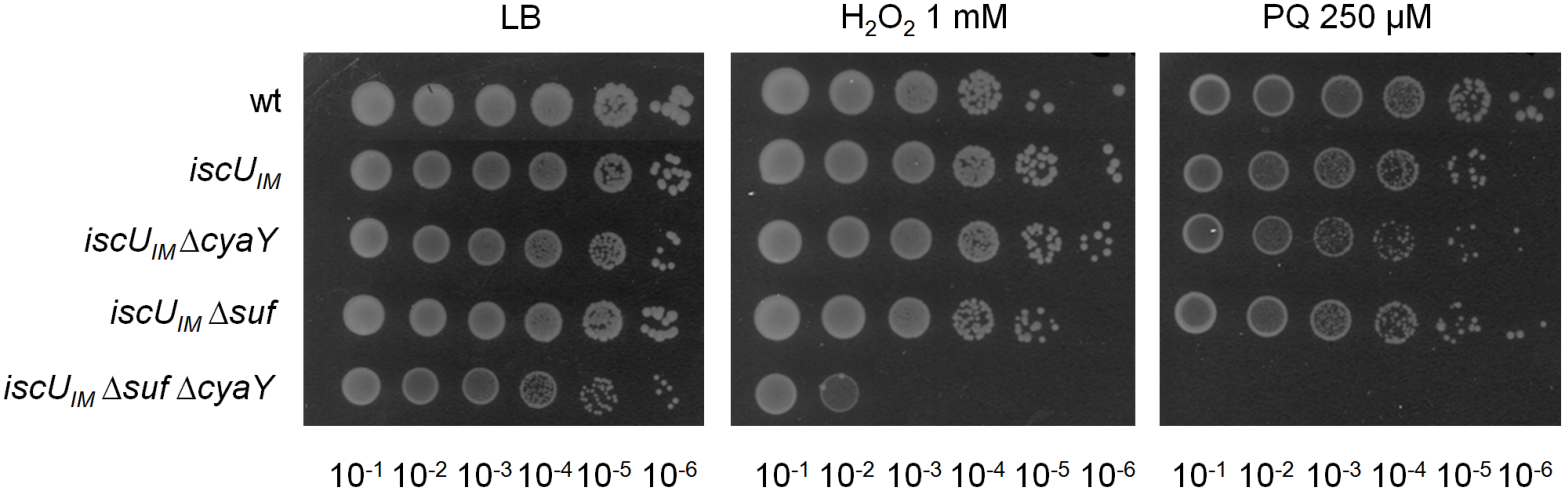
The *iscU*_*IM*_ Δ*suf* Δ*cyaY* strain is hypersensitive to oxidative stress. The wt (DV901), *iscU*_*IM*_ (BR755), *iscU*_*IM*_ *ΔcyaY* (BR756), *iscU*_*IM*_ *Δsuf* (BR763) and *iscU*_*IM*_ *Δsuf ΔcyaY* (BR767) strains were grown overnight at 37°C in LB medium. Cultures were diluted in sterile PBS, and 5 μL were directly spotted onto LB medium plates containing either 1 mM H_2_O_2_ or 250 μM paraquat. Growth was analysed after overnight incubation at 37°C. Each spot represents a 10-fold serial dilution.

Because frataxin deficiency, in yeast, led to hypersensitivity to oxidants, we also tested the importance of CyaY in the “eukaryotized” background. Fig 3 shows that introduction of the *cyaY* deletion in the *iscU*_*IM*_ Δ*suf* strain led to hypersensitivity to hydrogen peroxide and to paraquat, a superoxide generator.

Altogether, these results indicate that an *E*. *coli* lacking SUF can be turned into a frataxin-dependent organism simply by changing a single residue in the IscU scaffold.

### The *iscU*_*IM*_-associated defects are due to decreased Fe-S biogenesis

**Fig 4:**
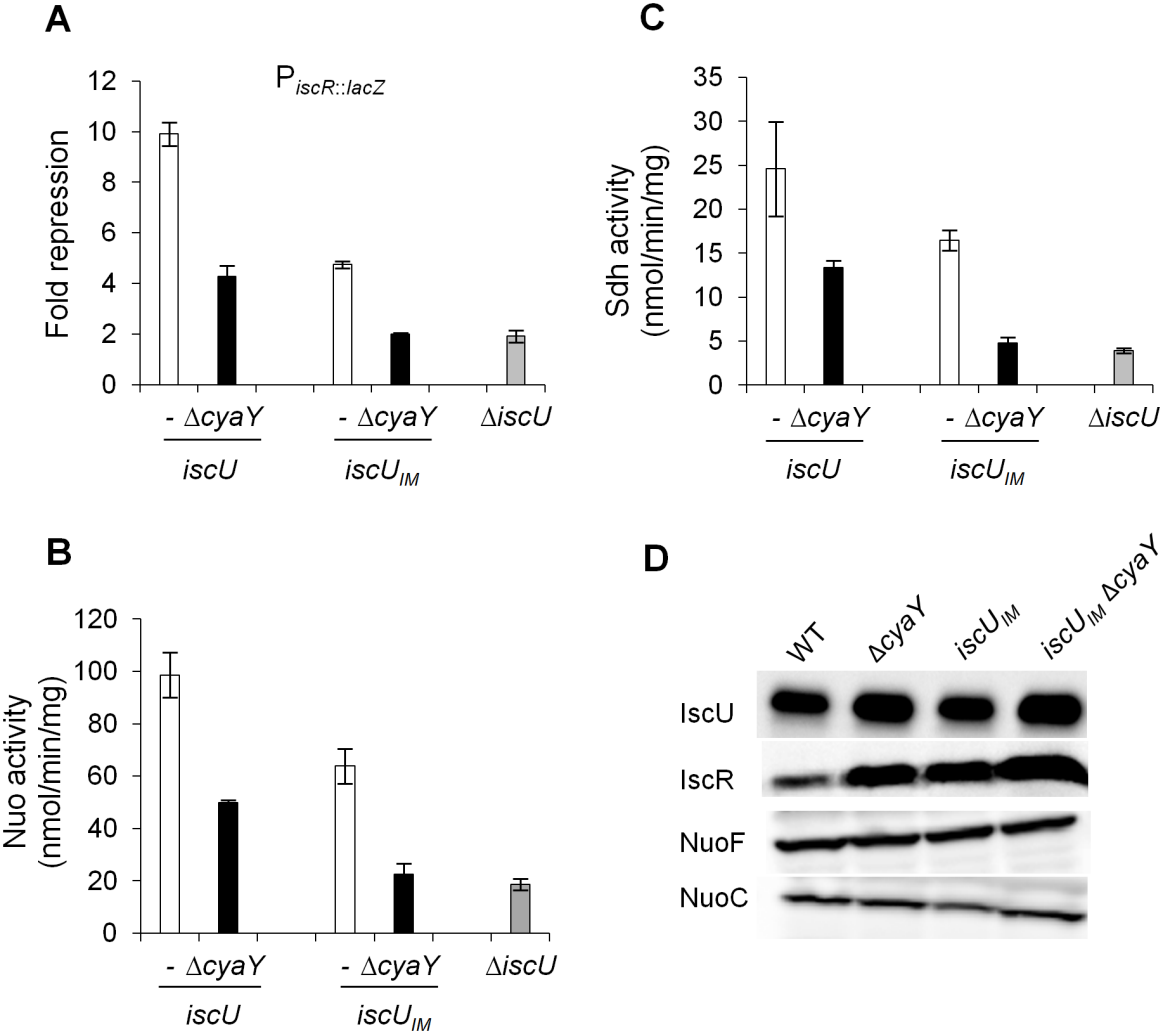
Activities of Fe-S proteins in *iscU*_*IM*_ and Δ*cyaY* strains. Repression of the IscR-regulated gene (*iscR*::*lacZ*) (A), Nuo (B) and Sdh (C) activities in the wt (DV901) (white bars), *iscU*_*IM*_ (BR755) (white bars), their *ΔcyaY* derivatives (DV925, BR756) (black bars), and Δ*iscU* (BR667) (grey bars) strains. The amount of IscR-dependent repression (fold repression) was determined by dividing the β-galactosidase activity present in the strain lacking IscR (DV915) by the β-galactosidase activity measured for each strain. Error bars represent the standard error from three independent experiments. (D) Cell extracts of indicated strains were subjected to immunoblot analysis using antibodies raised against IscU, IscR, NuoF and NuoC.

In order to ascertain that the drastic defects observed in the *iscU*_*IM*_ Δ*cyaY* strain were directly due to a dysfunction of Fe-S cluster biogenesis, we tested the activity of several Fe-S cluster-containing proteins. These latter were IscR, a [2Fe-2S] transcriptional regulator, Nuo and Sdh, two multi-protein complexes containing 9 and 3 Fe-S clusters, respectively. In full agreement with the phenotypic tests reported above, introduction of a Δ*cyaY* mutation in a strain synthesizing the eukaryote-like IscU_IM_ scaffold essentially recapitulated the effect of deleting the scaffold-encoding gene *iscU* (Fig 4A, 4B and 4C). As a point of comparison, in the *iscU*_*IM*_ strain, the IscR, Nuo and Sdh activities were decreased by 1.5–2 fold when compared to the wt strain (Fig 4A, 4B and 4C). Immunoblot analysis of the IscR, Nuo and IscU_IM_ proteins ruled out that the decreased activities were due to reduced amounts of target or scaffold proteins (Figs 4D and S2). Altogether, these results indicate that even though it is a conservative change, a single Ile-to-Met substitution in the IscU scaffold alters Fe-S biogenesis efficiency.

### The IscU_IM_ forms Fe-S cluster at a slower rate

**Fig 5:**
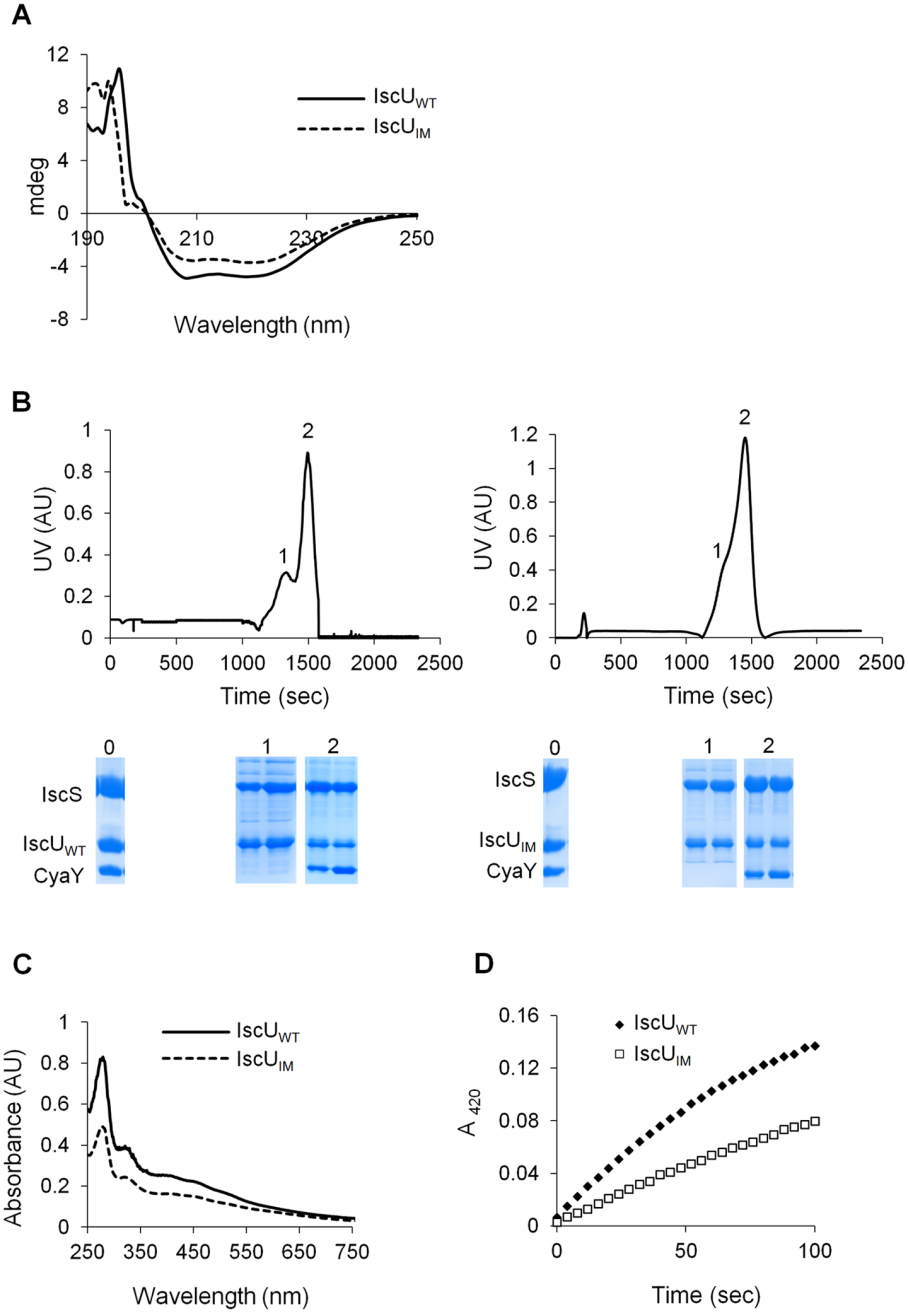
Analysis of IscU_IM_ *in vitro*. (A) Comparison of the CD spectra (expressed in mdeg) recorded in the region 190–250 nm between IscU_WT_ (filled line) and IscU_IM_ (dotted line). (B) Purified IscS, CyaY and IscU_WT_ (left panel) or IscU_IM_ (right panel) were mixed in 1:1:1 ratio (144 μM of each protein) in the presence of 4-fold excess of Fe(SO_4_)_2_(NH_4_)_2_, 10-fold excess of L-cysteine and 5 mM DTT and incubated for 40 minutes. The mixture was then loaded onto a QFF column equilibrated with 50 mM Tris pH 8. Proteins were eluted with 50 mM Tris pH 8 containing 1M NaCl. SDS-PAGE analyses have been performed on samples from the column on-put (0) and the peaks 1 and 2 for each mixture. (C) Reconstitution of [2Fe-2S] IscU_WT_ (filled line) and IscU_IM_ (dotted line) followed by UV-visible absorption spectroscopy. Apo-IscU_WT_ or apo-IscU_IM_ (144 μM) were incubated with 5 mM DTT, 1.44 μM IscS, 2 mM L-cysteine and 0.43 mM Fe(SO_4_)_2_(NH_4_)_2_ in 50 mM Tris-HCl pH 8. (D) Comparison of the kinetics of enzymatic Fe-S cluster formation on IscU_WT_ (black diamonds) and IscU_IM_ (white squares). Experiment was carried out using 25 μM IscU_WT_ or IscU_IM_, 25 μM IscS, 100 μM Fe(SO_4_)_2_(NH_4_)_2_, 250 μM L-cysteine, 2 mM DTT. Fe-S cluster formation was followed by absorbance at 420 nm. The experiment was repeated at least three times. One representative experiment is shown.

In order to understand the molecular basis for the effect caused by the mutation, the IscU_IM_ protein was submitted to a thorough *in vitro* analysis. A plasmid encoding a His-tagged IscU_IM_ was constructed, and the tagged protein was purified in large quantities. The CD spectra of IscU_IM_ and IscU_WT_ were similar, indicating that the mutation did not affect the secondary structure of the protein (Fig 5A). Also gel filtration experiments indicated that the IscU_IM_ formed dimers like the IscU_WT_ (S3 Fig).

The IscU_WT_ was previously shown to be isolated from complexes together with IscS and IscS-CyaY [23,24]. Therefore, we investigated whether the IscU_IM_ had similar behavior. To this purpose an anion exchange chromatography approach was used. Purified reconstituted IscU_WT_ or IscU_IM_ was mixed anaerobically with molar stoichiometric amount of IscS and CyaY proteins. The mixtures were loaded onto an anion exchange column (QFF), and the collected fractions were analysed by SDS-PAGE. Using IscU_WT_, a first peak (peak 1), containing IscU and IscS, eluted at 640 mM NaCl while a second major peak (peak 2), which eluted at 780 mM NaCl contained the IscS, IscU and CyaY proteins (Fig 5B left panel). The proteins recovered in peak 1 and peak 2 were part of a complex, since each individual protein, IscU_WT_, IscS and CyaY eluted from the column at 400, 430 and 530 mM NaCl, respectively (S4 Fig). A similar result was obtained when using IscU_IM_ instead of IscU_WT_ (Figs 5B right panel and S4). Thus, these data show that the ability of IscU to associate with IscS and CyaY was not altered by the Ile-to-Met mutation.

Lastly, we investigated whether IscU_IM_ could assemble a [2Fe-2S] cluster. Fig 5C shows that after anaerobic Fe-S cluster reconstitution, IscU_WT_ and IscU_IM_ displayed similar UV-vis. spectra characteristic of [2Fe-2S] clusters, with absorption maxima at 320, 410 and 456 nm (Fig 5C) [47–49]. However, the rate of Fe-S cluster formation differed between the two. Indeed the rate of Fe-S cluster formation was slowed down by approximately 2-fold when using the IscU_IM_ mutant (Fig 5D). Altogether, these biochemical investigations revealed that the Ile-to-Met mutation specifically altered the efficiency of Fe-S cluster formation on IscU_IM_, with no major effect on the structure of IscU_IM_ or its capacity to interact with its partners IscS and CyaY.

### Evolution of CyaY and IscU in prokaryotes

CyaY contains a single domain of ~100 residues referred to as PF01491 in the Pfam database. By using this domain as a query, we detected 598 homologous proteins within 2742 complete prokaryotic genomes available in the local bank of complete genomes (2 March, 2014) (S1 Table).

**Fig 6:**
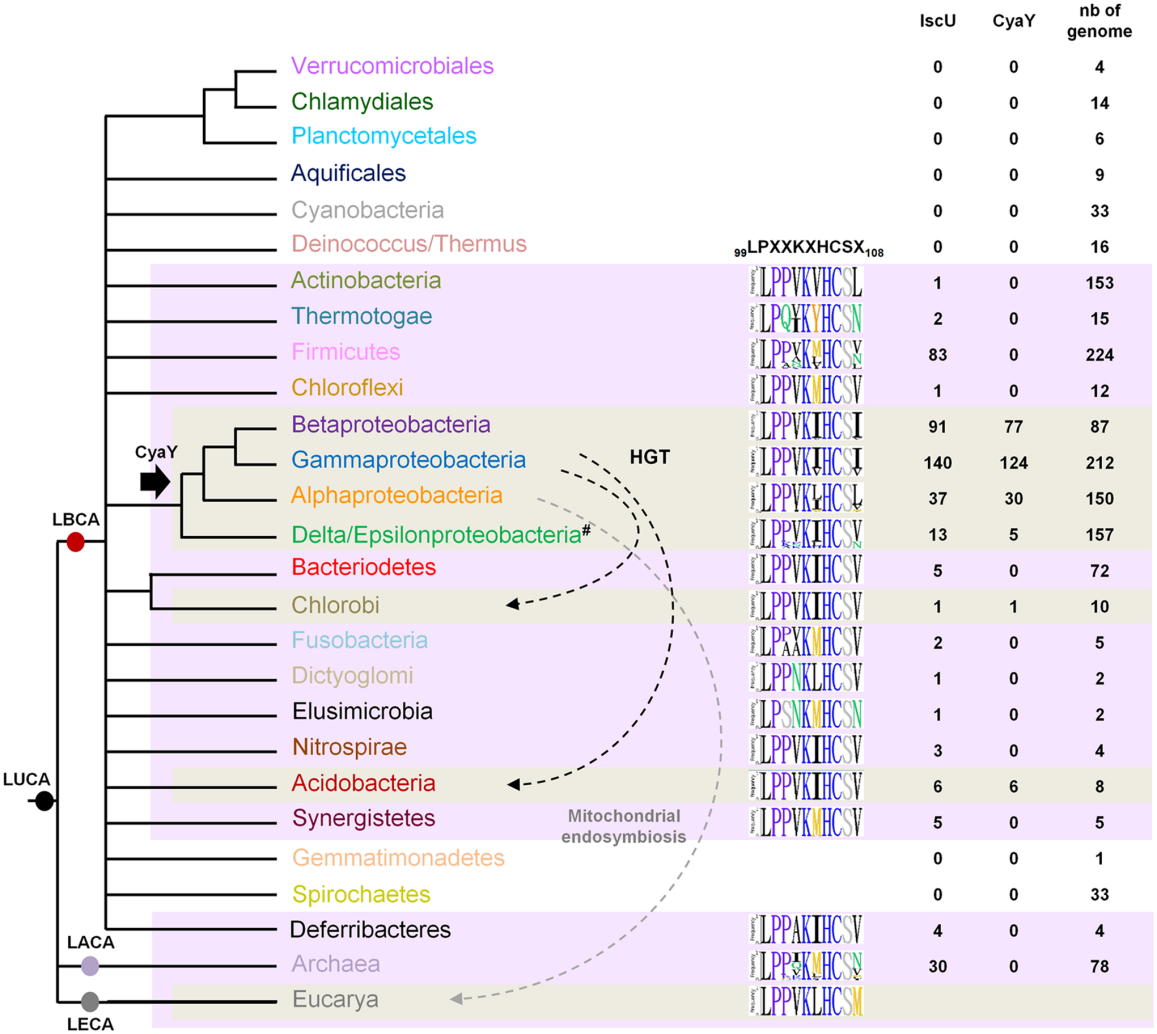
Model for CyaY protein evolution. Schematic representation of the universal tree of life, for which complete genome sequences are available. LUCA (Last Universal Common Ancestor), LECA (Last Eukaryotic Common Ancestor), LACA (Last Archaeal Common Ancestor) and LBCA (Last Bacterial Common Ancestor). For each prokaryotic phylum (whose color code is the same as the one used in S5 Fig), the number of genomes encoding a CyaY and a IscU homolog with respect to the number of complete available genomes is given. The black arrow indicates the presence of a CyaY encoding gene in the ancestor of a given lineage. The evolutionary event at the origin of the *cyaY* gene in the Delta/Epsilon subgroup cannot be definitively inferred. One hypothesis is that the *cyaY* gene is originated in the common ancestor of the Proteobacteria which together with a probable massive loss of *cyaY* (#) in Delta/Epsilonproteobacteria subgroup explains the presence of CyaY in the species of the Delta/Epsilonproteobacteria subgroup. Dotted arrows indicate horizontal gene transfer events (HGT) (black) and the mitochondrial endosymbiosis (grey). Sequence-logo of the region 99–108 in IscU homologs is also represented using Phylo-mLogo. This region contains the LPPVK motif and amino acid residues at position 108.

Homologs of CyaY were found in Alpha-, Beta-, Gammaproteobacteria, Acidobacteria and Deltaproteobacteria species, and in one representative of Chlorobi phylum (*Chloroherpeton thalassium ATCC 35110*) (Fig 6). These data indicate that CyaY is not widely distributed among prokaryotes. The absence of a CyaY encoding gene in the ancestor of most bacterial phyla suggests that a CyaY encoding gene was absent in LBCA. The phylogenetic analysis of CyaY also showed that the representatives of Chlorobi and Acidobacteria phylum, which emerge within the Gammaproteobacteria, have probably acquired *cyaY* gene by HGT (dotted black arrows) (Figs 6 and S5). Altogether, these results suggest that the CyaY protein originated in the bacterial domain, likely in the common ancestor of the Proteobacteria with massive loss in Delta/Epsilonproteobacteria subdivision.

IscU homologs were retrieved using the PF01592 domain and were aligned using the multiple alignment program MAFFT v7.045b (S1 Table). We imposed some additional criteria in order for a protein to be considered as an IscU homolog: (i) the presence of the three conserved cysteine residues that are required for the scaffold activity of IscU, (ii) the presence of the sequence that is recognized by the chaperone/co-chaperone system of the ISC system (LPPVK in *E*. *coli* IscU) (iii) no other additional domain such as those that could be found in NifU, and (iv) at least one other *isc*-related gene as a neighbor gene. Using these criteria, well studied U-like proteins such as the SufU protein of *Bacillus subtilis* and the NifU protein of *Azotobacter vinelandii* and their close homologs were eliminated. We then showed that all the prokaryotic species that possessed CyaY also contained an IscU homolog. However, the reverse situation was not true, since numerous prokaryotic species possessing IscU did not contain a CyaY encoding gene (Fig 6).

Sequence alignment of the 429 prokaryotic IscU homologs showed that amino acids at position 108 were mostly (369/429) Ile, Leu or Val (Figs 6 and S6). A few IscU examples exhibited a Met or Asn amino acid. Interestingly, an IscU_M_ protein was found in 3 out of 28 species of the Rickettsiales order (Alphaproteobacteria) (*Orientia tsutsugamushi* str. Boryong, YP_001248706; *Neorickettsia risticii* str. Illinois, YP_003081518 and *Neorickettsia sennetsu* str. Miyayama, YP_506192). An IscU_M_ protein was also found in two Archaea species (2 out of 30 species) *Methanosarcina barkeri* str. Fusaro (YP_305925), and *Methanosarcina acetivorans* C2A (NP_617616).

## Discussion

ISC machineries from both eukaryotes and prokaryotes are considered to be homologous. They share many components including cysteine desulfurases, scaffolds, dedicated-chaperone proteins and A-type carriers. A defect in any of these conserved components provokes a drastic drop in Fe-S cluster biogenesis in either eukaryotes or prokaryotes [12–14,26–29,50,51]. The case of frataxin is different, however, as a lack of FXN in eukaryotes, humans or yeast, is markedly more detrimental than a lack of CyaY in prokaryotes such as *E*. *coli* or *Salmonella* [30–44]. A possible explanation for the difference is that variation within the Fe-S cluster assembly machineries provides different contexts, which in turn make the contribution of FXN of greater importance than that of CyaY. In this regard, it is important to recall that the core eukaryotic ISC system includes a component, Isd11, which interacts with Nfs1 [52–54]. A model was recently proposed according to which the eukaryotic Nfs1 cysteine desulfurase remains in an OFF state unless it interacts with FXN and Isd11 [55–58]. However, no Isd11-like proteins are present in *E*. *coli* and this regulation of IscS activity does not apply [52,53].

Recent genetic analysis by the Dancis group showed that modifying part of the ISC machinery could render it independent of FXN. Indeed, in a search for suppressing mutation that could bypass the lack of FXN, these authors identified a mutation in the scaffold-encoding gene *ISU1* [45]. The suppressing mutation allowed Isu1 to activate Nfs1, thereby mimicking FXN [55,59]. Remarkably, this mutation changed a Met residue, conserved in eukaryotes, to an Ile residue, conserved into prokaryote IscU proteins. Although largely speculative, this result may open the way to deciphering the contribution of frataxin in the functioning of ISC machineries, and possibly provide a lead towards understanding the differences between prokaryotes and eukaryotes.

In the present work, we carried out a bioinformatic analysis of IscU sequences in prokaryotes. This allowed us to confirm that position 108 was mostly occupied by Ile, as in *E*. *coli*, Leu or Val. By contrast, position 108 in prokaryotes was almost never occupied by Met (see below for an exception), which is the situation most frequently encountered in eukaryotes. In an effort to address the importance of this residue experimentally, the Ile residue was changed to Met at position 108 of IscU and expressed into *E*. *coli* lacking SUF. The results confirmed the influential role of that position. First, the *E*. *coli* strain containing a eukaryotic-like IscU_IM_ became fully dependent on CyaY. Thus, this strain was unable to mature a series of Fe-S cluster containing proteins such the transcriptional regulator IscR, a [2Fe-2S] protein, or Nuo and Sdh, multi-cluster containing enzymes of the electron transport chain. Moreover, such a strain became auxotrophic for various amino acids, including Ile, Leu and Val, the branched amino acids whose synthesis depends on the Fe-S cluster containing proteins, dihydroxy-acid dehydratase (IlvD) and isopropylmalate dehydratase (LeuD). In addition, the strain showed hypersensitivity to oxidative stress, a phenotype linked to FXN deficiency in eukaryotes [39,60,61]. Therefore, a single Ile-to-Met substitution was sufficient to turn *E*. *coli* into a frataxin-dependent organism for Fe-S cluster biogenesis.

How could a single conservative Ile-to-Met change have such a crucial impact on Fe-S cluster biogenesis? A hypothesis is that the Ile-to-Met mutation alters the IscU protein, diminishing its efficiency in contributing to the overall Fe-S cluster biogenesis process and that in this context, the contribution of CyaY becomes essential. To test this hypothesis, we carried out a thorough biochemical characterization of the IscU_IM_ variant and could rule out structural or stability defects. This fits with the *in vivo* observation that the IscU_IM_ protein was as abundant as the wt protein and failed to exhibit instability as assessed by immunoblot analysis. Moreover, we observed that the IscU_IM_ protein interacted with its natural partners, IscS and CyaY, in a mode indistinguishable from the wild type. In contrast, *in vitro*, IscU_IM_ was found to assemble Fe-S clusters at a rate 2-fold slower than the wild type. Interestingly, these data are consistent with the *in vivo* observation that *E*. *coli* containing a chromosomal copy of the mutated *iscU* allele was 2-fold less efficient in maturing IscR than the wt strain. Hence, altogether these results support the notion that the Ile-to-Met mutation altered the kinetic formation of Fe-S clusters on IscU. A possible structural explanation for the effect might be that the mutation modifies the accessibility of sulfur or iron for Fe-S cluster intermediate formation, as the 108^th^ position is in close vicinity to the Cys106, one of the three Cys residues acting as ligands. Regardless of the structural basis for this effect, the fact is that this analysis revealed that a eukaryotic-like IscU is slightly less efficient than the *E*. *coli* one in assembling a cluster and as a consequence, the contribution of frataxin becomes more significant for helping the process to go on. As previously shown, we observed that *via* its interaction with IscS, CyaY slowed down the kinetic of Fe-S cluster formation on IscU (S7 Fig) [22,62]. At first this contradicts the view of CyaY acting as a positive effector for Fe-S cluster formation and this has already been discussed at length in the literature [22,62,63]. But what matters here is that CyaY also inhibited, and to the same extent, Fe-S cluster assembly by the IscU_IM_ variant (S7 Fig). This indicated that the CyaY action is not strictly connected to the nature of the residue at position 108. Thus one possibility is that CyaY and IscU_IM_ influence the overall Fe-S cluster biogenesis process at different steps. The fact that the CyaY dependency is not bypassed by increasing amount of the IscU_IM_ scaffold, as indicated *in vivo* by the CyaY-dependent maturation of IscR when IscU_IM_ was overproduced (S8 Fig), is consistent with this hypothesis. Further biochemical analyses are needed to investigate the possible sites of action in the Fe-S cluster assembly process, such as iron donation, control of sulfur flux, Fe-S cluster transfer to downstream recipients, or HscBA-associated steps, for the CyaY and IscU_IM_ effect.

The involvement of CyaY in Fe-S cluster biogenesis was proposed in the early 2000’s on the basis of co-occurrence of *cyaY* and *hscBA* genes [64]. This led to the belief that CyaY would be as conserved as the other ISC components. The reason why the cognate structural gene was not part of the *isc* operon in bacteria remained unclear. Here, exploiting the larger number of genomes now available for analysis, we reinvestigated the distribution of CyaY and its co-occurrence with the ISC system. Surprisingly, CyaY was found to be much less conserved in eubacteria than previously thought, as its presence was mostly restricted to Alpha-, Beta-, and Gammaproteobacteria. Interestingly, in these bacteria, none of the genes encoding components related to Fe-S cluster biogenesis were to be found in the vicinity of *cyaY*. Phylogenic analysis revealed that CyaY originated in the last common ancestor of Proteobacteria. This contrasts with the story for A-type Fe-S cluster carriers, which we previously found to be present in the last bacterial common ancestor [65,66]. Even more surprising was the fact that many genomes contained *iscU* but not *cyaY*, suggesting that these bacteria learned how to make Fe-S clusters in an ISC-dependent and CyaY-independent way. In contrast, all genomes containing *cyaY* also contained *iscU*. Hence overall this leads to picture CyaY as a Fe-S cluster biogenesis factor associated with the ISC machinery in most eukaryotes and in a restricted number of prokaryotes. Interestingly, not only some lineages such as Deltaproteobacteria, but also some species within the Alpha- and Betaproteobacteria have lost CyaY, indicating that there might have been some evolutionary drift favoring organisms that evolve without it. Amino acids encoded by the codon at position 108 of IscU are essentially Ile, Leu or Val. Methionine appears in only two cases, in Methanobacteria and some *Rickettsiae* species that also have a *cyaY* gene. *Rickettsiae* are thought to have given rise to mitochondria *via* the first endosymbiosis event. Hence, it is tempting to speculate that the current mitochondrial Isu protein originated from the IscU_M_ version that was already present in the ancestor of *Rickettsiae*.

Based upon the above considerations, one can envision the following scenario: i) Frataxin appeared in the ancestor of the Proteobacteria, and joined the ISC system for Fe-S cluster biogenesis, ii) Mitochondria developed from Proteobacteria by endosymbiosis, in particular from *Rickettsiae*, acquiring components what would give rise to the actual Isu_M_ and FXN, iii) Proteobacteria acquired SUF, which released the pressure on ISC, and in parallel they explored variation in the ISC scaffold at position 108. In particular, the Met-to Ile, Leu, Val changes happened to improve Fe-S cluster assembly, iv) Frataxin dependency was loosened in Proteobacteria that have a more efficient ISC scaffold and other Fe-S back up system.

## Materials and Methods

### Bacterial strains and growth conditions

The *E*. *coli* K-12 strain MG1655 and its derivatives used in this study are listed in Table 1. Deletion mutations from the KEIO collection were introduced by P1 transduction [67]. Transductants were verified by PCR, using primer pairs hybridizing upstream and downstream of the deleted gene. Strain BR755 producing the IscU_I108M_ variant from a chromosomal copy was constructed as follows: a DNA fragment carrying the *iscU*_*I108M*_ allele was obtained after a mutagenesis procedure by overlap extension PCR reactions using the following primer pairs: IscU-UPBamH1/IscU_I108M_-DO, IscU_I108M_-UP/IscU-DOXbaI, IscU-UPBamH1/IscU-DOXbaI (S2 Table). This DNA fragment was introduced in a strain in which the *iscU* gene had been replaced by a *cat-sacB* cassette as previously described [68]. The Suc-resistant clones were checked for Cm sensitivity, and the appropriate region was sequenced. The *iscU*_*I108M*_ allele was transduced into desired strains by using a Kan^R^-linked marker in the *yphD* gene, which is located close to the *iscU* gene. The Δ*suf* mutation was introduced in the *iscU*_*IM*_ background strains that contained the eukaryotic Fe-S cluster independent mevalonate pathway for IPP biosynthesis (MVA), in case the combination of *iscU*_*IM*_ Δ*cyaY* would have been lethal [50,51]. This precaution proved to be unnecessary since the *iscU*_*IM*_ Δ*suf* and *iscU*_*IM*_ Δ*suf* Δ*cyaY* strains could be obtained without the addition of arabinose and mevalonate. Addition of arabinose and mevalonate did not improve growth of the *iscU*_*IM*_ Δ*suf* and *iscU*_*IM*_ Δ*suf* Δ*cyaY* strains; therefore, all the experiments have been done without. However, the *iscU*_*IM*_ Δ*suf* and *iscU*_*IM*_ Δ*suf* Δ*cyaY* strains are auxotroph for tryptophan since the MVA synthetic operon was inserted in the *trp* operon, therefore when grown in M9 glucose minimal medium tryptophan was added [50].

**Table 1:** Bacterial strains and plasmids used in this study.

Strain or plasmid	Relevant genotype	Reference or source
*E*. *coli* K-12 strains		
MG1655	Parental strain	Lab collection
DV901	*lacIpoZ Δ* (*Mlu*) λ -P_*iscR*_ *-lacZ*	[51]
DV915	*lacIpoZ Δ* (*Mlu*) λ -P_*iscR*_ *-lacZ ΔiscR*::*kan*	[51]
DV925	*lacIpoZ Δ* (*Mlu*) λ -P_*iscR*_ *-lacZ ΔcyaY*	Lab collection
BR667	*lacIpoZ Δ* (*Mlu*) λ -P_*iscR*_ *-lacZ ΔiscU*	This study
DV1093	MG1655 MVA^+^	[51]
BR755	*lacIpoZ Δ* (*Mlu*) λ -P_*iscR*_ *-lacZ iscU*_*I108M*_ *ΔyphD*	This study
BR756	*lacIpoZ Δ* (*Mlu*) λ -P_*iscR*_ *-lacZ iscU*_*I108M*_ *ΔyphD ΔcyaY*	This study
BR757	*lacIpoZ Δ* (*Mlu*) λ -P_*iscR*_ *-lacZ iscU*_*I108M*_ *ΔyphD MVA* ^*+*^::*kan*	This study
BR763	*lacIpoZ Δ* (*Mlu*) λ -P_*iscR*_ *-lacZ iscU*_*I108M*_ *ΔyphD MVA* ^*+*^::*kan Δsuf*::*cat*	This study
BR767	*lacIpoZ Δ* (*Mlu*) λ -P_*iscR*_ *-lacZ iscU*_*I108M*_ *ΔyphD MVA* ^*+*^::*kan Δsuf*::*cat ΔcyaY*::*spec*	This study
PM12.05	MG1655 *mal*::*lacI* ^*q*^, *ΔaraBAD*, *lacI’*::*P*_*BAD*_ *-cat-sacB*:*lacZ*, *mini* λ *tet* ^*R*^	[68]
Plasmids		
pET22b-CyaY	pET22b expressing CyaY-(his)_6_	[85]
pQE-30-IscS	pQE30 expressing (his)_6_-IscS	[74]
pET21a-IscU	pET21a expressing IscU-(his)_6_	This study
pET21a-IscU_IM_	pET21a expressing IscU_I108M_-(his)_6_	This study

Oligonucleotides used in this study are listed in S2 Table. Supplementary strains are listed in S3 Table.

*E*. *coli* strains were grown at 37°C in Luria—Bertani (LB) rich medium or in minimal medium (M9) supplemented with glucose (0.4%) and MgSO_4_ (1 mM). Arabinose (0.2%), amino acids (0.5 mM), sucrose (5%), thiamine (0.2 μg/mL) and nicotinic acid (12.5 μg/mL) were added as required. Solid media contained 1.5% agar. Antibiotics were used at the following concentrations: chloramphenicol 25 μg/mL, kanamycin 30 μg/mL, tetracycline 25 μg/mL, gentamicin 5 μg/mL and ampicillin 50 μg/mL.

### Plasmid construction

Plasmid pIscU was constructed by PCR amplification of the coding region of *iscU* from *E*. *coli* MG1655 chromosomal DNA using the following primer pair: NcoI-IscU/HindIII-IscU (S2 Table). The PCR product was then digested by *Nco*I and *Hind*III and cloned into the *Nco*I/*Hind*III linearized pBAD24 vector.

Production of the IscU_IM_ variant exhibiting a single amino acid substitution isoleucine to methionine at position 108 was obtained by site-directed mutagenesis in the pIscU plasmid to generate pIscU_IM_ using the following primer pair: IscU_I108M__for/IscU_I108M__rev (S2 Table).

Plasmids pETIscU_WT_ and pETIscU_IM_ were constructed by PCR amplification of the coding region of *iscU* from *E*. *coli* MG1655 chromosomal DNA and from the pIscU_IM_ vector, respectively, using the following primer pair: *Nde*I-IscU/*Hind*III-IscU (S2 Table). The PCR products were then digested by *Nde*I and *Hind*III and cloned into the *Nde*I/*Hind*III linearized pET21a+ vector.

Plasmids pET22b-CyaY and pQE-IscS for production of recombinant *E*. *coli* CyaY and IscS, were described previously [69].

### Generation of survival curves

Overnight cultures were diluted and grown aerobically in LB at 37°C to an OD_600_ of 0.2. At this point, antibiotics were added to the cells (Gm at 5 μg/mL and Kan at 10 μg/mL). At different incubation times, 100 μL of cells were diluted in PBS buffer, spotted on LB agar and then incubated at 37°C overnight. Cell survival was determined by counting colony-forming units per mL (CFU/mL). The absolute CFU at time-point 0 (used as the 100%) was ≈ 5x10^7^ CFU/mL.

### Paraquat and hydrogen peroxide sensitivity test

Overnight cultures were diluted in sterile PBS and 5 μL were directly spotted onto LB plates containing either paraquat (250 μM) or H_2_0_2_ (1 mM). The plates were incubated overnight at 37°C before growth was scored.

### β-Galactosidase assay

Strains were grown at 37°C in LB rich medium, to an OD_600_ of ~1.5. β-galactosidase assays were carried out as previously described [70].

### Enzymatic assays

#### NADH dehydrogenase activity

NADH dehydrogenase activity was assayed as previously described [71]. Briefly, cells were grown to an OD_600_ of 0.6–0.8, harvested by centrifugation, resuspended in MES-10% glycerol buffer pH 6.5, and disrupted in a French press. Aliquots of the whole-cell extract were immediately frozen in liquid nitrogen and stored at -80°C until used. Enzymatic activity was measured spectrophotometrically at 30°C by following absorbance at 340 nm in a reaction mixture containing 50 mM MES, pH 6.5, 10% glycerol and 200 μM D-NADH, as a specific substrate. Protein concentration was determined using the protein A_280_ method on NanoDrop2000 spectrophotometer.

#### Succinate dehydrogenase activity

Succinate dehydrogenase activity (Sdh) was assayed as described previously [71]. Briefly, cells were grown to an OD_600_ of 0.6–0.8, harvested by centrifugation, resuspended in MES-10% glycerol buffer pH 6.5, and disrupted in a French press. Following centrifugation (11 000 rpm for 15 min at 4°C), the supernatant was submitted to ultracentrifugation (45 000 rpm for 2 h at 4°C) to obtain the membrane fraction. Sdh activity was assayed for the pellet fraction resuspended in MES-10% glycerol buffer pH 6.5. Because Sdh is partially inhibited by oxaloacetate, the enzyme was first activated by incubation in 50 mM Tris-HCl pH 7.5, 4 mM succinate and 1 mM KCN for 30 min at 30°C [72,73]. Sdh activity was then measured spectrophotometrically at 30°C by following the phenazine ethosulfate (PES)-coupled reduction of DCPIP at 600 nm, in a reaction mixture containing 50 mM Tris-Hcl pH 7.5, 4 mM succinate, 1 mM KCN, 400 μM PES and 50 μM DCPIP. Protein concentration was determined using a NanoDrop2000 spectrophotometer to determine the protein A_280_.

### Western blot analysis

Equal quantities of protein were applied to SDS-PAGE and transferred onto nitrocellulose membranes. The membrane filters were incubated with appropriate antibodies (1/200, 1/2000, 1/2000, 1/150 dilutions of the anti-IscU, anti-NuoF, anti-NuoC and anti-IscR serums, respectively). Immunoblots were developed by using horseradish peroxidase-conjugated goat anti-rabbit antibody, followed by chemiluminescence detection.

### Expression and purification of proteins

Recombinant CyaY, IscU_WT_ and IscU_IM_ proteins containing a C-terminal His_6_ tag were expressed in *E*. *coli* and purified as follows: *E*. *coli* BL21 (DE3)/pET*cyaY* was grown in LB medium containing 50 μg/mL ampicillin at 37°C. Protein expression was induced for 4 h by the addition of 0.5 mM isopropyl β-D-thiogalactoside (IPTG) at an OD_600_ ≈ 0.5. The bacterial pellet was resuspended in buffer A (0.1 M Tris-HCl, pH 8, 500 mM NaCl, 20 mM imidazole) and disrupted in a French press. After centrifugation (15 min, 11 000 rpm, 4°C), the supernatant was loaded onto a 1-mL HisTrap affinity column (GE Healthcare) equilibrated with buffer A. Proteins were eluted with a gradient of buffer A containing 500 mM imidazole. Protein-containing fractions were desalted with a Nap-25 column (Amersham Biosciences) and then concentrated. A similar procedure was used to purify IscU_WT_ and IscU_IM_ proteins except that protein expression was induced by the addition of 1 mM IPTG. Recombinant *E*. *coli* IscS containing an N-terminal His_6_ tag was expressed and purified as previously described [74]. The protein concentration was estimated by measuring the absorbance at 280 nm with the NanoDrop2000 spectrophotometer and using the calculated molar extinction coefficient.

### Circular dichroism experiments

CD spectra were recorded on a Jasco J-815 spectropolarimeter by using Hellma 110-QS cuvettes of 1 mm path length. CD measurements were performed in 50 mM Tris-HCl pH 8, 50 mM NaCl using protein concentrations of 2 μM. 20 scans were averaged and the buffer baseline was subtracted.

### *In vitro* Fe-S reconstitution

The purified His-tagged IscU_WT_ and IscU_IM_ proteins were obtained in the apo-form. The purified proteins were reconstituted anaerobically in a glove box as described previously [48]. Briefly, 144 μM protein was mixed with 5 mM DTT, 1.44 μM IscS, 2 mM L-cysteine and 0.43 mM Fe(SO_4_)_2_(NH_4_)_2_ in a total volume of 500 μL of buffer A (50 mM Tris-HCl pH 8). Formation of Fe-S clusters on IscU was followed by UV-visible absorption spectroscopy using a Cary 1 Bio spectrophotometer. After 3 h incubation, samples were loaded onto a 1-mL anion exchange column (QFF) (GE Healthcare) equilibrated with buffer A and eluted with a gradient of buffer A containing 1 M NaCl. Protein fractions were concentrated on a Microcon concentrator (Amicon) and each concentrate was analysed for its Fe content, and for its UV-visible spectrum.

### Ion exchange chromatography

Purified His-tagged IscU_WT_ or IscU_IM_, IscS and CyaY proteins were mixed anaerobically in a 1:1:1 ratio (144 μM of each protein) for 40 minutes with 4-fold excess of Fe(SO_4_)_2_(NH_4_)_2_, 10-fold excess of L-cysteine and 5 mM DTT in a total volume of 500 μL of buffer A (50 mM Tris-HCl pH 8). The mixture was loaded onto a 1-mL QFF column (GE Healthcare) equilibrated with buffer A and eluted with a gradient of buffer A containing 1 M NaCl. Proteins elution was visualized by SDS-PAGE.

### Kinetics of Fe-S formation

To assess kinetics of cluster formation on IscU_WT_ or IscU_IM_, absorbance at 420 nm was measured as a function of time. 25 μM IscU_WT_ or IscU_IM_ was incubated anaerobically with 100 μM Fe(SO_4_)_2_(NH_4_)_2_, 2 mM DTT in 50 mM Tris-HCl pH 8. Subsequently, 25 μM IscS and 250 μM L-cysteine were added to start the reaction.

### Bioinformatic and phylogenomic analyses

The 2742 complete prokaryotic proteome (2591 bacterial and 151 archaeal) available at the NCBI in March 03, 2014 were downloaded (ftp://ftp.ncbi.nlm.nih.gov/genomes/). The HMMER package v3.0b2 and self-written scripts were then used to search for CyaY homologs in these complete genomes, requiring the presence of Frataxin-like domain (PFAM accession number PF01491) [75]. Alignments E-value with the 599 profile less than 0.1 were considered as significant. To retrieve CyaY sequence we imposed homology with the entire CyaY sequence and an E-value with 1.7e-7 as threshold. In addition, alignments have been visually inspected. Proteins of the YjbR family, such as YdhG from *Bacillus subtilis* have not been detected since despite their structural similarity with CyaY they lack similarity at the sequence level [76,77]. The corresponding sequences were subsequently analysed with the same software in order to determine the presence of additional known functional domains. Additional BLASTP/tBLASTN searches were performed in complete genomes to ensure that the CyaY family was exhaustively sampled and in the nr database at the NCBI to retrieve eukaryotic sequences [78]. For each homolog, the gene context, defined as the 5 neighboring genes located upstream and downstream, was investigated using MGcV (Microbial Genomic context Viewer) [79].

The retrieved homologous sequences were aligned using MAFFT v7.045b [80]. The best resulting alignment was then visually inspected and manually refined using ED program from the MUST package [81]. The regions in a multiple sequence alignment that were suited for phylogenetic inference were selected by using BMGE (BLOSUM30 similarity matrix) [82].

The phylogeny of all the prokaryotic CyaY was constructed using both maximum likelihood (ML) and Bayesien methods. ML analyses were run using PHYML version 3.1 with the Le and Gascuel (LG) model (amino acid frequencies estimated from the dataset) and a gamma distribution (4 discrete categories of sites and an estimated alpha parameter) to take into account evolutionary rate variations across sites [80]. The robustness of each branch was estimated by the non-parametric bootstrap procedure implemented in PhyML (100 replicates of the original dataset with the same parameters). Bayesian analyses were performed using MrBayes version 3.2.2 with a mixed model of amino acid substitution including a gamma distribution (4 discrete categories) and an estimated proportion of invariant sites [83]. MrBayes was run with four chains for 1 million generations and trees were sampled every 100 generations. To construct the consensus tree, the first 1500 trees were discarded as ‘‘burnin”.

For the dataset construction IscU homologs was retrieved from complete proteome available in the local databank (see above) using BLASTP. The distinction between homologous and non-homologous sequences was assessed by visual inspection of each BLASTP outputs (no arbitrary cut-off on the E-value or score). We imposed some additional criterion in order for a protein to be considered as an IscU homologs: the presence of the three conserved cysteine residues that are required for the scaffold activity of IscU, no other additional domain such as those that could be found in NifU, and at least one other *isc*-related gene as a neighbor gene. The IscU homologs were gathered in a dataset and the corresponding sequences were aligned using MAFFT v7.045b [80].

Sequence-logo of IscU alignment was generated using Phylo-mLogo visualization tool in order to highlight the LPPVK motif and residues in position 108 [84].

Additional materials and methods are mentioned in S1 Text.

## Supporting Information

S1 Fig Addition of thiamine and nicotinic acid rescues partially the residual growth defect exhibited by the *iscU*_*IM*_ Δ*suf* Δ*cyaY* strain. Growth of the *iscU*_*IM*_ **Δ** *suf* (BR763) (diamonds) and *iscU*_*IM*_ *Δsuf ΔcyaY* (BR767) (squares) strains in glucose M9 minimal medium supplemented with all amino acids and complemented with (white symbols) or without (black symbols) thiamine (B1) and nicotinic acid (NA). Growth was monitored at 600 nm. The experiment was repeated at least three times. One representative experiment is shown.
(TIF)

S2 Fig Quantification of western blots analysis. Quantification of western blots analysis of results shown in Fig 4 was performed using ImageQuantTL software.
(TIF)

S3 Fig IscU_WT_ and IscU_IM_ exist mainly as dimers. Comparison of the elution profiles between IscU_WT_ (A) and IscU_IM_ (B). For each protein, a gel filtration was performed on a Superdex 75 10/300 GL equilibrated with buffer A (0.1 M Tris-HCl pH 8, 50 mM NaCl). (C) Oligomerization state of IscU_WT_ and IscU_IM_ was determined from calibration curve using ribonuclease A (A; 13.7 kDa), chymotrypsinogen A (B; 25 kDa) and ovalbumin (C, 43 kDa) as molecular standards. Values of the elution volume (Ve)/ void volume (V0) are given for IscU_WT_ and IscU_IM_.
(TIF)

S4 Fig Chromatographic profiles on anion exchange QFF of the mixture and single purified proteins. For each profile obtained from the mixtures of IscU_WT_/IscS/CyaY (A) and IscU_IM_ /IscS/CyaY (E), the black arrows indicate the elution for each single protein (U: IscU_WT/IM_; S: IscS; C: CyaY) whose chromatographic profiles are shown below: IscU_WT_ (B), IscS (C), CyaY (D), and IscU_IM_ (F). Equilibration buffer of QFF column is 50 mM Tris-HCl pH 8 and elution was performed with a gradient of 50 mM Tris-HCl, pH 8, 1M NaCl. Flow rate: 1 mL/min.
(TIF)

S5 Fig CyaY phylogenetic tree. Unrooted Bayesian phylogenetic trees of CyaY (251 sequences, 70 positions). Numbers at nodes indicate posterior probabilities (PP) computed by MrBayes and bootstrap values (BV) computed by PhyML. Only PP and BV above 0.5 and 50% are shown. The scale bars represent the average number of substitutions per site. In the phylogenetic tree each prokaryotic phylum is highlighted in different colors: Alphaproteobacteria (orange), Gammaproteobacteria (blue), Deltaproteobacteria (green), Chlorobi (grey), Acidobacteria (red), Betaproteobacteria (purple). This color code is the same as the one used in the Fig 6.
(PDF)

S6 Fig Alignment of the prokaryotic IscU. IscU homologs were aligned using MAFFT v7.045b. The _99_LPPVK_103_ motif and the residue at position 108 are indicated at the top of the alignment. Species having Methionine at position 108 are highlighted in yellow.
(PDF)

S7 Fig Inhibitory effect of CyaY on Fe-S cluster formation on IscU_WT_ and IscU_IM_ *in vitro*. Comparison of the kinetics of enzymatic Fe-S cluster formation on IscU_WT_ (black diamonds; red triangles) and IscU_IM_ (white circles; white squares) with (red triangles; white circles) or without (black diamonds; white squares) CyaY. Experiment was carried out using 25 μM IscU_WT_ or IscU_IM_, 25 μM IscS, 25 μM CyaY,100 μM Fe(SO_4_)_2_(NH_4_)_2_, 250 μM L-cysteine, 2 mM DTT. Fe-S cluster formation was followed by absorbance at 420 nm. The experiment was repeated at least three times. One representative experiment is shown.
(TIF)

S8 Fig Overproduction of IscU_IM_ does not alleviate the CyaY requirement. Repression of the IscR-regulated gene (*iscR*::*lacZ*) in the Δ*iscU* (BR667) mutant (A) and the Δ*cyaY* Δ*iscU* (BR668) mutant (B) transformed with pBAD (empty vector) (white bars), pIscU (black bars) or pIscU_IM_ (grey bars) plasmids. Cultures were grown in LB medium supplemented with ampicillin and arabinose. The amount of IscR-dependent repression (fold repression) was determined by dividing the β-galactosidase activity present in the strain lacking IscR (DV915) by the β-galactosidase activity measured for each strain. Error bars represent the standard error from three independent experiments.
(TIF)

S1 Text Materials and methods. (DOCX)

S1 Table List of the homologs of the CyaY and IscU proteins found in complete genomes. For each gene the accession number is provided.
(XLSX)

S2 Table List of the oligonucleotides used in this study. (DOCX)

S3 Table List of supplementary bacterial strain and plasmids used in this study. (DOCX)
