# Supplementary figures and images for: Turning Escherichia coli into a Frataxin-Dependent Organism
Source: PLoS Genet. 2015 May 21;11(5):e1005134. doi: 10.1371/journal.pgen.1005134 (PMC4440780; doi:10.1371/journal.pgen.1005134)

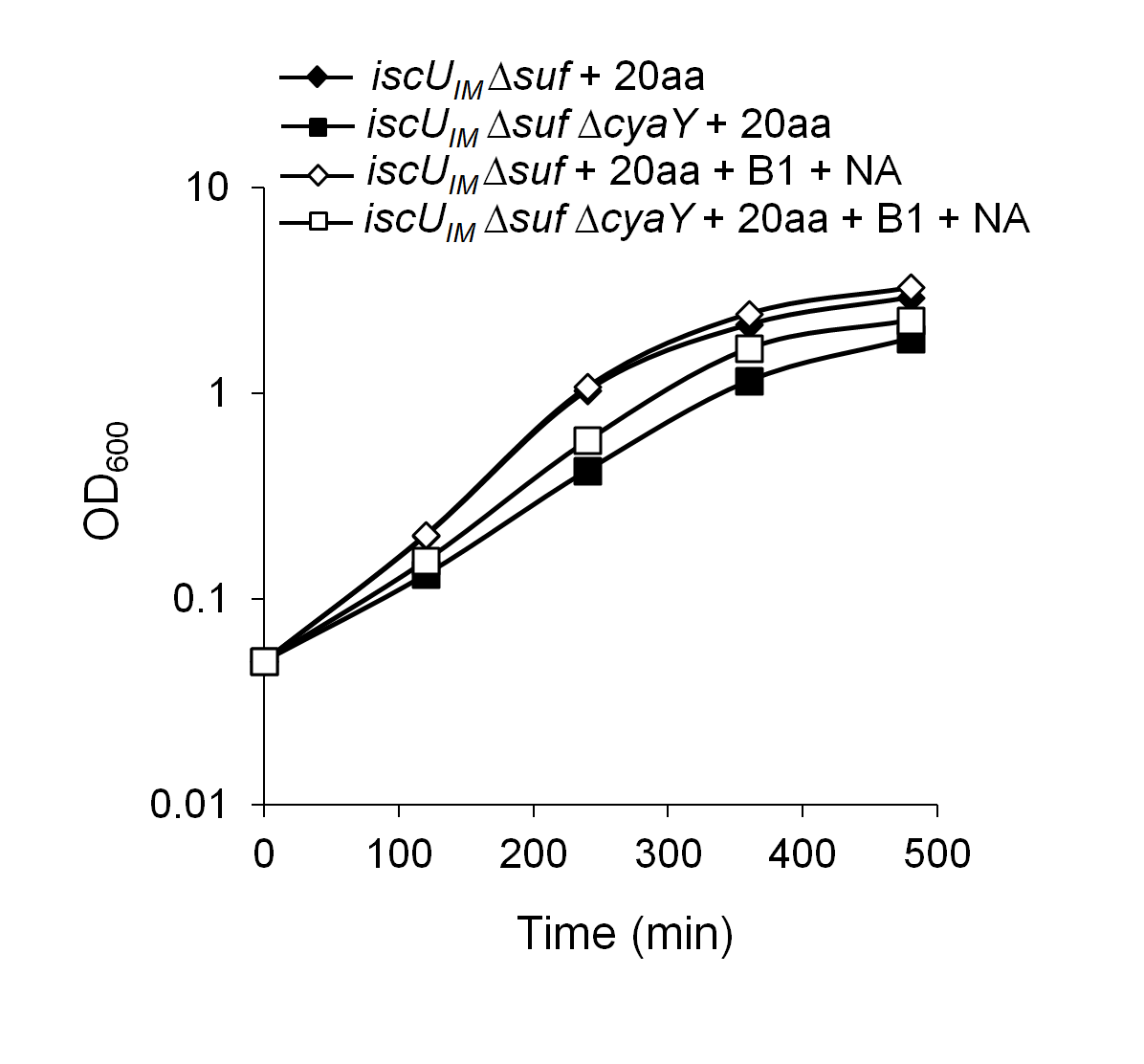

Supplement: S1 Fig — Growth of the iscU IM Δ suf (BR763) (diamonds) and iscU IM Δsuf ΔcyaY (BR767) (squares) strains in glucose M9 minimal medium supplemented with all amino acids and complemented with (white symbols) or without (black symbols) thiamine (B1) and nicotinic acid (NA). Growth was monitored at 600 nm. The experiment was repeated at least three times. One representative experiment is shown. (TIF) [file pgen.1005134.s001.tif]

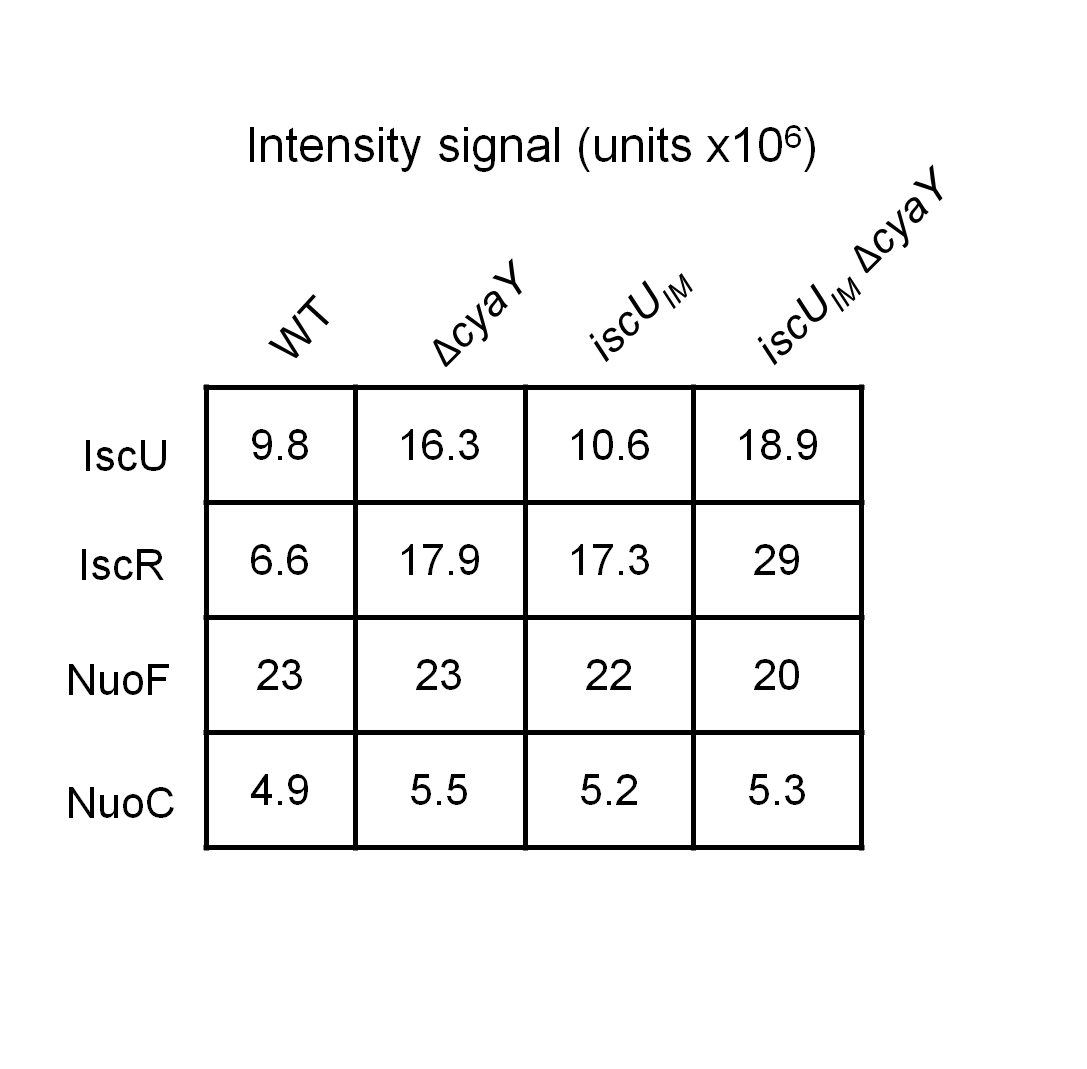

Supplement: S2 Fig — Quantification of western blots analysis of results shown in Fig 4 was performed using ImageQuantTL software. (TIF) [file pgen.1005134.s002.tif]

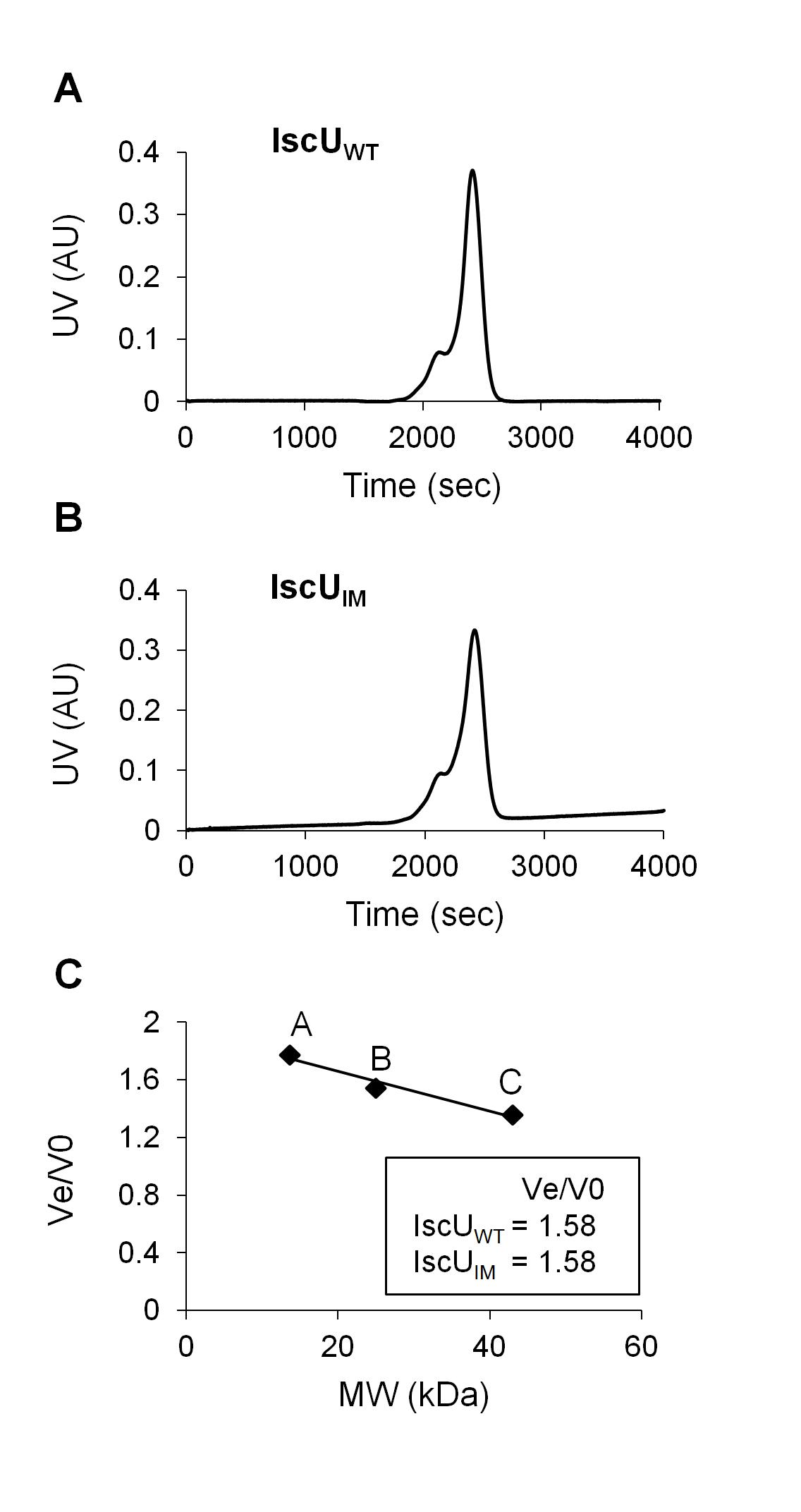

Supplement: S3 Fig — Comparison of the elution profiles between IscUWT (A) and IscUIM (B). For each protein, a gel filtration was performed on a Superdex 75 10/300 GL equilibrated with buffer A (0.1 M Tris-HCl pH 8, 50 mM NaCl). (C) Oligomerization state of IscUWT and IscUIM was determined from calibration curve using ribonuclease A (A; 13.7 kDa), chymotrypsinogen A (B; 25 kDa) and ovalbumin (C, 43 kDa) as molecular standards. Values of the elution volume (Ve)/ void volume (V0) are given for IscUWT and IscUIM. (TIF) [file pgen.1005134.s003.tif]

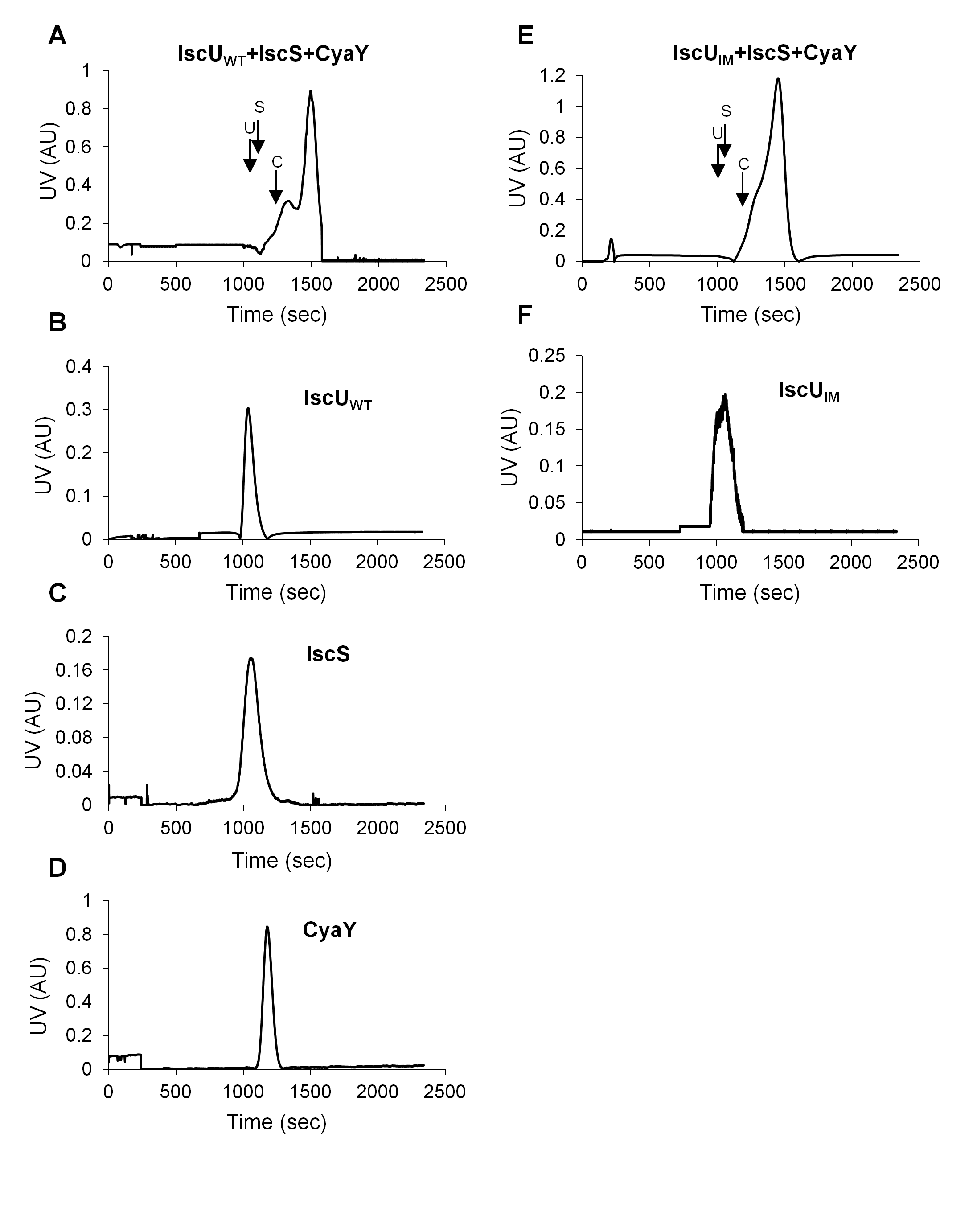

Supplement: S4 Fig — For each profile obtained from the mixtures of IscUWT/IscS/CyaY (A) and IscUIM /IscS/CyaY (E), the black arrows indicate the elution for each single protein (U: IscUWT/IM; S: IscS; C: CyaY) whose chromatographic profiles are shown below: IscUWT (B), IscS (C), CyaY (D), and IscUIM (F). Equilibration buffer of QFF column is 50 mM Tris-HCl pH 8 and elution was performed with a gradient of 50 mM Tris-HCl, pH 8, 1M NaCl. Flow rate: 1 mL/min. (TIF) [file pgen.1005134.s004.tif]

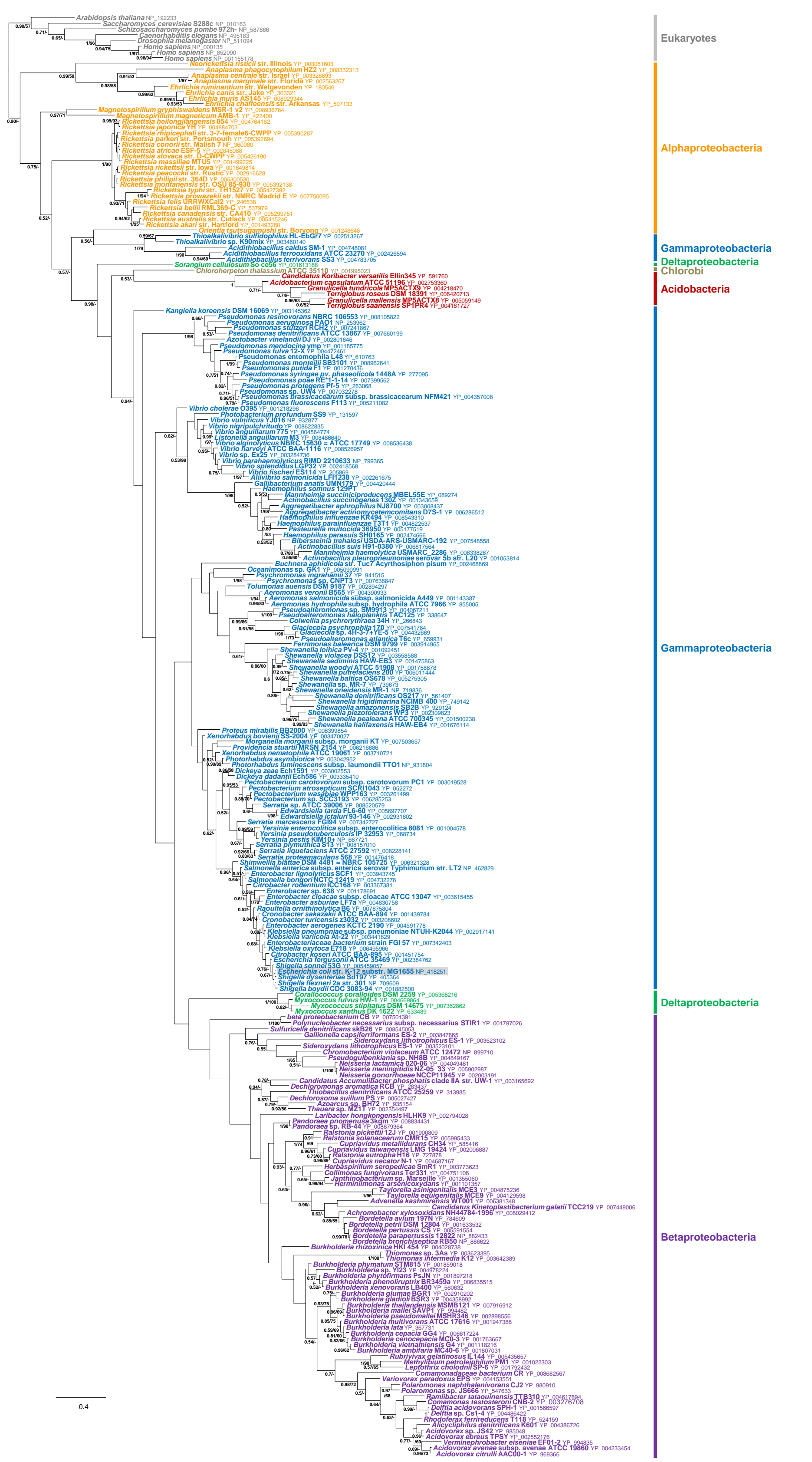

0.4

Supplement: S5 Fig — Unrooted Bayesian phylogenetic trees of CyaY (251 sequences, 70 positions). Numbers at nodes indicate posterior probabilities (PP) computed by MrBayes and bootstrap values (BV) computed by PhyML. Only PP and BV above 0.5 and 50% are shown. The scale bars represent the average number of substitutions per site. In the phylogenetic tree each prokaryotic phylum is highlighted in different colors: Alphaproteobacteria (orange), Gammaproteobacteria (blue), Deltaproteobacteria (green), Chlorobi (grey), Acidobacteria (red), Betaproteobacteria (purple). This color code is the same as the one used in the Fig 6. (PDF) [file pgen.1005134.s005.pdf]

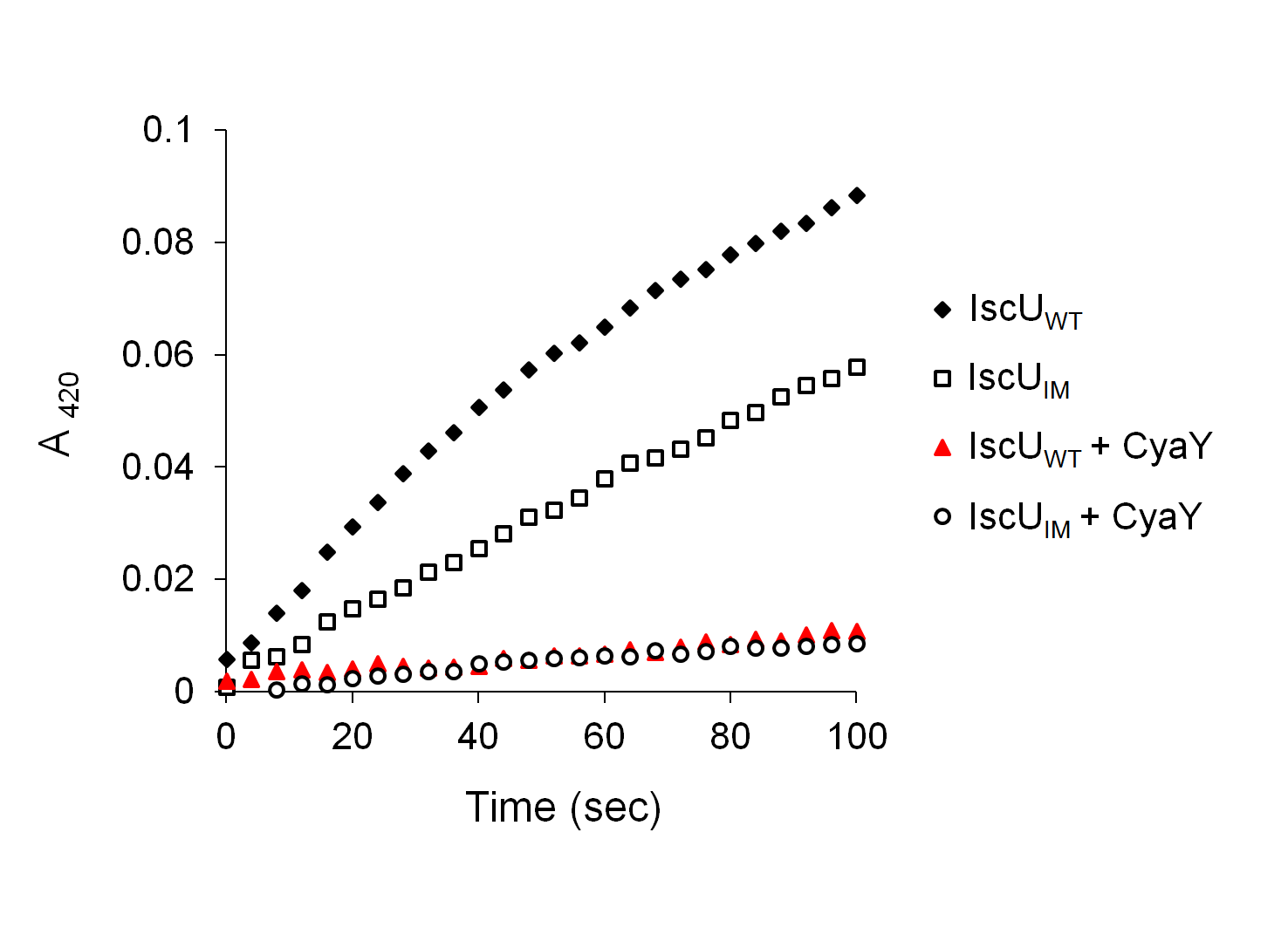

Supplement: S7 Fig — Comparison of the kinetics of enzymatic Fe-S cluster formation on IscUWT (black diamonds; red triangles) and IscUIM (white circles; white squares) with (red triangles; white circles) or without (black diamonds; white squares) CyaY. Experiment was carried out using 25 μM IscUWT or IscUIM, 25 μM IscS, 25 μM CyaY,100 μM Fe(SO4)2(NH4)2, 250 μM L-cysteine, 2 mM DTT. Fe-S cluster formation was followed by absorbance at 420 nm. The experiment was repeated at least three times. One representative experiment is shown. (TIF) [file pgen.1005134.s007.tif]

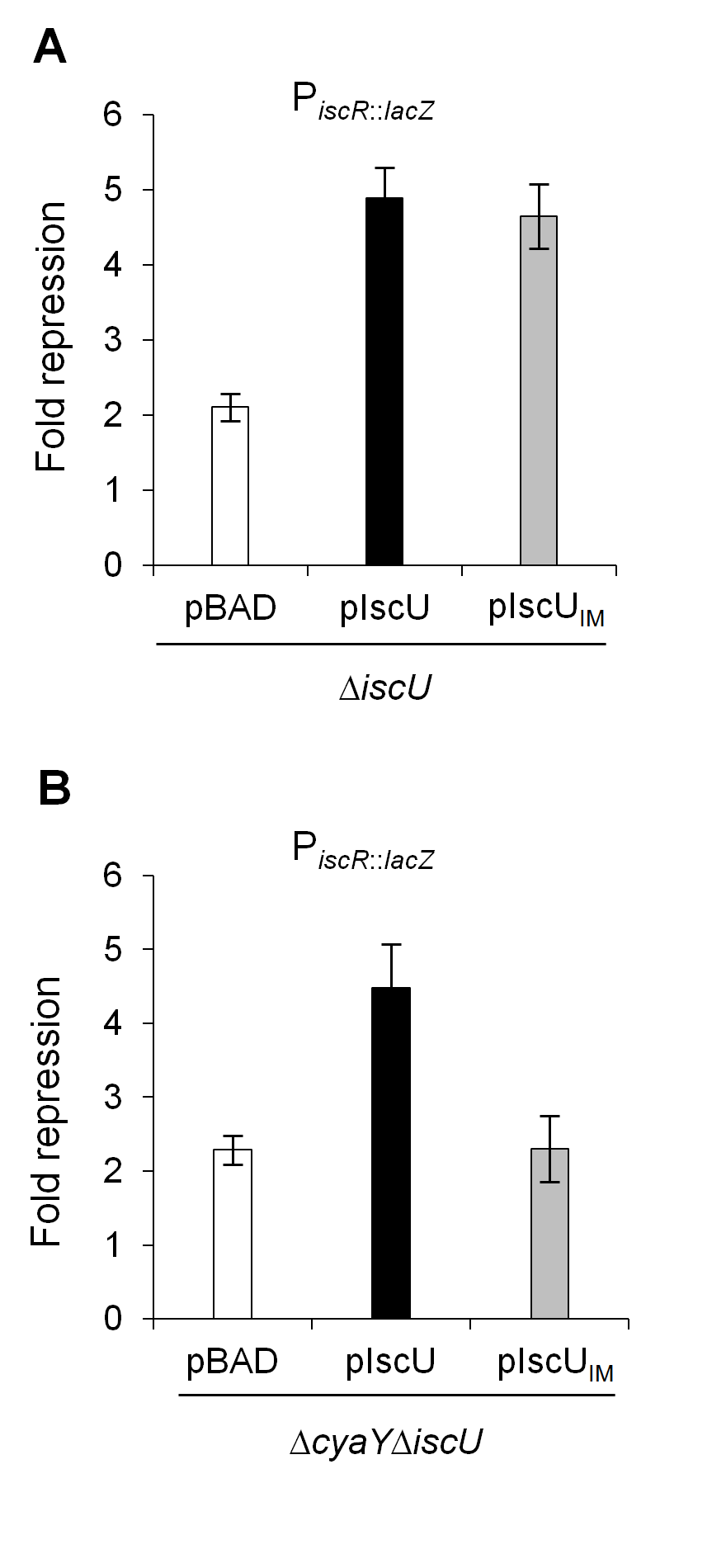

Supplement: S8 Fig — Repression of the IscR-regulated gene (iscR::lacZ) in the ΔiscU (BR667) mutant (A) and the ΔcyaY ΔiscU (BR668) mutant (B) transformed with pBAD (empty vector) (white bars), pIscU (black bars) or pIscUIM (grey bars) plasmids. Cultures were grown in LB medium supplemented with ampicillin and arabinose. The amount of IscR-dependent repression (fold repression) was determined by dividing the β-galactosidase activity present in the strain lacking IscR (DV915) by the β-galactosidase activity measured for each strain. Error bars represent the standard error from three independent experiments. (TIF) [file pgen.1005134.s008.tif]
