# Supplementary material for: Turning Escherichia coli into a Frataxin-Dependent Organism
Source: PLoS Genet. 2015 May 21;11(5):e1005134. doi: 10.1371/journal.pgen.1005134 (PMC4440780; doi:10.1371/journal.pgen.1005134)
Supplement: S6 Fig — IscU homologs were aligned using MAFFT v7.045b. The 99LPPVK103 motif and the residue at position 108 are indicated at the top of the alignment. Species having Methionine at position 108 are highlighted in yellow. (PDF) [file pgen.1005134.s006.pdf]

```
Alignment: C:\Users\ragrebi\Downloads\IscU_fin_seaview.fasta
Seaview [blocks=10 fontsize=9 A4] on Wed Feb 04 18:12:48 2015

                                      1
243233_Mcapsulatus@YP_112782          ---------- ---------- ---------- ---------- ----------
458817_Shalifaxensis@YP_001673798     ---------- ---------- ---------- ---------- ----------
398579_Spealeana@YP_001501349         ---------- ---------- ---------- ---------- ----------
557598_Lhongkongensis@YP_002796852    ---------- ---------- ---------- ---------- ----------
521006_Ngonorrhoeae@YP_002001906      ---------- ---------- ---------- ---------- ----------
489653_Nlactamica@YP_004048810        ---------- ---------- ---------- ---------- ----------
935589_Nmeningitidis@YP_005903962     ---------- ---------- ---------- ---------- ----------
395495_Lcholodnii@YP_001790394        ---------- ---------- ---------- ---------- ----------
999541_Bgladioli@YP_004361365         ---------- ---------- ---------- ---------- ----------
983917_Rgelatinosus@YP_005439140      ---------- ---------- ---------- ---------- ----------
1416914_Ppnomenusa@YP_008837319       ---------- ---------- ---------- ---------- ----------
1380774_Psp@YP_008881415              ---------- ---------- ---------- ---------- ----------
1249661_Bthailandensis@YP_007917844   ---------- ---------- ---------- ---------- ----------
320388_Bmallei@YP_993528              ---------- ---------- ---------- ---------- ----------
536230_Bpseudomallei@YP_002897469     ---------- ---------- ---------- ---------- ----------
395019_Bmultivorans@YP_001579332      ---------- ---------- ---------- ---------- ----------
626418_Bglumae@YP_002912250           ---------- ---------- ---------- ---------- ----------
365044_Pnaphthalenivor@YP_981803      ---------- ---------- ---------- ---------- ----------
266265_Bxenovorans@YP_553871          ---------- ---------- ---------- ---------- ----------
269482_Bvietnamiensis@YP_001115172    ---------- ---------- ---------- ---------- ----------
583345_Mmobilis@YP_003049105          ---------- ---------- ---------- ---------- ----------
666681_Mversatilis@YP_003674921       ---------- ---------- ---------- ---------- ----------
391038_Bphymatum@YP_001857685         ---------- ---------- ---------- ---------- ----------
398577_Bambifaria@YP_001808732        ---------- ---------- ---------- ---------- ----------
269482_Bvietnamiensis@YP_001120041    ---------- ---------- ---------- ---------- ----------
406425_Bcenocepacia@YP_001765425      ---------- ---------- ---------- ---------- ----------
1009846_Bcepacia@YP_006615433         ---------- ---------- ---------- ---------- ----------
482957_Blata@YP_369669                ---------- ---------- ---------- ---------- ----------
748280_Psp@YP_004848592               ---------- ---------- ---------- ---------- ----------
1229205_Bphenoliruptrix@YP_006834626  ---------- ---------- ---------- ---------- ---------M
1163617_Sdenitrificans@YP_008546117   ---------- ---------- ---------- ---------- ----------
266265_Bxenovorans@YP_559453          ---------- ---------- ---------- ---------- ----------
398527_Bphytofirmans@YP_001896197     ---------- ---------- ---------- ---------- ----------
375286_Jsp@YP_001352943               ---------- ---------- ---------- ---------- ----------
204773_Harsenicoxydans@YP_001100494   ---------- ---------- ---------- ---------- ----------
580332_Slithotrophicus@YP_003524869   ---------- ---------- ---------- ---------- ----------
159087_Daromatica@YP_285167           ---------- ---------- ---------- ---------- ----------
882378_Brhizoxinica@YP_004029636      ---------- ---------- ---------- ---------- ----------
85643_Tsp@YP_002355840                ---------- ---------- ---------- ---------- ----------
757424_Hseropedicae@YP_003776230      ---------- ---------- ---------- ---------- ----------
522306_CAccumulibacter@YP_003167247   ---------- ---------- ---------- ---------- ----------
76114_Aaromaticum@YP_160687           ---------- ---------- ---------- ---------- ----------
381666_Reutropha@YP_725668            ---------- ---------- ---------- ---------- ----------
977880_Ctaiwanensis@YP_002005171      ---------- ---------- ---------- ---------- ----------
1042878_Cnecator@YP_004684939         ---------- ---------- ---------- ---------- ----------
266264_Cmetallidurans@YP_583181       ---------- ---------- ---------- ---------- ----------
395494_Gcapsiferriform@YP_003846450   ---------- ---------- ---------- ---------- ----------
859655_Rsolanacearum@YP_005996961     ---------- ---------- ---------- ---------- ----------
402626_Rpickettii@YP_001898467        ---------- ---------- ---------- ---------- ----------
543913_bproteobacteriu@YP_007502653   ---------- ---------- ---------- ---------- ----------
62928_Asp@YP_933519                   ---------- ---------- ---------- ---------- ----------
640081_Dsuillum@YP_005028573          ---------- ---------- ---------- ---------- ----------
452638_Pnecessarius@YP_001797351      ---------- ---------- ---------- ---------- ----------
292415_Tdenitrificans@YP_314923       ---------- ---------- ---------- ---------- ----------
265072_Mflagellatus@YP_544916         ---------- ---------- ---------- ---------- ----------
582744_Mglucosetrophus@YP_003051619   ---------- ---------- ---------- ---------- ----------
887061_Msp@YP_004040241               ---------- ---------- ---------- ---------- ----------
891398_CTremblaya@YP_004707015        ---------- ---------- ---------- ---------- ----------
426114_Tarsenitoxydans@YP_003623866   ---------- ---------- ---------- ----MDVSFA ASGERFEEDK
75379_Tintermedia@YP_003642782        ---------- ---------- ---------- ---------- ----------
937774_Tequigenitalis@YP_004130301    ---------- ---------- ---------- ---------- ----------
1008459_Tasinigenitalis@YP_004874474  ---------- ---------- ---------- ---------- ----------
634503_Eictaluri@YP_002934567         ---------- ---------- ---------- ---------- ----------
718251_Etarda@YP_005700147            ---------- ---------- ---------- ---------- ----------
259536_Parcticus@YP_264760            ---------- ---------- ---------- ---------- ----------
335284_Pcryohalolentis@YP_580918      ---------- ---------- ---------- ---------- ----------
571800_Psp@YP_008163576               ---------- ---------- ---------- ---------- ----------
420662_Mpetroleiphilum@YP_001021452   ---------- ---------- ---------- ---------- ----------
243365_Cviolaceum@NP_900763           ---------- ---------- ---------- ---------- ----------
1208919_CKinetoplastiba@YP_007459239  ---------- ---------- ---------- ---------- ----------
983545_Gsp@YP_004435367               ---------- ---------- ---------- ---------- ----------
342610_Patlantica@YP_660819           ---------- ---------- ---------- ---------- ----------
743299_Aferrivorans@YP_004783591      ---------- ---------- ---------- ---------- ----------
243159_Aferrooxidans@YP_002425162     ---------- ---------- ---------- ---------- ----------
1005048_Cfungivorans@YP_004753777     ---------- ---------- ---------- ---------- ----------
669502_CProfftella@YP_008343646       ---------- ---------- ---------- ---------- ----------
757424_Hseropedicae@YP_003776535      ---------- ---------- ---------- ---------- ----------
395495_Lcholodnii@YP_001790080        ---------- ---------- ---------- ---------- ----------
257310_Bbronchiseptica@NP_888820      ---------- ---------- ---------- ---------- ----------
990288_Acaldus@YP_004748177           ---------- ---------- ---------- ---------- ----------
990288_Acaldus@YP_004748110           ---------- ---------- ---------- ---------- ----------
983917_Rgelatinosus@YP_005436383      ---------- ---------- ---------- ---------- ----------
360910_Bavium@YP_786035               ---------- ---------- ---------- ---------- ----------
1036672_Akashmirensis@YP_006379415    ---------- ---------- ---------- ---------- ----------
```

```
340100_Bpetrii@YP_001631386          ---------- ---------- ---------- ---------- ----------
523791_Kkoreensis@YP_003146685       ---------- ---------- ---------- ---------- ----------
156889_Mmarinus@YP_866958            ---------- ---------- ---------- ---------- ----------
404589_Asp@YP_001377835              ---------- ---------- ---------- ---------- ----------
290397_Adehalogenans@YP_463806       ---------- ---------- ---------- ---------- ----------
447217_Asp@YP_002132996              ---------- ---------- ---------- ---------- ----------
455488_Adehalogenans@YP_002491042    ---------- ---------- ---------- ---------- ----------
378806_Saurantiaca@YP_003955403      ---------- ---------- ---------- ---------- ----------
246197_Mxanthus@YP_633158            ---------- ---------- ---------- ---------- ----------
483219_Mfulvus@YP_004669542          ---------- ---------- ---------- ---------- ----------
1144275_Ccoralloides@YP_005368444    ---------- ---------- ---------- ---------- ----------
1278073_Mstipitatus@YP_007362455     ---------- ---------- ---------- ---------- ----------
448385_Scellulosum@YP_001617979      ---------- ---------- ---------- ---------- ----------
399739_Pmendocina@YP_001188990       ---------- ---------- ---------- ---------- ----------
1294143_Pdenitrificans@YP_007659193  ---------- ---------- ---------- ---------- ----------
208964_Paeruginosa@NP_252502         ---------- ---------- ---------- ---------- ----------
322710_Avinelandii@YP_002801150      ---------- ---------- ---------- ---------- ----------
644801_Pstutzeri@YP_007239445        ---------- ---------- ---------- ---------- ----------
1207075_Psp@YP_007027941             ---------- ---------- ---------- ---------- ----------
1114970_Pfluorescens@YP_005206437    ---------- ---------- ---------- ---------- ----------
220664_Pprotegens@YP_262044          ---------- ---------- ---------- ---------- ----------
351746_Pputida@YP_001266219          ---------- ---------- ---------- ---------- ----------
1435058_Pmonteilii@YP_008958359      ---------- ---------- ---------- ---------- ----------
743720_Pfulva@YP_004475358           ---------- ---------- ---------- ---------- ----------
264730_Psyringae@YP_273568           ---------- ---------- ---------- ---------- ----------
384676_Pentomophila@YP_606728        ---------- ---------- ---------- ---------- ----------
1430440_Mgryphiswaldens@YP_008937260 ---------- ---------- ---------- ---------- ----------
749219_Mcatarrhalis@YP_003626770     ---------- ---------- ---------- ---------- ----------
62977_Asp@YP_046090                  ---------- ---------- ---------- ---------- ----------
436717_Aoleivorans@YP_003732380      ---------- ---------- ---------- ---------- ----------
400667_Abaumannii@YP_001084660       ---------- ---------- ---------- ---------- ----------
871585_Acalcoaceticus@YP_004995269   ---------- ---------- ---------- ---------- ----------
511062_Osp@YP_005091814              ---------- ---------- ---------- ---------- ----------
318167_Sfrigidimarina@YP_751106      ---------- ---------- ---------- ---------- ----------
326297_Samazonensis@YP_927171        ---------- ---------- ---------- ---------- ----------
318161_Sdenitrificans@YP_562467      ---------- ---------- ---------- ---------- ----------
167879_Cpsychrerythrae@YP_267876     ---------- ---------- ---------- ---------- ----------
637905_Sviolacea@YP_003556341        ---------- ---------- ---------- ---------- ----------
225849_Spiezotolerans@YP_002311055   ---------- ---------- ---------- ---------- ----------
323850_Sloihica@YP_001094441         ---------- ---------- ---------- ---------- ----------
425104_Ssediminis@YP_001474605       ---------- ---------- ---------- ---------- ----------
392500_Swoodyi@YP_001760156          ---------- ---------- ---------- ---------- ----------
60481_Ssp@YP_737869                  ---------- ---------- ---------- ---------- ----------
211586_Soneidensis@NP_717861         ---------- ---------- ---------- ---------- ----------
693973_Sbaltica@YP_005273706         ---------- ---------- ---------- ---------- ----------
399804_Sputrefaciens@YP_006009782    ---------- ---------- ---------- ---------- ----------
298386_Pprofundum@YP_128966          ---------- ---------- ---------- ---------- ----------
28173_Vnigripulchritu@YP_008621944   ---------- ---------- ---------- ---------- ----------
1219076_Valginolyticus@YP_008537102  ---------- ---------- ---------- ---------- ----------
150340_Vsp@YP_003286982              ---------- ---------- ---------- ---------- ----------
345073_Vcholerae@YP_001216234        ---------- ---------- ---------- ---------- ----------
223926_Vparahaemolytic@NP_796976     ---------- ---------- ---------- ---------- ----------
882102_Vanguillarum@YP_004566935     ---------- ---------- ---------- ---------- ----------
575788_Vsplendidus@YP_002416251      ---------- ---------- ---------- ---------- ----------
1129794_Gpsychrophila@YP_007545568   ---------- ---------- ---------- ---------- ----------
196600_Vvulnificus@NP_933549         ---------- ---------- ---------- ---------- ----------
903510_Vfurnissii@YP_004993913       ---------- ---------- ---------- ---------- ----------
338187_Vcampbellii@YP_001444276      ---------- ---------- ---------- ---------- ----------
338187_Vcampbellii@YP_008526292      ---------- ---------- ---------- ---------- ----------
314282_Psp@YP_007639631              ---------- ---------- ---------- ---------- ----------
357804_Pingrahamii@YP_942749         ---------- ---------- ---------- ---------- ----------
326442_Phaloplanktis@YP_341160       ---------- ---------- ---------- ---------- ----------
234831_Psp@YP_004067531              ---------- ---------- ---------- ---------- ----------
595494_Tauensis@YP_002893203         ---------- ---------- ---------- ---------- ----------
396588_Tsulfidiphilus@YP_002513559   ---------- ---------- ---------- ---------- ----------
396588_Tsulfidiphilus@YP_002514125   ---------- ---------- ---------- ---------- ----------
998088_Averonii@YP_004393031         ---------- ---------- ---------- ---------- ----------
380703_Ahydrophila@YP_856284         ---------- ---------- ---------- ---------- ----------
382245_Asalmonicida@YP_001142383     ---------- ---------- ---------- ---------- ----------
342108_Mmagneticum@YP_422390         ---------- ---------- ---------- ---------- ----------
1245471_Presinovorans@YP_008101472   ---------- ---------- ---------- ---------- ----------
365044_Pnaphthalenivor@YP_982518     ---------- ---------- ---------- ---------- ----------
296591_Psp@YP_549004                 ---------- ---------- ---------- ---------- ----------
232721_Asp@YP_986388                 ---------- ---------- ---------- ---------- ----------
535289_Aebreus@YP_002553097          ---------- ---------- ---------- ---------- ----------
365046_Rtataouinensis@YP_004618947   ---------- ---------- ---------- ---------- ----------
946483_CSymbiobacter@YP_008681130    ---------- ---------- ---------- ---------- ----------
312309_Vfischeri@YP_204001           ---------- ---------- ---------- ---------- ----------
595537_Vparadoxus@YP_004156043       ---------- ---------- ---------- ---------- ----------
316275_Asalmonicida@YP_002262235     ---------- ---------- ---------- ---------- ----------
391735_Veiseniae@YP_997138           ---------- ---------- ---------- ---------- ----------
397945_Acitrulli@YP_970793           ---------- ---------- ---------- ---------- ----------
643561_Aavenae@YP_004235248          ---------- ---------- ---------- ---------- ----------
398578_Dacidovorans@YP_001565014     ---------- ---------- ---------- ---------- ----------
742013_Dsp@YP_004488168              ---------- ---------- ---------- ---------- ----------
338969_Aferrireducens@YP_523433      ---------- ---------- ---------- ---------- ----------
596154_Adenitrificans@YP_004388278   ---------- ---------- ---------- ---------- ----------
688245_Ctestosteroni@YP_003278774    ---------- ---------- ---------- ---------- ----------
```

```
1266738_Pmirabilis@YP_008398502      ---------- ---------- ---------- ---------- ----------
1124991_Mmorganii@YP_007505117       ---------- ---------- ---------- ---------- ----------
406817_Xnematophila@YP_003713469     ---------- ---------- ---------- ---------- ----------
291112_Pasymbiotica@YP_003040200     ---------- ---------- ---------- ---------- ----------
243265_Pluminescens@NP_930506        ---------- ---------- ---------- ---------- ----------
406818_Xbovienii@YP_003468915        ---------- ---------- ---------- ---------- ----------
273123_Ypseudotubercul@YP_071365     ---------- ---------- ---------- ---------- ----------
393305_Yenterocolitica@YP_001005390  ---------- ---------- ---------- ---------- ----------
1346614_Sliquefaciens@YP_008231656   ---------- ---------- ---------- ---------- ----------
1348660_Splymuthica@YP_008160622     ---------- ---------- ---------- ---------- ----------
399741_Sproteamaculans@YP_001479850  ---------- ---------- ---------- ---------- ----------
1249634_Smarcescens@YP_007345780     ---------- ---------- ---------- ---------- ----------
187410_Ypestis@NP_668657             ---------- ---------- ---------- ---------- ----------
1157951_Pstuartii@YP_006215182       ---------- ---------- ---------- ---------- ----------
344609_Sboydii@YP_001881320          ---------- ---------- ---------- ---------- ----------
104623_Ssp@YP_008524031              ---------- ---------- ---------- ---------- ----------
561229_Dzeae@YP_003003456            ---------- ---------- ---------- ---------- ----------
590409_Ddadantii@YP_003334610        ---------- ---------- ---------- ---------- ----------
561230_Pcarotovorum@YP_003018589     ---------- ---------- ---------- ---------- ----------
561231_Pwasabiae@YP_003258666        ---------- ---------- ---------- ---------- ----------
1166016_Psp@YP_006282128             ---------- ---------- ---------- ---------- ----------
218491_Patrosepticum@YP_051325       ---------- ---------- ---------- ---------- ----------
99287_Senterica@NP_461477            ---------- ---------- ---------- ---------- ----------
218493_Sbongori@YP_004731144         ---------- ---------- ---------- ---------- ----------
290339_Csakazakii@YP_001436836       ---------- ---------- ---------- ---------- ----------
693216_Cturicensis@YP_003211492      ---------- ---------- ---------- ---------- ----------
399742_Esp@YP_001177741              ---------- ---------- ---------- ---------- ----------
640513_Easburiae@YP_004829762        ---------- ---------- ---------- ---------- ----------
693444_Ebacterium@YP_007339349       ---------- ---------- ---------- ---------- ----------
290338_Ckoseri@YP_001451852          ---------- ---------- ---------- ---------- ----------
484021_Kpneumoniae@YP_002920722      ---------- ---------- ---------- ---------- ----------
637910_Crodentium@YP_003366009       ---------- ---------- ---------- ---------- ----------
640131_Kvariicola@YP_003438138       ---------- ---------- ---------- ---------- ----------
1191061_Koxytoca@YP_006500071        ---------- ---------- ---------- ---------- ----------
1286170_Rornithinolytic@YP_007872306 ---------- ---------- ---------- ---------- ----------
511145_Ecoli@NP_417024               ---------- ---------- ---------- ---------- ----------
585054_Efergusonii@YP_002381825      ---------- ---------- ---------- ---------- ----------
198214_Sflexneri@NP_708368           ---------- ---------- ---------- ---------- ----------
216599_Ssonnei@YP_005457544          ---------- ---------- ---------- ---------- ----------
300267_Sdysenteriae@YP_404261        ---------- ---------- ---------- ---------- ----------
716541_Ecloacae@YP_003614361         ---------- ---------- ---------- ---------- ----------
701347_Elignolyticus@YP_003940771    ---------- ---------- ---------- ---------- ----------
630626_Sblattae@YP_006318650         ---------- ---------- ---------- ---------- ----------
1028307_Eaerogenes@YP_004590373      ---------- ---------- ---------- ---------- ----------
557723_Hparasuis@YP_002474707        ---------- ---------- ---------- ---------- ----------
1005058_Ganatis@YP_004420698         ---------- ---------- ---------- ---------- ----------
1075089_Pmultocida@YP_005176279      ---------- ---------- ---------- ---------- ----------
205914_Hsomnus@YP_718490             ---------- ---------- ---------- ---------- ----------
1171377_Btrehalosi@YP_007548422      ---------- ---------- ---------- ---------- ----------
233412_Hducreyi@NP_873560            ---------- ---------- ---------- ---------- ----------
416269_Apleuropneumoni@YP_001053631  ---------- ---------- ---------- ---------- ----------
696748_Asuis@YP_006816777            ---------- ---------- ---------- ---------- ----------
1366053_Mhaemolytica@YP_008338465    ---------- ---------- ---------- ---------- ----------
1334187_Hinfluenzae@YP_008544790     ---------- ---------- ---------- ---------M AFARIEKRKV
634176_Aaphrophilus@YP_003007650     ---------- ---------- ---------- ---------- ----------
694569_Aactinomycetemc@YP_006286355  ---------- ---------- ---------- ---------- ----------
862965_Hparainfluenzae@YP_004822800  ---------- ---------- ---------- ---------- ----------
221988_Msucciniciprodu@YP_088916     ---------- ---------- ---------- ---------- ----------
339671_Asuccinogenes@YP_001344170    ---------- ---------- ---------- ---------- ----------
550540_Fbalearica@YP_003914035       ---------- ---------- ---------- ---------- ----------
396595_Tsp@YP_003460082              ---------- ---------- ---------- ---------- ----------
413404_CRuthia@YP_903798             ---------- ---------- ---------- ---------- ----------
412965_CVesicomyosociu@YP_001219373  ---------- ---------- ---------- ---------- ----------
1208921_CKinetoplastiba@YP_007448562 ---------- ---------- ---------- ---------- ----------
561501_Baphidicola@YP_002468877      ---------- ---------- ---------- ---------- ----------
357244_Otsutsugamushi@YP_001248706   ---------- ---------- ---------- --------MN NDAKTVTGTR
1003201_Rtyphi@YP_005427548          ---------- ---------- ---------- ---------- ----------
1105111_CRickettsia@YP_005365544     ---------- ---------- ---------- ---------- ----------
272944_Rconorii@NP_360366            ---------- ---------- ---------- ---------- ----------
452659_Rrickettsii@YP_001650107      ---------- ---------- ---------- ---------- ----------
347255_Rafricae@YP_002845312         ---------- ---------- ---------- ---------- ----------
562019_Rpeacockii@YP_002916645       ---------- ---------- ---------- ---------- ----------
1105113_Rrhipicephali@YP_005390607   ---------- ---------- ---------- ---------- ----------
416276_Rmassiliae@YP_001499486       ---------- ---------- ---------- ---------- ---------M
1105108_Rparkeri@YP_005392983        ---------- ---------- ---------- ---------- ----------
1105114_Rmontanensis@YP_005391246    ---------- ---------- ---------- ---------- ----------
1032845_Rheilongjiangen@YP_004764467 ---------- ---------- ---------- ---------- ----------
652620_Rjaponica@YP_004884928        ---------- ---------- ---------- ---------- ----------
481009_Rphilipii@YP_005300823        ---------- ---------- ---------- ---------- ----------
1105109_Rslovaca@YP_005426472        ---------- ---------- ---------- ---------- ----------
1105107_Rcanadensis@YP_005299497     ---------- ---------- ---------- ---------- ----------
336407_Rbellii@YP_538116             ---------- ---------- ---------- ---------- ----------
315456_Rfelis@YP_246860              ---------- ---------- ---------- ---------- ----------
293614_Rakari@YP_001493572           ---------- ---------- ---------- ---------- ----------
1105110_Raustralis@YP_005414962      ---------- ---------- ---------- ---------- ----------
434131_Nristicii@YP_003081518        ---------- ---------- ---------- ---------- ----------
222891_Nsennetsu@YP_506192           ---------- ---------- ---------- ---------- ----------
330214_CNitrospira@YP_003799619      ---------- ---------- ---------- ---------- ----------
```

```
984262_Sgrandis@YP_005322201           ---------- ---------- ---------- ---------- ----------
100901_Wendosymbiont@YP_006555868      ---------- ---------- ---------- ---------- ----------
66084_Wsp@YP_002727379                 ---------- ---------- ---------- ---------- ----------
1423892_Emuris@YP_008929080            ---------- ---------- ---------- ---------- ----------
574556_Acentrale@YP_003328545          ---------- ---------- ---------- ---------- ----------
320483_Amarginale@YP_002563600         ---------- ---------- ---------- ---------- ----MGCIGG
1184253_Aphagocytophilu@YP_008332775   ---------- ---------- ---------- ---------- ----------
205920_Echaffeensis@YP_507440          ---------- ---------- ---------- ---------- ----------
254945_Eruminantium@YP_180281          ---------- ---------- ---------- ---------- ----------
254945_Eruminantium@YP_197308          ---------- ---------- ---------- ---------- ----------
269484_Ecanis@YP_303050                ---------- ---------- ---------- ---------- ----------
517418_Cthalassium@YP_001997584        ---------- ---------- ---------- ---------- ----------
485917_Pheparinus@YP_003091837         ---------- ---------- ---------- ---------- ----------
762903_Psaltans@YP_004273747           ---------- ---------- ---------- ---------- ----------
929556_Scanadensis@YP_006255074        ---------- ---------- ---------- ---------- ----------
760192_Hhydrossis@YP_004446110         ---------- ---------- ---------- ---------- ----------
1162668_Lferrooxidans@YP_005469656     ---------- ---------- ---------- ---------- ----------
1048260_Lferriphilum@YP_006765680      ---------- ---------- ---------- ---------- ----------
240015_Acapsulatum@YP_002755573        ---------- ---------- ---------- ---------- ----------
204669_CKoribacter@YP_589562           ---------- ---------- ---------- ---------- ----------
1198114_Gtundricola@YP_004219465       ---------- ---------- ---------- ---------- ----------
926566_Troseus@YP_006423699            ---------- ---------- ---------- ---------- ----------
401053_Tsaanensis@YP_004180969         ---------- ---------- ---------- ---------- ----------
682795_Gmallensis@YP_005055878         ---------- ---------- ---------- ---------- ----------
696127_CMidichloria@YP_004679282       ---------- ---------- ---------- ---------- ----------
871271_CZinderia@YP_003878048          ---------- ---------- ---------- ---------- ----------
657324_Bfibrisolvens@YP_007819038      ---------- ---------- ---------- ---------- ----------
768670_Cnitroreducens@YP_004051749     ---------- ---------- ---------- ---------- --------MA
717231_Fsinusarabici@YP_004603876      ---------- ---------- ---------- ---------- --------MA
639282_Ddesulfuricans@YP_003496772     ---------- ---------- ---------- ---------- --------MA
522772_Dacetiphilus@YP_003503972       ---------- ---------- ---------- ---------- --------MA
693746_Ovalericigenes@YP_004880314     ---------- ---------- ---------- ---------- ----------
657321_Rbromii@YP_007781362            ---------- ---------- ---------- ---------- ----------
657322_Fprausnitzii@YP_007799942       ---------- ---------- ---------- ---------- ---------M
213810_Rchampanellensi@YP_007828576    ---------- ---------- ---------- ---------- ----------
657319_[siraeum@YP_007775950           ---------- ---------- ---------- ---------- ----------
293826_Ametalliredigen@YP_001320267    ---------- ---------- ---------- ---------- ----------
350688_Aoremlandii@YP_001513205        ---------- ---------- ---------- ---------- ----------
546269_Falocis@YP_005054244            ---------- ---------- ---------- ---------- ----------
1511_[sticklandii@YP_003936518         ---------- ---------- ---------- ---------- ----------
699034_Pdifficile@YP_006198328         ---------- ---------- ---------- ---------- ----------
1128398_[acidurici@YP_006788189        ---------- ---------- ---------- ---------- ----------
572544_Ipolytropus@YP_003967871        ---------- ---------- ---------- ---------- ----------
469604_Fnucleatum@YP_008477745         ---------- ---------- ---------- ---------- ----------
580340_Tlienii@YP_004932350            ---------- ---------- ---------- ---------- ----------
891968_Amobile@YP_006444325            ---------- ---------- ---------- ---------- ----------
525903_Tacidaminovoran@YP_003316727    ---------- ---------- ---------- ---------- ----------
697281_Maustraliensis@YP_004463371     ---------- ---------- ---------- ---------- ----------
309799_Dthermophilum@YP_002251521      ---------- ---------- ---------- ---------- ----------
368407_Mmarisnigri@YP_001047821        ---------- ---------- ---------- ---------- ----------
903814_Elimosum@YP_003961665           ---------- ---------- ---------- ---------- ----------
931626_Awoodii@YP_005268656            ---------- ---------- ---------- ---------- ----------
410358_Mlabreanum@YP_001029708         ---------- ---------- ---------- ---------- ----------
679926_Mpetrolearius@YP_003895855      ---------- ---------- ---------- ---------- ----------
86416_Cpasteurianum@YP_007940944       ---------- ---------- ---------- ---------- ----------
929506_Cbotulinum@YP_004396224         ---------- ---------- ---------- ---------- ----------
386415_Cnovyi@YP_878355                ---------- ---------- ---------- ---------- ----------
748727_Cljungdahlii@YP_003779382       ---------- ---------- ---------- ---------- ----------
1341692_Cautoethanogenu@YP_008700753   ---------- ---------- ---------- ---------- ----------
747365_Tnarugense@YP_004437867         ---------- ---------- ---------- ---------- ----------
651822_Ffastidiosum@YP_007827223       ---------- ---------- ---------- ---------- ---------M
572547_Acolombiense@YP_003553035       ---------- ---------- ---------- ---------- ----------
212717_Ctetani@NP_781697               ---------- ---------- ---------- -----MGRLF KKSKGRGEII
431943_Ckluyveri@YP_001394709          ---------- ---------- ---------- ---------- ----------
573061_Ccellulovorans@YP_003843335     ---------- ---------- ---------- ---------- ----------
508767_Cbotulinum@YP_001920515         ---------- ---------- ---------- ---------- ----------
931276_Csaccharoperbut@YP_007454264    ---------- ---------- ---------- ---------- ----------
290402_Cbeijerinckii@YP_001308237      ---------- ---------- ---------- ---------- ----------
1345695_Csaccharobutyli@YP_008674363   ---------- ---------- ---------- ---------- ----------
269797_Mbarkeri@YP_305925              ---------- ---------- ---------- ---------- ----------
188937_Macetivorans@NP_617616          ---------- ---------- ---------- ---------- ----------
195103_Cperfringens@YP_696469          ---------- ---------- ---------- ---------- ----------
593750_Mformicica@YP_007249963         ---------- ---------- ---------- ---------- ----------
456442_Mboonei@YP_001405283            ---------- ---------- ---------- ---------- ----------
521011_Mpalustris@YP_002466865         ---------- ---------- ---------- ---------- ----------
323259_Mhungatei@YP_503638             ---------- ---------- ---------- ---------- ----------
547558_Mmahii@YP_003541558             ---------- ---------- ---------- ---------- ----------
644295_Mevestigatum@YP_003727500       ---------- ---------- ---------- ---------- ----------
351627_Csaccharolyticu@YP_001181022    ---------- ---------- ---------- ---------- ----------
521460_Cbescii@YP_002573580            ---------- ---------- ---------- ---------- ----------
632292_Chydrothermalis@YP_003992133    ---------- ---------- ---------- ---------- ----------
632348_Ckronotskyensis@YP_004023692    ---------- ---------- ---------- ---------- ----------
632335_Ckristjanssonii@YP_004026829    ---------- ---------- ---------- ---------- ----------
632516_Clactoaceticus@YP_004798736     ---------- ---------- ---------- ---------- ----------
608506_Cobsidiansis@YP_003840166       ---------- ---------- ---------- ---------- ----------
632518_Cowensensis@YP_004002802        ---------- ---------- ---------- ---------- ----------
1121335_[stercorarium@YP_007372524     ---------- ---------- ---------- ---------- ----------
1121335_[stercorarium@YP_007678972     ---------- ---------- ---------- ---------- ----------
```

```
755731_Csp@YP_005148252                  ---------- ---------- ---------- ---------- ----------
394503_Ccellulolyticum@YP_002506259      ---------- ---------- ---------- ---------- ----------
720554_[clariflavum@YP_005046370         ---------- ---------- ---------- ---------- ----------
203119_Rthermocellum@YP_001037149        ---------- ---------- ---------- ---------- ----------
642492_Clentocellum@YP_004309737         ---------- ---------- ---------- ---------- ----------
1384484_Aequolifaciens@YP_008663926      ---------- ---------- ---------- ---------- ---------M
515619_Erectale@YP_002936246             ---------- ---------- ---------- ---------- ----------
718255_Rintestinalis@YP_007780168        ---------- ---------- ---------- ---------- ----------
657315_Rintestinalis@YP_007830593        ---------- ---------- ---------- ---------- ----------
357809_Lphytofermentan@YP_001560003      ---------- ---------- ---------- ---------- ----------
610130_[saccharolyticu@YP_003823446      ---------- ---------- ---------- ---------- ----------
717608_[cf@YP_007849106                  ---------- ---------- ---------- ---------- ----------
245012_bproducing@YP_007810477           ---------- ---------- ---------- ---------- ----------
657313_Rtorques@YP_007786815             ---------- ---------- ---------- ---------- ----------
245018_bproducing@YP_007788847           ---------- ---------- ---------- ---------- ----------
585394_Rhominis@YP_004837975             ---------- ---------- ---------- ---------- ----------
1054217_Tarchaeon@YP_007688600           ---------- ---------- ---------- ---------- --------MY
657323_Rsp@YP_007784833                  ---------- ---------- ---------- ---------- ----------
657314_Robeum@YP_007804234               ---------- ---------- ---------- ---------- ----------
717962_Ccatus@YP_007770008               ---------- ---------- ---------- ---------- ----------
552811_Dlykanthroporep@YP_003758020      ---------- ---------- ---------- ---------- ------MNNG
246194_Chydrogenoforma@YP_361011         ---------- ---------- ---------- ---------- ----------
696281_Druminis@YP_004546667             ---------- ---------- ---------- ---------- ----------
868595_Dcarboxydivoran@YP_004497987      ---------- ---------- ---------- ---------- ----------
349161_Dreducens@YP_001112128            ---------- ---------- ---------- ---------- ----------
634498_Mruminantium@YP_003424309         ---------- ---------- ---------- ---------- ----------
406327_Mvannielii@YP_001322697           ---------- ---------- ---------- ---------- ----------
679901_Mzhilinae@YP_004616912            ---------- ---------- ---------- ---------- ----------
224719_Msp@YP_008075510                  ---------- ---------- ---------- ---------- ----------
339860_Mstadtmanae@YP_448034             ---------- ---------- ---------- ---------- ----------
1295009_CMethanomassili@YP_008071572     ---------- ---------- ---------- ---------- ----------
420247_Msmithii@YP_001272836             ---------- ---------- ---------- ---------- ----------
1041930_Mconradii@YP_005380864           ---------- ---------- ---------- ---------- ----------
304371_Mpaludicola@YP_003355464          ---------- ---------- ---------- ---------- ----------
1379702_Msp@YP_008915990                 ---------- ---------- ---------- ---------- ----------
351160_Marvoryzae@YP_686139              ---------- ---------- ---------- ---------- ----------
990316_Mconcilii@YP_004384883            ---------- ---------- ---------- ---------- -MAKGDRWMA
521011_Mpalustris@YP_002466708           ---------- ---------- ---------- ---------- -----MEDVP
1201294_Mbourgensis@YP_006544991         ---------- ---------- ---------- ---------- -------MTD
368407_Mmarisnigri@YP_001048051          ---------- ---------- ---------- ---------- -------MAD
635013_Tpotens@YP_003640662              ---------- ---------- ---------- ---------- ----------
457570_Nthermophilus@YP_001916489        ---------- ---------- ---------- ---------- ----------
574087_Aarabaticum@YP_003827335          ---------- ---------- ---------- ---------- ----------
748449_Hhalobius@YP_007315822            ---------- ---------- ---------- ---------- ----------
338963_Pcarbinolicus@YP_006717600        ---------- ---------- ---------- ---------- ----------
498761_Hmodesticaldum@YP_001680434       ---------- ---------- ---------- ---------- ----------
768704_Dmeridiei@YP_006620851            ---------- ---------- ---------- ---------- ----------
768706_Dorientis@YP_004969159            ---------- ---------- ---------- ---------- ----------
1147129_Dsp@YP_006909410                 ---------- ---------- ---------- ---------- ----------
138119_Dhafniense@YP_518658              ---------- ---------- ---------- ---------- ---------M
645991_Sglycolicus@YP_004266248          ---------- ---------- ---------- ---------- ----------
646529_Dacidiphilus@YP_006467680         ---------- ---------- ---------- ---------- ----------
272564_Dhafniense@YP_002460033           ---------- ---------- ---------- ---------- ----------
756499_Ddehalogenans@YP_006431093        ---------- ---------- ---------- ---------- ----------
871963_Ddichloroelimin@YP_007221311      ---------- ---------- ---------- ---------- ----------
767817_Dgibsoniae@YP_007945765           ---------- ---------- ---------- ---------- ----------
485916_Dacetoxidans@YP_003191710         ---------- ---------- ---------- ---------- ----------
555079_Toceani@YP_003825515              ---------- ---------- ---------- ---------- ----------
349307_Mthermophila@YP_843171            ---------- ---------- ---------- ---------- -------MIQ
760568_Dkuznetsovii@YP_004517387         ---------- ---------- ---------- ---------- ----------
373903_Horenii@YP_002508640              ---------- ---------- ---------- ---------- ----------
370438_Pthermopropioni@YP_001211605      ---------- ---------- ---------- ---------- ----------
264732_Mthermoacetica@YP_430496          ---------- ---------- ---------- ---------- ----------
96561_Doleovorans@YP_001528642           ---------- ---------- ---------- ---------- ----------
471821_uTermite@YP_001956213             ---------- ---------- ---------- ---------- ----------
429009_Adegensii@YP_003238425            ---------- ---------- ---------- ---------- ----------
706587_Dtiedjei@YP_006449617             ---------- ---------- ---------- ---------- ----------
589924_Fplacidus@YP_003435557            ---------- ---------- ---------- ---------- ----------
224325_Afulgidus@NP_069024               ---------- ---------- ---------- ---------- ----------
224325_Afulgidus@NP_069399               ---------- ---------- ---------- ---------- ----------
416591_Tlettingae@YP_001470347           ---------- ---------- ---------- ---------- ----------
688269_Tthermarum@YP_004660083           ---------- ---------- ---------- ---------- ----------
374847_CKorarchaeum@YP_001737319         ---------- ---------- ---------- ---------- -----MSTRV
583356_Iaggregans@YP_003860035           ---------- ---------- ---------- ---------- -----MSTRI
272947_Rprowazekii@NP_220862             ---------- ---------- ---------- ---------- ----------
1282356_Ppoae@YP_007398800               ---------- ---------- ---------- ---------- ----------
994484_Pbrassicacearum@YP_004352099      ---------- ---------- ---------- ---------- ----------
882944_Languillarum@YP_008488748         ---------- ---------- ---------- ---------- ----------
1167634_Axylosoxidans@YP_008032542       ---------- ---------- ---------- ---------- ----------
257311_Bparapertussis@NP_884287          ---------- ---------- ---------- ---------- ----------
1017264_Bpertussis@YP_005589900          ---------- ---------- ---------- ---------- ----------
284812_Spombe@NP_592967                  MSVFRRSVQC VGVLPSILAQ RSSLLARPAN LQFLKTNSSK FVPQVTANVS
559292_Scerevisiae@NP_014869             ---------- ---------- -------MFA RLANPAHFKP LTGSHITRAA
559292_Scerevisiae@NP_015190             ---------- ---------M LPVITRFARP ALMAIRPVNA MGVLRASSIT
9606_Hsapiens@NP_998760                  ---------- ---------M AAAGAFRLRR AASALLLRSP RLPARELSAP
9606_Hsapiens@NP_055116                  ---------- ---------- ---------- ---------- ----MVLIDM
3702_Athaliana@NP_193953                 ---------- ---------- -----MMLKQ AAKKALGLTS RQSTPWSVGI
3702_Athaliana@NP_192317                 ---------- ---------- ------MLRQ TTKRAFLGLA SQNPTPFPVV
```

```
3702_Athaliana@NP_186751        ---------- ---------- -------MMM LRQTSRKAYL GLQASPLGLG
7227_Dmelanogaster@NP_649840    ---------- ---------- --------MS LVRNSSRLLR SQLKRVQSVP
6239_Celegans@NP_502658         ---------- ---------- -------MSL QISSAAKTLL HKFALPAATS
```

```
                                          51
243233_Mcapsulatus@YP_112782              -MAYSDKVID HYENPRNVGS F-DKDED-GV GTGVVGAPAC GDVMKLQIKI
458817_Shalifaxensis@YP_001673798         -MAYSEKVID HYENPRNVGS F-DKNDP-SV VTGMVGAPAC GDVMKLQLRI
398579_Speleana@YP_001501349              -MAYSEKVID HYENPRNVGS F-DKNDP-SV VTGMVGAPAC GDVMKLQLRI
557598_Lhongkongensis@YP_002796852        -MAYSEKVIE HYEHPRNVGS F-EKGDS-SV GTGMVGAPAC GDVMKLQIKV
521006_Ngonorrhoeae@YP_002001906          -MAYSDKVID HYENPRNVGT F-DKNDE-SV GTGMVGAPAC GDVMRLQIKV
489653_Nlactamica@YP_004048810            -MAYSDKVID HYENPRNVGT F-DKNDE-SV GTGMVGAPAC GDVMRLQIKV
935589_Nmeningitidis@YP_005903962         -MAYSDKVID HYENPRNVGT F-DKNDE-SV GTGMVGAPAC GDVMRLQIKV
395495_Lcholodnii@YP_001790394            -MAYSDKVIE HYENPRNVGG F-TAEDD-DV GTGMVGAPAC GDVMKLQIKV
999541_Bgladioli@YP_004361365             -MSYSNKVLD HYENPRNVGS F-SKDDD-AV GTGMVGAPAC GDVMKLQIRV
983917_Rgelatinosus@YP_005439140          -MAYSEKVID HYENPRNVGG F-AAGDD-TV ATGMVGAPAC GDVMKLQIKV
1416914_Ppnomenusa@YP_008837319           -MSYSKEVLD HYENPRNVGS F-EKGDD-AV GTGMVGAPAC GDVMKLQIRV
1380774_Psp@YP_008881415                  -MSYSKEVLD HYENPRNVGS F-EKGDD-AV GTGMVGAPAC GDVMKLQIRV
1249661_Bthailandensis@YP_007917844       -MSYSNKVLD HYENPRNVGS F-AKDDE-AV GTGMVGAPAC GDVMKLQIRV
320388_Bmallei@YP_993528                  -MSYSNKVLD HYENPRNVGS F-AKDDD-TV GTGMVGAPAC GDVMKLQIRV
536230_Bpseudomallei@YP_002897469         -MSYSNKVLD HYENPRNVGS F-AKDDD-TV GTGMVGAPAC GDVMKLQIRV
395019_Bmultivorans@YP_001579332          -MSYSNKVLD HYENPRNVGS F-AKDDD-AV GTGMVGAPAC GDVMKLQIRV
626418_Bglumae@YP_002912250               -MSYSNKVLD HYENPRNVGS F-SKGDD-AV GTGMVGAPAC GDVMKLQIRV
365044_Pnaphthalenivor@YP_981803          -MAYSDKVVD HYENPRNVGA F-EAGDD-SV GTGMVGAPAC GDVMRLQIRV
266265_Bxenovorans@YP_553871              -MAYSDKVID HYENPRNVGA F-DPDAA-NV GTGMVGAPAC GDVMRLQIRV
269482_Bvietnamiensis@YP_001115172        -MAYSDKVLD HYENPRNVGS F-DAGAD-DV GTGMVGAPAC GDVMRLQIRV
583345_Mmobilis@YP_003049105              -MAYSDKVLD HYENPRNVGS L-DKNDP-QV GTGMVGAPAC GDVMKLMIKV
666681_Mversatilis@YP_003674921           -MAYSDKVLD HYENPRNVGT L-DKNDP-NV GTGMVGAPAC GDVMKLMIKV
391038_Bphymatum@YP_001857685             -MAYSDKVLD HYENPRNVGS F-AKDDD-TV GTGMVGAPAC GDVMKLQIRV
398577_Bambifaria@YP_001808732            -MSYSNKVLD HYENPRNVGS F-AKDDD-AV GTGMVGAPAC GDVMKLQIRV
269482_Bvietnamiensis@YP_001120041        -MSYSNKVLD HYENPRNVGS F-AKDDD-AV GTGMVGAPAC GDVMKLQIRV
406425_Bcenocepacia@YP_001765425          -MSYSNKVLD HYENPRNVGS F-AKDDD-TV GTGMVGAPAC GDVMKLQIRV
1009846_Bcepacia@YP_006615433             -MSYSNKVLD HYENPRNVGS F-AKDDD-AV GTGMVGAPAC GDVMKLQIRV
482957_Blata@YP_369669                    -MSYSNKVLD HYENPRNVGS F-AKDDD-TV GTGMVGAPAC GDVMKLQIRV
748280_Psp@YP_004848592                   -MAYSEQVLD HYENPRNVGS F-DKGDD-SV GTGMVGAPAC GDVMKLQIKV
1229205_Bphenoliruptrix@YP_006834626      IMAYSDKVLD HYENPRNVGS F-SKDDD-AV GTGMVGAPAC GDVMKLQIRV
1163617_Sdenitrificans@YP_008546117       -MSYSTKVLD HYENPRNVGT F-GKDDA-GV ATGMVGAPAC GDVMKLQIKV
266265_Bxenovorans@YP_559453              -MAYSDKVLD HYENPRNVGS F-AKDDD-AV GTGMVGAPAC GDVMKLQIRV
398527_Bphytofirmans@YP_001896197         -MAYSDKVLD HYENPRNVGS F-AKDDD-AV GTGMVGAPAC GDVMKLQIRV
375286_Jsp@YP_001352943                   -MAYSDKVLD HYENPRNVGA F-DKGDE-TI GTGMVGAPAC GDVMKLQIKV
204773_Harsenicoxydans@YP_001100494       -MAYSDKVLD HYENPRNVGA F-DKGDE-TI GTGMVGAPAC GDVMKLQIKV
580332_Slithotrophicus@YP_003524869       -MAYSEKVLD HYEHPRNVGS L-DKDDA-YV GTGMVGAPAC GDVMKLQIKV
159087_Daromatica@YP_285167               -MSYSVKVID HYENPRNVGS F-AKEDD-GV GTGMVGAPAC GDVMKLQIKV
882378_Brhizoxinica@YP_004029636          -MAYSDKVLD HYENPRNVGS F-AKDDD-AV GTGMVGAPAC GDVMKLQIRV
85643_Tsp@YP_002355840                    -MAYSEKVLD HYENPRNVGS F-GKEEE-GV ATGMVGAPAC GDVMKLQIKV
757424_Hseropedicae@YP_003776230          -MAYSDKLMD HYENPRNVGS F-DKDDT-SV ATGMVGAPAC GDVMKLQIKV
522306_CAccumulibacter@YP_003167247       -MSYSVKVLD HYENPRNVGS F-GKEEE-GV ATGMVGAPAC GDVMKLQIKV
76114_Aaromaticum@YP_160687               -MAYSEKVLD HYENPRNVGA F-GKEDE-GV GTGMVGAPAC GDVMKLQIKV
381666_Reutropha@YP_725668                -MSYSTKVLD HYENPRNVGS F-DKNDD-AV GTGMVGAPAC GDVMKLQIKV
977880_Ctaiwanensis@YP_002005171          -MSYSTKVLD HYENPRNVGS F-DKNDD-AV GTGMVGAPAC GDVMKLQIKV
1042878_Cnecator@YP_004684939             -MSYSTKVLD HYENPRNVGS F-DKNDD-AV GTGMVGAPAC GDVMKLQIKV
266264_Cmetallidurans@YP_583181           -MSYSNKVLD HYENPRNVGS F-DKNDD-AV GTGMVGAPAC GDVMKLQIKV
395494_Gcapsiferriform@YP_003846450       -MAYSEKLLD HYENPRNVGS F-GKDDE-GL GTGMVGAPAC GDVMKLQIKV
859655_Rsolanacearum@YP_005996961         -MSYSNQVLD HYENPRNVGA F-EKGDD-TV GTGMVGAPAC GDVMKLQIKV
402626_Rpickettii@YP_001898467            -MSYSNQVLD HYENPRNVGS F-EKGDD-TV GTGMVGAPAC GDVMKLQIKV
543913_bproteobacteriu@YP_007502653       -MAYSDKVID HYENPRNVGS F-EKGDD-QV GTGMVGAPAC GDVMKLQIRV
62928_Asp@YP_933519                       -MAYSDKLLD HYENPRNVGS F-GKDDE-GV ATGMVGAPAC GDVMKLQIKV
640081_Dsuillum@YP_005028573              -MAYSVKVLD HYENPRNVGS F-TKEEEGGV GTGMVGAPAC GDVMKLQIKV
452638_Pnecessarius@YP_001797351          -MAYSEKVID HYENPRNVGS F-EKGDD-SV GTGMVGAPAC GDVMKLQIRV
292415_Tdenitrificans@YP_314923           -MSYSDKVLD HYENPRNVGA F-NKEDE-GV GTGMVGAPAC GDVMKLQIKV
265072_Mflagellatus@YP_544916             -MAYSDKVLD HYENPRNVGS L-DKNDP-SV GTGMVGAPAC GDVMKLQIKV
582744_Mglucosetrophus@YP_003051619       -MAYSDKVLD HYENPRNVGT L-DKNDP-SV GTGMVGAPAC GDVMKLQIKV
887061_Msp@YP_004040241                   -MAYSDKVLD HYENPRNVGT L-DKNDP-SV GTGMVGAPAC GDVMKLQIKV
891398_CTremblaya@YP_004707015            MGVYSKRVMR YCNDRRNVGS L-PEHES-DV GTGVAGSEAC GDVMRLQVRI
426114_Tarsenitoxydans@YP_003623866       IMAYSDKVVD HYENPRNVGS F-SKDDP-QV GTGMVGAPAC GDVMKLQIRV
75379_Tintermedia@YP_003642782            -MAYSDKVVD HYENPRNVGS F-AKDDP-QV GTGMVGAPAC GDVMKLQIRV
937774_Tequigenitalis@YP_004130301        -MAYSEKVLD HYENPRNVGS F-DKSDD-SV GTGMVGAPAC GDVMKLQIKV
1008459_Tasinigenitalis@YP_004874474      -MAYSEKVLD HYENPRNVGS F-DKSDE-SV GTGMVGAPAC GDVMKLQIKV
634503_Eictaluri@YP_002934567             -MAYSDKVID HYENPRNVGS F-DNSDP-NV GSGMVGAPAC GDVMKLQIKV
718251_Etarda@YP_005700147                -MAYSDKVID HYENPRNVGS F-DNSDP-NV GSGMVGAPAC GDVMKLQIKV
259536_Parcticus@YP_264760                -MAYSDQVID HYENPRNVGN L-DKNAK-NV GTGMVGAPAC GDVMRLQIQV
335284_Pcryohalolentis@YP_580918          -MAYSDQVID HYENPRNVGN L-DKNAK-NV GTGMVGAPAC GDVMRLQIQV
571800_Psp@YP_008163576                   -MAYSDQVID HYENPRNVGN L-DKNAK-NV GTGMVGAPAC GDVMRLQIQV
420662_Mpetroleiphilum@YP_001021452       -MAYSEKVVE HYENPRNVGS F-EKGDD-TV GTGMVGAPAC GDVMKLQIKV
243365_Cviolaceum@NP_900763               -MAYSEKVLD HYENPRNVGS F-DKGDD-SI GTGMVGAPAC GDVMKLQIKV
1208919_CKinetoplastiba@YP_007459239      -MAYSSKVLD HYENPRNVGS L-DKTDS-SV GTGMVGAPAC GDVMKLQIKV
983545_Gsp@YP_004435367                   -MAYSEKVID HYENPRNVGG F-DKSDP-SI ATGMVGAPAC GDVMKLQLKI
342610_Patlantica@YP_660819               -MAYSEKVID HYENPRNVGG F-DKNDP-SI ATGMVGAPAC GDVMKLQLKI
743299_Aferrivorans@YP_004783591          -MAYSEKVID HYENPRNVGA M-DKDDS-GV GTGMVGAPAC GDVMRLQIKV
243159_Aferrooxidans@YP_002425162         -MAYSEKVID HYEHPRNVGA L-DKDDS-GV GTGMVGAPAC GDVMRLQIRV
1005048_Cfungivorans@YP_004753777         -MSYSEKVLD HYENPRNVGA F-EKGDE-TV GTGMVGAPAC GDVMKLQIKV
669502_CProfftella@YP_008343646           -MSYSEKVLD HYENPRNVGV F-EKNDN-TV GTGMVGAPAC GDVMKLQIKV
757424_Hseropedicae@YP_003776535          -MSYSEKVLD HYENPRNVGA F-EKGDE-TV GTGMVGAPAC GDVMKLQIKV
395495_Lcholodnii@YP_001790080            -MAYSDKVID HYEHPRNVGA F-EKGDE-SV GTGMVGAPAC GDVMKLQIKV
257310_Bbronchiseptica@NP_888820          -MAYSSKVLD HYENPRNVGS F-DKGDD-SV GTGMVGAPAC GDVMRLQIKV
990288_Acaldus@YP_004748177               -MAYSEKVID HYENPRNVGS F-DKGDE-SV GTGMVGAPAC GDVMRLQIRV
990288_Acaldus@YP_004748110               -MAYSRKVLD HYEHPRNVGS F-DKNAA-EV GTGMVGAPAC GDVMRLQIKV
983917_Rgelatinosus@YP_005436383          -MAYSDKVVD HYENPRNVGA F-EKGDE-TV GTGMVGAPAC GDVMKLQIKV
360910_Bavium@YP_786035                   -MAYSDKVLD HYENPRNVGS F-EKGDD-SV GTGMVGAPAC GDVMKLQIKV
1036672_Akashmirensis@YP_006379415        -MAYSNKVLD HYENPRNVGS F-EKGDE-SI GTGMVGAPAC GDVMKLQIKV
340100_Bpetrii@YP_001631386               -MAYSSKVLD HYENPRNVGS F-DKGDE-SV GTGMVGAPAC GDVMKLQIKV
523791_Kkoreensis@YP_003146685            -MAYSEKVID HYENPRNVGS F-EKTDI-SI GTGMVGAPAC GDVMKLQIKV
156889_Mmarinus@YP_866958                 -MAYNEKVLD HYEKPRNVGS M-DKDAD-DV GTGMVGAPSC GDVMKLQIKV
```

```
404589_Asp@YP_001377835                 -MAYSDKLLD HYEHPRNVGS L-DKADP-DV GTGLVGAPAC GDVMKLQIKV
290397_Adehalogenans@YP_463806          -MAYSEKLID HYEHPRNVGS L-DKNDP-DV GTGLVGAPAC GDVMKLQIKV
447217_Asp@YP_002132996                 -MAYSEKLID HYEHPRNVGS L-DKNDP-DV GTGLVGAPAC GDVMKLQIKV
455488_Adehalogenans@YP_002491042       -MAYSEKLID HYEHPRNVGS L-DKNDP-DV GTGLVGAPAC GDVMKLQIKV
378806_Saurantiaca@YP_003955403         -MAYSDKVIE HYENPRNVGT L-DKADP-NV GTGLVGAPAC GDVMRLQLRI
246197_Mxanthus@YP_633158               -MAYSDKVIE HYENPRNVGT L-DKEDP-NV GTGLVGAPAC GDVMRLQLKI
483219_Mfulvus@YP_004669542             -MAYSDKVIE HYENPRNVGT L-DKEDP-NV GTGLVGAPAC GDVMRLQLKI
1144275_Ccoralloides@YP_005368444       -MAYSEKVIE HYENPRNVGT L-DKNDP-NV GTGLVGAPAC GDVMRLQLKI
1278073_Mstipitatus@YP_007362455        -MAYSDKVID HYENPRNVGT M-DKEDP-NV GTGLVGAPAC GDVMRLQLKI
448385_Scellulosum@YP_001617979         -MAYSDKVIE HYENPHNVGT L-DKNDE-RV GTGLVGAPAC GDVMRLQIKV
399739_Pmendocina@YP_001188990          -MAYSDKVID HYENPRNVGK L-NAEDP-DV GTGMVGAPAC GDVMRLQIKV
1294143_Pdenitrificans@YP_007659193     -MAYSDKVID HYENPRNVGK L-NAEDP-DV GTGMVGAPAC GDVMRLQIKV
208964_Paeruginosa@NP_252502            -MAYSEKVID HYENPRNVGK L-DAADP-NV GTGMVGAPAC GDVMRLQIKV
322710_Avinelandii@YP_002801150         -MAYSDKVID HYENPRNVGK L-DAQDP-DV GTGMVGAPAC GDVMRLQIKV
644801_Pstutzeri@YP_007239445           -MAYSDKVID HYENPRNVGK F-DAEDP-SI GTGMVGAPAC GDVMRLQIKV
1207075_Psp@YP_007027941                -MAYSEKVID HYENPRNVGK M-DAEDP-DV GTGMVGAPAC GDVMRLQIKV
1114970_Pfluorescens@YP_005206437       -MAYSEKVID HYENPRNVGK M-DAQDP-DV GTGMVGAPAC GDVMRLQIKV
220664_Pprotegens@YP_262044             -MAYSEKVID HYENPRNVGK M-NAEDP-DV GTGMVGAPAC GDVMRLQIKV
351746_Pputida@YP_001266219             -MAYSEKVID HYENPRNVGK M-NAEDP-DV GTGMVGAPAC GDVMRLQIKV
1435058_Pmonteilii@YP_008958359         -MAYSEKVID HYENPRNVGK M-NAEDP-DV GTGMVGAPAC GDVMRLQIKV
743720_Pfulva@YP_004475358              -MAYSEKVID HYENPRNVGK M-NAEDP-DV GTGMVGAPAC GDVMRLQIKV
264730_Psyringae@YP_273568              -MAYSEKVID HYENPRNVGK M-NAEDP-DV GTGMVGAPAC GDVMRLQIKV
384676_Pentomophila@YP_606728           -MAYSEKVID HYENPRNVGK M-NADDP-DV GTGMVGAPAC GDVMRLQIKV
1430440_Mgryphiswaldens@YP_008937260    -MAYSDKVVD HYEHPRNVGS M-DKDST-DV GTGLVGAPAC GDVMKLQIKV
749219_Mcatarrhalis@YP_003626770        -MAYSDKVID HYENPRNVGN L-DKAAK-NV GTGMVGAPAC GDVMRLQIQV
62977_Asp@YP_046090                     -MAYSEKVID HYENPRNVGI L-DKNAE-NV GTGMVGAPAC GDVMRLQIQV
436717_Aoleivorans@YP_003732380         -MAYSEKVID HYENPRNVGV L-DKNSE-NV GTGMVGAPAC GDVMRLQIQV
400667_Abaumannii@YP_001084660          ---------- ---------- ---------- ---MVGAPAC GDVMRLQIQV
871585_Acalcoaceticus@YP_004995269      -MAYSEKVID HYENPRNVGV L-DKNSE-NV GTGMVGAPAC GDVMRLQIQV
511062_Osp@YP_005091814                 -MAYSEKVID HYENPRNVGG F-DKNDP-NV ATGMVGAPAC GDVMKLQLKI
318167_Sfrigidimarina@YP_751106         -MAYSEKVID HYENPRNVGS F-DKNDP-SV VTGMVGAPAC GDVMKLQLKI
326297_Samazonensis@YP_927171           -MAYSEKVID HYENPRNVGS F-DKNDP-SV VTGMVGAPAC GDVMKLQLKI
318161_Sdenitrificans@YP_562467         -MAYSEKVID HYENPRNVGS F-DKNDP-SV VTGMVGAPAC GDVMKLQLKI
167879_Cpsychrerythrae@YP_267876        -MAYSEKVLD HYENPRNVGS M-DKNDP-SV ATGMVGAPAC GDVMKLQLKI
637905_Sviolacea@YP_003556341           -MAYSEKVID HYENPRNVGS F-DKNDP-SV VTGMVGAPAC GDVMKLQLKI
225849_Spiezotolerans@YP_002311055      -MAYSEKVID HYENPRNVGS F-DKNDS-SV VTGMVGAPAC GDVMKLQLRI
323850_Sloihica@YP_001094441            -MAYSEKVID HYENPRNVGS F-DKNDP-SV VTGMVGAPAC GDVMKLQLKI
425104_Ssediminis@YP_001474605          -MAYSEKVLE HYENPRNVGS F-DKNDP-SV VTGMVGAPAC GDVMKLQLKI
392500_Swoodyi@YP_001760156             -MAYSEKVLD HYENPRNVGS F-DKNDP-SV VTGMVGAPAC GDVMKLQLRI
60481_Ssp@YP_737869                     -MAYSEKVID HYENPRNVGS F-DKNDP-SV VTGMVGAPAC GDVMKLQLKI
211586_Soneidensis@NP_717861            -MAYSEKVID HYENPRNVGS F-DKNDP-SV VTGMVGAPAC GDVMKLQLKI
693973_Sbaltica@YP_005273706            -MAYSEKVID HYENPRNVGS F-DKNDP-SV VTGMVGAPAC GDVMKLQLKI
399804_Sputrefaciens@YP_006009782       -MAYSEKVID HYENPRNVGS F-DKNDP-SV VTGMVGAPAC GDVMKLQLKI
298386_Pprofundum@YP_128966             -MAYSEKVIE HYENPRNVGS F-DKNDK-NI GSGMVGAPAC GDVMKLQIKV
28173_Vnigripulchritu@YP_008621944      -MAYSEKVID HYENPRNVGS F-DKEDP-SV GSGMVGAPAC GDVMKLQIKV
1219076_Valginolyticus@YP_008537102     -MAYSEKVID HYENPRNVGS F-DKEDP-SV GSGMVGAPAC GDVMKLQIKV
150340_Vsp@YP_003286982                 -MAYSEKVID HYENPRNVGS F-DKEDP-SV GSGMVGAPAC GDVMKLQIKV
345073_Vcholerae@YP_001216234           -MAYSEKVID HYENPRNVGS F-DKEDP-SV GSGMVGAPAC GDVMRLQIKV
223926_Vparahaemolytic@NP_796976        -MAYSEKVID HYENPRNVGS F-DKEDP-SV GSGMVGAPAC GDVMKLQIKV
882102_Vanguillarum@YP_004566935        -MAYSEKVID HYENPRNVGS F-DKEDP-TI GSGMVGAPAC GDVMKLQIKV
575788_Vsplendidus@YP_002416251         -MAYSEKVID HYENPRNVGS F-DKEDP-SV GSGMVGAPAC GDVMKLQIKV
1129794_Gpsychrophila@YP_007545568      -MAYSEKVID HYENPRNVGG F-DKNDP-SI ATGMVGAPAC GDVMKLQLKI
196600_Vvulnificus@NP_933549            -MAYSEKVID HYENPRNVGS F-DKEDP-SV GSGMVGAPAC GDVMKLQIKV
903510_Vfurnissii@YP_004993913          -MAYSEKVID HYENPRNVGS F-DKEDP-SI GSGMVGAPAC GDVMRLQIKV
338187_Vcampbellii@YP_001444276         -MAYSEKVID HYENPRNVGS F-DKEDP-SV GSGMVGAPAC GDVMKLQIKV
338187_Vcampbellii@YP_008526292         -MAYSEKVID HYENPRNVGS F-DKEDP-SV GSGMVGAPAC GDVMKLQIKV
314282_Psp@YP_007639631                 -MAYSEKVID HYENPRNVGS FDDKDDK-SI ATGMVGAPAC GDVMKLQLKI
357804_Pingrahamii@YP_942749            -MAYSEKVID HYENPRNVGS F-DKDDL-SV ATGMVGAPAC GDVMKLQLKI
326442_Phaloplanktis@YP_341160          -MAYSDKVID HVENPRNVGV L-DKNDP-SV ATGMVGAPAC GDVMKLQIKV
234831_Psp@YP_004067531                 -MAYSDKVID HVENPRNVGA L-DKNDP-SV ATGMVGAPAC GDVMKLQIKV
595494_Tauensis@YP_002893203            -MAYSEKVID HYEHPRNVGS F-DKDDP-SV ATGMVGAPAC GDVMKLQLKV
396588_Tsulfidiphilus@YP_002513559      -MAYSDKVLD HYENPRNVGS L-DKADP-SV GTGMVGAPAC GDVMKLQIKV
396588_Tsulfidiphilus@YP_002514125      -MAYSDKVLD HYENPRNVGS L-DKADP-SV GTGMVGAPAC GDVMKLQIRV
998088_Averonii@YP_004393031            -MAYSEKVID HYENPRNVGG F-DKNDP-SV ATGMVGAPAC GDVMKLQLKI
380703_Ahydrophila@YP_856284            -MAYSEKVID HYENPRNVGG F-DKNDP-SI ATGMVGAPAC GDVMKLQLKI
382245_Asalmonicida@YP_001142383        -MAYSEKVID HYENPRNVGG F-DKNDP-NI ATGMVGAPAC GDVMKLQLKI
342108_Mmagneticum@YP_422390            -MAYSDKVID HYEHPRNVGA L-DKDDS-AV GTGLVGAPAC GDVMKLQIKV
1245471_Presinovorans@YP_008101472      -MAYSEKVID HYENPRNVGK L-DAQDP-NV GTGMVGAPAC GDVMRLQIKV
365044_Pnaphthalenivor@YP_982518        -MAYSEKVVD HYENPRNVGS F-EKGDE-TV GTGMVGAPAC GDVMKLQIKV
296591_Psp@YP_549004                    -MAYSEKVVD HYENPRNVGS F-EKGDD-SV GTGMVGAPAC GDVMKLQIKV
232721_Asp@YP_986388                    -MAYSDKVID HYENPRNVGS F-DKGDD-SV GTGMVGAPAC GDVMKLQIKV
535289_Aebreus@YP_002553097             -MAYSDKVID HYENPRNVGS F-DKGDD-SV GTGMVGAPAC GDVMKLQIKV
365046_Rtataouinensis@YP_004618947      -MAYSEKVVD HYENPRNVGS F-DKGDE-SV GTGMVGAPAC GDVMKLQIKV
946483_CSymbiobacter@YP_008681130       -MAYSEKVMD HYNNPRNVGS F-DKGDD-SV GTGMVGAPAC GDVMKLQIKV
312309_Vfischeri@YP_204001              -MAYSEKVID HYENPRNVGS F-EKDDP-SV GSGMVGAPAC GDVMKLQIKV
595537_Vparadoxus@YP_004156043          -MAYSSKVID HYENPRNVGS F-EKGDD-SV GTGMVGAPAC GDVMKLQIKV
316275_Asalmonicida@YP_002262235        -MAYSEKVID HYENPRNVGS F-EKDDP-SV GSGMVGAPAC GDVMKLQIKV
391735_Veiseniae@YP_997138              -MAYSEKVVD HYENPRNVGS F-DKGDD-SV GTGMVGAPAC GDVMKLQIKV
397945_Acitrulli@YP_970793              -MAYSDKVID HYENPRNVGS F-DKSDD-SV GTGMVGAPAC GDVMKLQIKV
643561_Aavenae@YP_004235248             -MAYSDKVID HYENPRNVGS F-DKSDD-SV GTGMVGAPAC GDVMKLQIKV
398578_Dacidovorans@YP_001565014        -MAYSDKVVD HYENPRNVGS F-DKNDE-SV GTGMVGAPAC GDVMKLQIKV
742013_Dsp@YP_004488168                 -MAYSDKVVD HYENPRNVGS F-DKNDE-SV GTGMVGAPAC GDVMKLQIKV
338969_Aferrireducens@YP_523433         -MAYSDKVVD HYENPRNVGS F-DKGDE-SV GTGMVGAPAC GDVMKLQIKV
596154_Adenitrificans@YP_004388278      -MAYSDKVID HYENPRNVGS F-DKGDD-TV GTGMVGAPAC GDVMKLQIKV
688245_Ctestosteroni@YP_003278774       -MAYSEKVVD HYENPRNVGS F-DKSDD-SV GTGMVGAPAC GDVMKLQIKV
1266738_Pmirabilis@YP_008398502         -MAYSDKVID HYENPRNVGS F-DNNDP-TV GSGMVGAPAC GDVMKLQIKV
1124991_Mmorganii@YP_007505117          -MAYSDKVID HYENPRNVGS F-DNEDP-TV GSGMVGAPAC GDVMKLQIKV
406817_Xnematophila@YP_003713469        -MAYSDKVID HYENPRNVGS F-DSEDP-SV GSGMVGAPAC GDVMRLQIKV
```

291112_Pasymbiotica@YP_003040200	-MAYSEKVID HYENPRNVGS F-DNEDP-TV GSGMVGAPAC GDVMKLQIKV
243265_Pluminescens@NP_930506	-MAYSEKVID HYENPRNVGS F-DSEDP-MV GSGMVGAPAC GDVMKLQIKV
406818_Xbovienii@YP_003468915	-MAYSDKVID HYENPRNVGS F-DSEDP-SV GSGMVGAPAC GDVMRLQIKV
273123_Ypseudotubercul@YP_071365	-MAYSEKVID HYENPRNVGS F-DSQDP-TI GSGMVGAPAC GDVMKLQIKV
393305_Yenterocolitica@YP_001005390	-MAYSEKVID HYENPRNVGS F-DNADP-TI GSGMVGAPAC GDVMKLQIKV
1346614_Sliquefaciens@YP_008231656	-MAYSEKVID HYENPRNVGS F-DNEDP-SV GSGMVGAPAC GDVMKLQIKV
1348660_Splymuthica@YP_008160622	-MAYSEKVID HYENPRNVGS F-DNEDP-TV GSGMVGAPAC GDVMKLQIKV
399741_Sproteamaculans@YP_001479850	-MAYSEKVID HYENPRNVGS F-DNEDP-SV GSGMVGAPAC GDVMKLQIKV
1249634_Smarcescens@YP_007345780	-MAYSEKVID HYENPRNVGS F-DNEDP-SV GSGMVGAPAC GDVMKLQIKV
187410_Ypestis@NP_668657	-MAYSEKVID HYENPRNVGS F-DSQDP-TI GSGMVGAPAC GDVMKLQIKV
1157951_Pstuartii@YP_006215182	-MAYSEKVID HYENPRNVGS F-DNNDP-SV GSGMVGAPAC GDVMKLQIKV
344609_Sboydii@YP_001881320	-MAYSEKVID HYENPRNVGS F-DNNDE-NV GSGMVGAPAC GDVMKLQIKV
104623_Ssp@YP_008524031	-MAYSEKVID HYENPRNVGS F-DNADP-NI GSGMVGAPAC GDVMKLQIKV
561229_Dzeae@YP_003003456	-MAYSEKVID HYENPRNVGS F-DSSDP-SI GSGMVGAPAC GDVMKLQIKV
590409_Ddadantii@YP_003334610	-MAYSEKVID HYENPRNVGS F-DSSDP-SI GSGMVGAPAC GDVMKLQIKV
561230_Pcarotovorum@YP_003018589	-MAYSEKVID HYENPRNVGS F-DNADP-SI GSGMVGAPAC GDVMKLQIKV
561231_Pwasabiae@YP_003258666	-MAYSEKVID HYENPRNVGS F-DNADP-SI GSGMVGAPAC GDVMKLQIKV
1166016_Psp@YP_006282128	-MAYSEKVID HYENPRNVGS F-DNADP-SI GSGMVGAPAC GDVMKLQIKV
218491_Patrosepticum@YP_051325	-MAYSEKVID HYENPRNVGS F-DNADP-SI GSGMVGAPAC GDVMKLQIKV
99287_Senterica@NP_461477	-MAYSEKVID HYENPRNVGS F-DNNDD-NV GSGMVGAPAC GDVMKLQIKV
218493_Sbongori@YP_004731144	-MAYSEKVID HYENPRNVGS F-DNNDD-NV GSGMVGAPAC GDVMKLQIKV
290339_Csakazakii@YP_001436836	-MAYSEKVID HYENPRNVGS F-DNNDD-NV GSGMVGAPAC GDVMKLQIKV
693216_Cturicensis@YP_003211492	-MAYSEKVID HYENPRNVGS F-DNNDD-NV GSGMVGAPAC GDVMKLQIKV
399742_Esp@YP_001177741	-MAYSEKVID HYENPRNVGS F-DNSDE-SV GSGMVGAPAC GDVMKLQIKV
640513_Easburiae@YP_004829762	-MAYSEKVID HYENPRNVGS F-DNSDE-SV GSGMVGAPAC GDVMKLQIKV
693444_Ebacterium@YP_007339349	-MAYSEKVID HYENPRNVGS F-DNSDE-SV GSGMVGAPAC GDVMKLQIKV
290338_Ckoseri@YP_001451852	-MAYSEKVID HYENPRNVGS F-DNSDE-NV GSGMVGAPAC GDVMKLQIKV
484021_Kpneumoniae@YP_002920722	-MAYSEKVID HYENPRNVGS F-DNSDE-NV GSGMVGAPAC GDVMKLQIKV
637910_Crodentium@YP_003366009	-MAYSEKVID HYENPRNVGS F-DNSDE-NV GSGMVGAPAC GDVMKLQIKV
640131_Kvariicola@YP_003438138	-MAYSEKVID HYENPRNVGS F-DNSDE-NV GSGMVGAPAC GDVMKLQIKV
1191061_Koxytoca@YP_006500071	-MAYSEKVID HYENPRNVGS F-DNSDE-NV GSGMVGAPAC GDVMKLQIKV
1286170_Rornithinolytic@YP_007872306	-MAYSEKVID HYENPRNVGS F-DNSDD-SV GSGMVGAPAC GDVMKLQIKV
511145_Ecoli@NP_417024	-MAYSEKVID HYENPRNVGS F-DNNDE-NV GSGMVGAPAC GDVMKLQIKV
585054_Efergusonii@YP_002381825	-MAYSEKVID HYENPRNVGS F-DNNDE-NV GSGMVGAPAC GDVMKLQIKV
198214_Sflexneri@NP_708368	-MAYSEKVID HYENPRNVGS F-DNNDE-NV GSGMVGAPAC GDVMKLQIKV
216599_Ssonnei@YP_005457544	-MAYSEKVID HYENPRNVGS F-DNNDE-NV GSGMVGAPAC GDVMKLQIKV
300267_Sdysenteriae@YP_404261	-MAYSEKVID HYENPRNVGS F-DNNDE-NV GSGMVGAPAC GDVMKLQIKV
716541_Ecloacae@YP_003614361	-MAYSEKVID HYENPRNVGS F-DNSDE-SV GSGMVGAPAC GDVMKLQIKV
701347_Elignolyticus@YP_003940771	-MAYSEKVID HYENPRNVGS F-DNSDE-NV GSGMVGAPAC GDVMKLQIKV
630626_Sblattae@YP_006318650	-MAYSEKVID HYENPRNVGS F-DNSDE-NV GSGMVGAPAC GDVMKLQIKV
1028307_Eaerogenes@YP_004590373	-MAYSEKVID HYENPRNVGS F-DNSDD-SV GSGMVGAPAC GDVMKLQIKV
557723_Hparasuis@YP_002474707	-MAYSDKVID HYENPRNVGS F-DKKAS-DI GTGMVGAPAC GDVMQLQIKV
1005058_Ganatis@YP_004420698	-MAYSEKVID HYENPRNVGS F-DKKDP-AV GSGMVGAPAC GDVMQLQIKV
1075089_Pmultocida@YP_005176279	-MAYSNKVID HYENPRNVGS L-DKKDA-NV GTGMVGAPAC GDVMQLQIKV
205914_Hsomnus@YP_718490	-MAYSEKLLD HYENPRNVGS F-DKKDP-TV GSGMVGAPAC GDVMQLQIKV
1171377_Btrehalosi@YP_007548422	---------- ---------- ---------- ---MVGAPAC GDILRLQIKV
233412_Hducreyi@NP_873560	-MAYSDKVID HYENPRNVGT F-DKEAT-DV GTGMVGAPAC GDILRLQIKV
416269_Apleuropneumoni@YP_001053631	-MAYSEKVID HYENPRNVGT F-DKEAA-DI GTGMVGAPAC GDILRLQIKV
696748_Asuis@YP_006816777	-MAYSEKVID HYENPRNVGT F-DKEAA-DI GTGMVGAPAC GDILRLQIKV
1366053_Mhaemolytica@YP_008338465	-MAYSEKVID HYENPRNVGT F-DKEAS-DT GTGMVGAPAC GDILRLQIKV
1334187_Hinfluenzae@YP_008544790	KMAYSEKVID HYENPRNVGS L-DKKDS-NV GTGMVGAPAC GDVMQLQIKV
634176_Aaphrophilus@YP_003007650	-MTYSNKVID HYENPRNVGS M-DKKDS-SV GTGMVGAPAC GDVMQLQIRV
694569_Aactinomycetemc@YP_006286355	---------- ---------- M-DRKDS-AV GTGMVGAPAC GDVMQLQIRV
862965_Hparainfluenzae@YP_004822800	-MAYSEKVID HYENPRNVGS M-DKKDN-SV GTGMVGAPAC GDVMQLQIKV
221988_Msucciniciprodu@YP_088916	-MAYSEKVID HYENPRNVGS F-DKKSS-DV GTGMVGAPAC GDVMQLQIKV
339671_Asuccinogenes@YP_001344170	-MAYSEKVID HYENPRNVGS F-DKKAS-DV GTGMVGAPAC GDVMQLQIKV
550540_Fbalearica@YP_003914035	-MAYSDKVID HYENPRNVGS F-DKNDP-NV ATGMVGAPAC GDVMKLQLKI
396595_Tsp@YP_003460082	-MAYSDKVLD HYENPRNVGS F-DKNDE-SV GTGMVGAPAC GDVMKLQIKV
413404_CRuthia@YP_903798	-MAYGEKVMD HYENPRNVGV L-DKDAK-NV GTGMVGAPAC GDVMRLQIQV
412965_CVesicomyosociu@YP_001219373	-MAYGKKVMD HYENPRNVGV L-DKNAK-NV GTGMVGAPAC GDVMRLQIKI
1208921_CKinetoplastiba@YP_007448562	-MAYSDKVLD HYENPRNVGS F-SKDDE-SV GTGMVGAPAC GDVMKLQIKV
561501_Baphidicola@YP_002468877	-MAYSKKVMD HYENPRNVGS F-SSTDL-NV GSGLVGAPAC GDVMKLQIKV
357244_Otsutsugamushi@YP_001248706	TAGYSSEVIK YYEDRSNVGS L-DETDK-SV GTGLVGAPAC GDVMKLQIKV
1003201_Rtyphi@YP_005427548	-MAYNKKVID HYENPRNVGS L-DKNKK-NV GTGLVGAPAC GDVMKLQIEV
1105111_CRickettsia@YP_005365544	-MAYSKKVID HYENPRNVGS L-DKENK-NV GTGLVGAPAC GDVMKLQIAV
272944_Rconorii@NP_360366	-MAYSKKVID HYENPRNVGS L-DKENK-NV GTGLVGAPAC GDVMKLQIAV
452659_Rrickettsii@YP_001650107	-MAYSKKVID HYENPRNVGS L-DKENK-NV GTGLVGAPAC GDVMKLQIAV
347255_Rafricae@YP_002845312	-MAYSKKVID HYENPRNVGS L-DKENK-NV GTGLVGAPAC GDVMKLQIAV
562019_Rpeacockii@YP_002916645	-MAYSKKVID HYENPRNVGS L-DKENK-NV GTGLVGAPAC GDVMKLQIAV
1105113_Rrhipicephali@YP_005390607	-MAYSKKVID HYENPRNVGS L-DKENK-NV GTGLVGAPAC GDVMKLQIAV
416276_Rmassiliae@YP_001499486	TMAYSKKVID HYENPRNVGS L-DKENK-NV GTGLVGAPAC GDVMKLQIAV
1105108_Rparkeri@YP_005392983	-MAYSKKVID HYENPRNVGS L-DKENK-NV GTGLVGAPAC GDVMKLQIAV
1105114_Rmontanensis@YP_005391246	-MAYSKKVID HYENPRNVGS L-DKENK-NV GTGLVGAPAC GDVMKLQIAV
1032845_Rheilongjiangen@YP_004764467	-MAYSKKVID HYANPRNVGS L-DKENK-NV GTGLVGAPAC GDVMKLQIAV
652620_Rjaponica@YP_004884928	-MAYSKKVID HYANPRNVGS L-DKENK-NV GTGLVGAPAC GDVMKLQIAV
481009_Rphilipii@YP_005300823	-MAYSKKVID HYENPRNVGS L-DKENK-NV GTGLVGAPAC GDVMKLQIAV
1105109_Rslovaca@YP_005426472	-MAYSKKVID HYENPRNVGS L-DKENK-NV GTGLVGAPAC GDVMKLQIAV
1105107_Rcanadensis@YP_005299497	-MAYSKKVID HYENPRNVGS L-NKESQ-NV GTGLVGAPAC GDVMKLQIEV
336407_Rbellii@YP_538116	-MAYSKKVID HYENPRNVGS L-DKEKK-NV GTGLVGAPAC GDVMKLQIEV
315456_Rfelis@YP_246860	-MAYSKKVID HYENPRNVGS L-DKEKK-NV GTGLVGAPAC GDVMKLQIEV
293614_Rakari@YP_001493572	-MAYSTKVID HYENPRNVGS L-DKEKK-NV GTGLVGAPAC GDVMKLQIEV
1105110_Raustralis@YP_005414962	-MAYSKKVID HYENPRNVGS L-DKEKK-NV GTGLVGAPAC GDVMKLQIEV
434131_Nristicii@YP_003081518	-MAYSDSVLN HYNNPRNVGT I-DKNKK-NV GTGLVGAPAC GDVMRLQIEV
222891_Nsennetsu@YP_506192	-MAYSDSVLN HYNNPRNVGT I-DKNKK-NV GTGLVGAPAC GDVMRLQIEV
330214_CNitrospira@YP_003799619	-MAYSEKVVD HFNNPRNMGS F-KKEED-GV GTGIVGAPEC GDVMKLQIKV
984262_Sgrandis@YP_005322201	-MAYSEKLLD HFKNPRNVGT L-DKNKN-TV GTGLVGAPEC GDVMRLQIEV
100901_Wendosymbiont@YP_006555868	-MSYNKKILD HYENPRNVGS L-DKNDP-NV GTGLVGAPSC GDVMKLQIKV
66084_Wsp@YP_002727379	-MSYNEKILD HYENPRNVGS L-DKNDP-NV GTGLVGAPSC GDVMKLQIKV

```
1423892_Emuris@YP_008929080                 -MSYSESLLE YYKNPKNVGT L-SKEDY-NV GTGLVGAPSC GDVMKLQIKV
574556_Acentrale@YP_003328545               -MSYSDAVLD RCKNPQNVGS L-PKDDL-NV GTGLVGAPSC GDVMKLQIKV
320483_Amarginale@YP_002563600              YMSYSDAVLD RCKNPQNVGS L-PKDDL-NV GTGLVGAPSC GDVMKLQIKV
1184253_Aphagocytophilu@YP_008332775        -MSYSDAVLN RCKAPQNVGS L-PKDDI-SV GTGLVGAPSC GDVMKLQIRV
205920_Echaffeensis@YP_507440               -MSYSESLLE HYKNPKNVGT L-PKEDY-NV GTGLVGAPSC GDVMKLQIKV
254945_Eruminantium@YP_180281               -MSYSESLLE HYKNPKNVGT L-PKEDY-NV GTGLVGAPSC GDVMKLQIKV
254945_Eruminantium@YP_197308               -MSYSESLLE HYKNPKNVGT L-PKEDY-NV GTGLVGAPSC GDVMKLQIKV
269484_Ecanis@YP_303050                     -MSYSEVLLE HYKNPKNVGT L-PKDDY-NV GTGLVGAPSC GDVMKLQIKV
517418_Cthalassium@YP_001997584             -MAYSDKVMD HYNNPRNVGS L-DQKDE-QV GTGVVGAPEC GDVMKLQIKV
485917_Pheparinus@YP_003091837              -MAYSEKVIE HYTNPRNVGT L-NKDSK-SV GTGLVGAPEC GDVMRLQIEV
762903_Psaltans@YP_004273747                -MAYSDKVID HYSNPRNVGT L-DKSKQ-NV GTGLVGAPEC GDVMRLQIEV
929556_Scanadensis@YP_006255074             -MAYSEKVID HYTHPRNVGT L-DKAKQ-NV GTGLVGAPEC GDVMRLQIEV
760192_Hhydrossis@YP_004446110              -MAYSDKVLE HFQNPRNVGT L-DKSKP-TV GTGLVGAPEC GDVMRLQIEV
1162668_Lferrooxidans@YP_005469656          -MAYSDKVID HYNNPRNMGS F-DKSDE-NV GTGIVGAPEC GDVMKLQLKI
1048260_Lferriphilum@YP_006765680           -MAYSDKVVD HYNNPRNMGS F-DKSEE-NV GTGIVGAPEC GDVMKLQLKI
240015_Acapsulatum@YP_002755573             -MAYSDKVID HYNNPRNVGQ L-DKSSE-AV GTGLVGAPEC GDVMRLQIRV
204669_CKoribacter@YP_589562                -MAYSEKVLD HYSNPRNVGS M-DKNSA-EV GTGLVGAPEC GDVMKLQIKV
1198114_Gtundricola@YP_004219465            -MAYSDKVVD HYSNPRNVGQ M-DKSLD-EV GTGLVGAPEC GDVMRLQIRV
926566_Troseus@YP_006423699                 -MAYSDKVVD HYENPRNVGT L-DKSSS-EV GTGLVGAPEC GDVMRLQIKV
401053_Tsaanensis@YP_004180969              -MAYSDKVVD HYENPRNVGT L-DKAST-EV GTGLVGAPEC GDVMRLQIKV
682795_Gmallensis@YP_005055878              -MAYSDKVVD HYENPRNVGT L-DKSST-EV GTGLVGAPEC GDVMRLQIKV
696127_CMidichloria@YP_004679282            -MAYSKKVID HYENPQNVGS F-AKDEE-NV GTGLVGAPSC GDVMKLQIKV
871271_CZinderia@YP_003878048               MTFYSKKLIK YCEKPKNIGS F-LKNNR-KI GTGITGSPSC GDVMKLQIKI
657324_Bfibrisolvens@YP_007819038           MALYSEKVMD HFRNPRNVGV I-ENAD---- GVGEVGNPVC GDIMKIYLKI
768670_Cnitroreducens@YP_004051749          KGPYSDKVMD HFMNPRNMGE I-EDAN---- GVGEVGNPAC GDVMKLFFKI
717231_Fsinusarabici@YP_004603876           KGPYSEKVME HFMNPRNMGE I-EDAS---- GVGEVGNPAC GDVMKLFLKI
639282_Ddesulfuricans@YP_003496772          KGPYSEKVMD HFMNPRNMGE I-EDAN---- AVGEVGNPAC GDVMKLYMKI
522772_Dacetiphilus@YP_003503972            KGPYSEKVMD HFMNPRNMGE I-EDAN---- GIGEVGNPAC GDVMKIFLKI
693746_Ovalericigenes@YP_004880314          -MLYSEKVMD HFQNPRNVGK M-DDAD---- GVGEVGNAKC GDIMRMYIKV
657321_Rbromii@YP_007781362                 -MAYSEKVMD HFANPRNVGE I-ENAD---- GIGEVGNSKC GDIMKMYIKV
657322_Fprausnitzii@YP_007799942            ASMYSAKVME HFANPHNVGE L-PDAN---- GVGEVGNPKC GDIMRMYLKI
213810_Rchampanellensi@YP_007828576         -MLYSEKVMD HFTNPRNVGE I-PDAD---- GVGEVGNAKC GDIMKMYIKV
657319_[siraeum@YP_007775950                -MIYSEKVMD HYANPRNVGE I-EDAN---- GVGEVGNAKC GDIMKMYLKI
293826_Ametalliredigen@YP_001320267         --MYSEKVMD HFTNPRNVGE I-ENAD---- GVGQVGNPKC GDIMKMYLKI
350688_Aoremlandii@YP_001513205             --MYSEKVMD HFMNPRNVGE I-EKAQ---- AVGEVGNAKC GDIMKMYLQI
546269_Falocis@YP_005054244                 -MLYTEKVMD HFNNPRNVGE I-PDAD---- GIGEVGNAKC GDIMRMYLKI
1511_[sticklandii@YP_003936518              --------MD HFTNPRNVGE I-EGAD---- GVGEVGNAKC GDIMKIYLKV
699034_Pdifficile@YP_006198328              -MQYSDKVME HFMNPRNMGE I-DNAS---- GVGEVGNPTC GDIMKIFLDI
1128398_[acidurici@YP_006788189             --MYSEKVME HFSNPRNVGE I-QDAD---- GIGEVGNAKC GDIMKMYLKI
572544_Ipolytropus@YP_003967871             -MQYSEKVMD HFMNPRNVGT I-ENPD---- GYGKVGSPSC GDVMEIFLKI
469604_Fnucleatum@YP_008477745              -MQYTEKVMQ HFMNPHNVGV I-ENPD---- GYGKVGNPSC GDIMEIFIKV
580340_Tlienii@YP_004932350                 --MYSEKVME LFMHPKNAGK I-EDAD---- GIGQVGNPQC GDIMKIYIKV
891968_Amobile@YP_006444325                 --MYTEKVME LFMNPKNVGR I-EDAD---- GIGEVGNPVC GDVMKIYLKI
525903_Tacidaminovoran@YP_003316727         -MAYSEKVVQ YFMNPVNVGE L-PDPD---- GVGEVGNPKC GDVMKIYLRI
697281_Maustraliensis@YP_004463371          --MYSQKVMD HFTNPRNVGE I-PDAD---- GVGEVGNPRC GDIMKIFIKV
309799_Dthermophilum@YP_002251521           --MYSEKVLE HFMNPRNVGE I-ENAD---- GVAQVGNPKC GDVMKMYLKI
368407_Mmarisnigri@YP_001047821             --MYTEKVIE EFTNPENVGE L-ADAD---- AVGEAGSPTC GDIMKIYLKV
903814_Elimosum@YP_003961665                MMEYTDKVME NFQNPKNVGE M-EDAN---- GIGQVGSPAC GDIMKMYLKI
931626_Awoodii@YP_005268656                 -MEYTDVVME NFTCPKHVGE L-EDAN---- GIGQVGSPAC GDIMKIFLKI
410358_Mlabreanum@YP_001029708              --MYSEKVMD HFTNPRNVGM I-EDPS---- GVGEEGNPSC GDIMKMYLKV
679926_Mpetrolearius@YP_003895855           --MYSDKVMD HFENPRNQGT I-EDAD---- GIGEVGNPQC GDIMKIYIKV
86416_Cpasteurianum@YP_007940944            -MMYSDKVMD HFRNPRNVGE I-ENAN---- GVGEVGNPQC GDIMKIYIKV
929506_Cbotulinum@YP_004396224              --MYSEKVMD HFRNPRNVGE I-ENPS---- GVGEVGNPQC GDIMKIYLEV
386415_Cnovyi@YP_878355                     --MYSEKVMD HFRNPRNVGE I-ENPS---- GVGEVGNPQC GDIMKIYLDV
748727_Cljungdahlii@YP_003779382            -MMYSEKVMD HFRHPRNVGE I-PDAN---- GVGEVGNPQC GDIMKIYIKV
1341692_Cautoethanogenu@YP_008700753        -MMYSEKVMD HFRHPRNVGE I-PDAN---- GVGEVGNPQC GDIMKIYIKV
747365_Tnarugense@YP_004437867              --MYSEKVMD HFRNPRNVGI I-SDAD---- GIGEVGNPRC GDIMKVYIKV
651822_Ffastidiosum@YP_007827223            ALDYSQKVMD HFTNPRNVGE L-KDAD---- GVGEAGNPRC GDIMKIYLKV
572547_Acolombiense@YP_003553035            -MLYSEIVMD HFQNPRNVGE I-KDAD---- GVGEAGNPRC GDIMKIYIKV
212717_Ctetani@NP_781697                    MMPYSDKVME HFRNPRNVGE I-SDAN---- GVGEVGNPKC GDIMKIYLKV
431943_Ckluyveri@YP_001394709               -MMYSEKVMD HFRNPRNVGE I-PDAN---- GVGEVGNPKC GDIMKMYIKV
573061_Ccellulovorans@YP_003843335          -MEYSEKVME HFTNPRNVGE I-ESAD---- GIGEVGNPKC GDIMKIYLKV
508767_Cbotulinum@YP_001920515              -MIYTDKVMD HFRNPRNVGE I-EDAN---- GVGEVGNAKC GDIMKIYLKV
931267_Csaccharoperbut@YP_007454264         -MIYSEKVMD HFQNPRNVGE I-ENAN---- GVGEVGNAKC GDIMKIYLKV
290402_Cbeijerinckii@YP_001308237           -MIYSEKVME HFQNPRNVGE I-VDAN---- GVGEVGNAKC GDIMKIYLKV
1345695_Csaccharobutyli@YP_008674363        -MIYSEKVME HFQNPRNVGE I-PDAN---- GVGEVGNAKC GDIMKIYLKV
269797_Mbarkeri@YP_305925                   -MDYSLKVMD HFSNPRNMGS I-ENSD---- GVGEVGNAKC GDIMKIYLKV
188937_Macetivorans@NP_617616               -MDYSAKVLD HFSNPRNMGS I-ANCD---- GVGEVGNAKC GDIMKIFLKV
195103_Cperfringens@YP_696469               -MIYSEKVMD HFKNPRNVGE I-KDAN---- GIGEVGNAKC GDIMRIYLKI
593750_Mformicica@YP_007249963              --MYSDKVMD HFKNPRNVGE I-ENPD---- GVGEVGNPVC GDIMNIYLKI
456442_Mboonei@YP_001405283                 --MYSETVMD HFKNPRNVGE I-ENPD---- GVGEVGNPVC GDIMKIFLKI
521011_Mpalustris@YP_002466865              --MYSEKVMD HFMNPRNVGE I-EDAD---- GIGEVGNPKC GDIMKIYLKI
323259_Mhungatei@YP_503638                  --MYSEKVMD HFMNPRNQGE I-PDAD---- GVGEVGNPTC GDIMRIFLTV
547558_Mmahii@YP_003541558                  -MMYSEKVME HFMNPHNVGE M-EDAD---- GVGEVGNPQC GDIMRIYLKI
644295_Mevestigatum@YP_003727500            MAMYSKKVMD HFMNPRNVGE M-ENPD---- GVGEVGNPKC GDIMRMYLKI
351627_Csaccharolyticu@YP_001181022         --MYSEKVLD HFMNPRNVGE I-EDAD---- GVAQVGNPKC GDIMKMYLKI
521460_Cbescii@YP_002573580                 --MYSEKVLD HFMNPRNVGE I-ENAD---- GVAQVGNPKC GDIMKMYLKI
632292_Chydrothermalis@YP_003992133         --MYSEKVLD HFMNPRNVGE I-ENAD---- GVAQVGNPKC GDIMKMYLKI
632348_Ckronotskyensis@YP_004023692         --MYSEKVLD HFMNPRNVGE I-ENAD---- GVAQVGNPKC GDIMKMYLKI
632335_Ckristjanssonii@YP_004026829         --MYSEKVLD HFMNPRNVGE I-ENAD---- GVAQVGNPKC GDIMKMYLKI
632516_Clactoaceticus@YP_004798736          --MYSEKVLD HFMNPRNVGE I-ENAD---- GVAQVGNPKC GDIMKMYLKI
608506_Cobsidiansis@YP_003840166            --MYSEKVLD HFMNPRNVGE I-ENAD---- GVAQVGNPKC GDIMKVYLKI
632518_Cowensensis@YP_004002802             --MYSEKVLD HFMNPRNVGE I-ENAD---- GVAQVGNPKC GDIMKMYLKI
1121335_[stercorarium@YP_007372524          -MMYSEKVMD HFMHPRNVGE I-EDAD---- GVGQVGNPKC GDIMKMYLKI
1121335_[stercorarium@YP_007678972          -MMYSEKVMD HFMHPRNVGE I-EDAD---- GVGQVGNPKC GDIMKMYLKI
755731_Csp@YP_005148252                     --MYSEKVMD HFSNPRNVGE I-EDAN---- GVGQVGNPKC GDIMKMYLKI
394503_Ccellulolyticum@YP_002506259         --MYSEKVMD HFSNPRNVGE I-EDAD---- GVGQVGNSKC GDIMKMYLKI
720554_[clariflavum@YP_005046370            --MYSEKVMD HFMNPRNVGE I-ENAD---- GVGEVGNAKC GDIMKIYLKI
```

203119_Rthermocellum@YP_001037149	-MMYSEKVMD	HFMNPRNVGE	I-ENAD----	GVGEVGNAKC	GDIMKMYLKI
642492_Clentocellum@YP_004309737	--MYSEKVMD	HFMNPRNVGE	I-ENAS----	GVGTVGNAKC	GDIMKVYLQI
1384484_Aequolifaciens@YP_008663926	AMNYSDKVME	HFTNPQNVGE	I-ENAS----	GCGTVGNAVC	GDIMRVYLDI
515619_Erectale@YP_002936246	-MAYSEKVMD	HFNHPRNVGE	M-ENPS----	GVGTVGNAKC	GDIMRMYLDI
718255_Rintestinalis@YP_007780168	--MYSEKVMD	HFNHPRNVGE	M-ENPS----	GVGTVGNAKC	GDIMRMYLDI
657315_Rintestinalis@YP_007830593	--MYSEKVMD	HFNHPRNVGE	M-ENPS----	GVGTVGNAKC	GDIMRMYLDI
357809_Lphytofermentan@YP_001560003	--MYTEKVMD	HFQHPRNVGE	L-ENAS----	GVGTVGNAKC	GDIMRMYLDI
610130_[saccharolyticu@YP_003823446	--MYTEKVMD	HFEHPRNVGE	I-DNPS----	GMGTVGNAKC	GDIMRIYLDI
717608_[cf@YP_007849106	--MYSEKVMD	HFQNPRNVGE	I-ENPS----	GMGTVGNAKC	GDIMRIYLDI
245012_bproducing@YP_007810477	--MYSEKVKD	HFQNPRNVGE	I-ENPS----	GMGTVGNAKC	GDIMRIYLDI
657313_Rtorques@YP_007786815	--MYTEKVMD	HFQHPRNVGE	I-ENAS----	GVGTVGNAKC	GDIMRIYLDI
245018_bproducing@YP_007788847	--MYSEKVMD	HFQHPRNVGE	I-ENAS----	GVGTVGNAKC	GDIMRIYLDI
585394_Rhominis@YP_004837975	--MYSEKVMD	HFQHPRNVGE	I-EDAS----	GVGTVGNAKC	GDIMRMYLDI
1054217_Tarchaeon@YP_007688600	DGVYSDKVVD	HFTNPRNVGE	I-ENAS----	GVGTVGNAKC	GDIMRIFLDI
657323_Rsp@YP_007784833	--MYSEKVMD	HFQHPRNVGE	I-ENAS----	GVGTVGNAKC	GDIMRIFLDI
657314_Robeum@YP_007804234	--MYSEKVMD	HFQHPRNVGE	I-EDAS----	GVGTVGNAKC	GDIMRMFLDI
717962_Ccatus@YP_007770008	--MYSEKVMD	HFQHPRNVGE	I-ENAS----	GVGTVGNAKC	GDIMRIYFDI
552811_Dlykanthroporep@YP_003758020	PASYSEQVIE	HVKNPRNIGE	L-ENPD----	GVGRVGNPVC	GDVMELYIKV
246194_Chydrogenoforma@YP_361011	-MMYSEKVMD	HFTNPRNVGE	I-PDAD----	GVGEVGNPSC	GDIMRIYIKV
696281_Druminis@YP_004546667	--MYSEKVMD	HFTSPRNVGE	I-ENAD----	GVGQVGNPSC	GDIMKISLKI
868595_Dcarboxydivoran@YP_004497987	--MYSEKVMD	HFTNPRNVGE	I-ENPD----	GVGQVGNPSC	GDIMKITLKI
349161_Dreducens@YP_001112128	--MYTEKVMD	HFTNPRNVGE	I-ENAD----	GIGQVGNPSC	GDIMKITLKV
634498_Mruminantium@YP_003424309	--MYSDKVMD	HFANPRNSGE	I-ENAS----	GEGTVGNPTC	GDLMTIYIDV
406327_Mvannielii@YP_001322697	--MYSEKVME	HFSNPKNAGE	I-KNPS----	GVGTVGNPSC	GDLMTIYIYV
679901_Mzhilinae@YP_004616912	--MYSKKVMD	HFKNPRNVGE	I-EDAD----	GVGEVGNPAC	GDMMTIYIKV
224719_Msp@YP_008075510	MEEYTDKVLD	HFAHPRNTGV	I-EDAS----	GEATVGNPTC	GDLMTFYIDV
339860_Mstadtmanae@YP_448034	-MQYSDKVVD	HYTNPRNTGV	I-KNPD----	GEGTVGNPVC	GDIMTIYIKV
1295009_CMethanomassili@YP_008071572	-MQYGPEVMN	NIANPRNSGV	M-KDAD----	GVGQVGNPEC	DDVTTVYIKV
420247_Msmithii@YP_001272836	--MYSEKVMD	HFANPRNSGE	M-ENPD----	GVGTVGNPTC	GDLMTIYIKV
1041930_Mconradii@YP_005380864	-MMYSPKVMQ	HFSNPQNVGE	I-SDAD----	GVGEVGNPVC	GDMMTIYIKV
304371_Mpaludicola@YP_003355464	-MMYSPKVMQ	HFSNPQNVGE	I-PDAD----	GVGEVGNPVC	GDLMTIYIKV
1379702_Msp@YP_008915990	--MYSEKVMD	HFSNPRNVGE	I-EDAS----	GVGTEGNPVC	GDLMTIYIKV
351160_Marvoryzae@YP_686139	--MYSPKVMT	HFSNPQNVGE	I-PDAD----	GVGTVGNPVC	GDMMTIYLKI
990316_Mconcilii@YP_004384883	QIGYNETVMD	HFMNPRNVGV	I-ENPD----	GYGKVGNPVC	GDLMEIFINV
521011_Mpalustris@YP_002466708	QIGYTEKVMD	HFMHPRNVGA	V-ADPD----	GVGQVGNPVC	GDLMEVSIKV
1201294_Mbourgensis@YP_006544991	QIGYSQKVME	HFMNPHNVGV	I-ENPD----	GYGKVGNPVC	GDLMEIFIRV
368407_Mmarisnigri@YP_001048051	QIGYSQKVME	HFMNPHNVGV	I-ENPD----	GYGKVGNPVC	GDIMEIFIRV
635013_Tpotens@YP_003640662	--MYNEKVMD	HFTNPRNVGE	I-EEAS----	GIGEVGNPTC	GDIMRISIKV
457570_Nthermophilus@YP_001916489	--MYNEKVMD	HFQNPRNVGE	V-ESPD----	GVGEVGNVTC	GDIMRISIKV
574087_Aarabaticum@YP_003827335	--MYSDKVMD	HFQNPRNVGE	I-EDAD----	GVGEVGNPTC	GDIMKMYITV
748449_Hhalobius@YP_007315822	MKDYSDEVMD	HFSNPRNVGE	I-EEAD----	GVGQVGNPQC	GDIMKMYIKV
338963_Pcarbinolicus@YP_006717600	--MYSDKVID	HFTNPRNVGE	I-ENAD----	GVGEKVSSSC	GDRMKVFLKV
498761_Hmodesticaldum@YP_001680434	--MYTDKVMD	HFMNPRNVGE	I-ENAS----	GVGEVGNASC	GDIMRIYLDV
768704_Dmeridiei@YP_006620851	--MYTEKVMD	HFTNPRNVGE	L-ENAD----	GVGEVGNAKC	GDIMRIYLDI
768706_Dorientis@YP_004969159	--MYTEKVMD	HFTNPRNVGE	I-DNAD----	GVGEVGNAKC	GDIMRIYLDI
1147129_Dsp@YP_006909410	--MYSEKVID	HFTNPRNVGE	I-ENAN----	GVGQVGNAKC	GDIMRISMVV
138119_Dhafniense@YP_518658	SDMYTEKVMD	HFMNPRNVGE	I-EDAN----	GVGEVGNAKC	GDIMRIYLDV
645991_Sglycolicus@YP_004266248	--MYSDKVLD	HFSNPRNVGE	L-ENAN----	GVGTVGNAKC	GDIMQIFLDI
646529_Dacidiphilus@YP_006467680	--MYTEKVMD	HFTNPRNVGE	I-ENAD----	GVGEVGNAKC	GDIMRIYLDV
272564_Dhafniense@YP_002460033	--MYTEKVMD	HFMNPRNVGE	I-EDAN----	GVGEVGNAKC	GDIMRIYLDV
756499_Ddehalogenans@YP_006431093	--MYTEKVMD	HFMNPRNVGE	I-ENPD----	GVGEVGNAKC	GDIMRIYLDV
871963_Ddichloroelimin@YP_007221311	--MYSEKVMD	HFTNPRNVGE	I-ENAD----	GVGEVGNAKC	GDIMRIYLDV
767817_Dgibsoniae@YP_007945765	--MYTDKVMD	HFENPRNVGE	I-SDAN----	GVGQVGNPTC	GDIMKIYLKI
485916_Dacetoxidans@YP_003191710	--MYSEKVMD	HFENPRNVGE	I-ENAN----	GIGQVGNPSC	GDIMKIYLVV
555079_Toceani@YP_003825515	--MYNEKVMD	HFTNPRNVGE	I-PDAD----	GIGEVGNPVC	GDMMKIYIKV
349307_Mthermophila@YP_843171	QTGYSKKVME	HFMNPRNVGV	I-DDPD----	GYGKVGNPVC	GDLMEIFIKV
760568_Dkuznetsovii@YP_004517387	--MYSEKVMD	HFTNPRNVGE	I-PDAD----	GVGQVGNPVC	GDIMKIYIKV
373903_Horenii@YP_002508640	--MYSEKVME	HFNNPRNVGE	I-KDAD----	AVGEVGNPVC	GDIIKLYLKI
370438_Pthermopropioni@YP_001211605	--MYSEKVIE	HFSNPRNVGE	I-PDAD----	GVGEIGNQVC	GDITKIFIKV
264732_Mthermoacetica@YP_430496	--MYSEKVMD	HFTNPRNVGE	I-ENAD----	GVGQVGNPVC	GDIMRLYIKV
96561_Doleovorans@YP_001528642	-MIYSKTVMD	HFRNPRNVGV	I-ENAA----	GVGEVGNPIC	GDMMTIYLDI
471821_uTermite@YP_001956213	MAFYSERVMD	HFANPRNVGE	I-KDAD----	GIGEVGNPVC	GDMMTFYIKV
429009_Adegensii@YP_003238425	--MYSAKVME	HFLHPRNVGV	I-EDAD----	GVGEVGNPAC	GDVMTFYIKV
706587_Dtiedjei@YP_006449617	--MYSDVVMD	HFKNPRNVGE	I-PDAD----	GVGEVGNPAC	GDIMAFYIKV
589924_Fplacidus@YP_003435557	--MYSEKVME	HFRNPRNVGV	I-EDAD----	GVGTVGNPVC	GDMMTIYIKV
224325_Afulgidus@NP_069024	--MYSDKVFD	HFQNPRNVGK	I-EDAD----	GVGTVGNPVC	GDLMTIYIKV
224325_Afulgidus@NP_069399	--MYSDKVFD	HFQNPRNVGK	I-EDAD----	GVGTVGNPVC	GDLMTIYIKV
416591_Tlettingae@YP_001470347	MLKYTELVLD	HFKNPRNLGK	I-ENAD----	AEATEGSIAC	GDMMTVYLKV
688269_Tthermarum@YP_004660083	MLKYTELVLD	HFKNPRNMGR	M-EDAD----	VTATEGSIAC	GDMMTVYLKI
374847_CKorarchaeum@YP_001737319	PLPYTPRVLE	LFREPKNLGR	I-DDAD----	AFAQAGSPAC	GDVISIYLRI
583356_Iaggregans@YP_003860035	PLPYSPKVLE	IFRNPKNLGP	L-ENAT----	VVESAGSPAC	GDMIKLYLRI
272947_Rprowazekii@NP_220862	-MAYSKKVID	HYENPRNVGS	L-DKKKK-NV	GTGLVGAPAC	GDVMKLQIEV
1282356_Ppoae@YP_007398800	-MAYSEKVID	HYENPRNVGK	M-NAEDP-DV	GTGMVGAPAC	GDVMRLQIKV
994484_Pbrassicacearum@YP_004352099	-MAYSEKVID	HYENPRNVGK	M-DAQDP-DV	GTGMVGAPAC	GDVMRLQIKV
882944_Languillarum@YP_008488748	-MAYSEKVID	HYENPRNVGS	F-DKEDP-TI	GSGMVGAPAC	GDVMKLQIKV
1167634_Axylosoxidans@YP_008032542	-MSYSTKVLD	HYENPRNVGS	F-DKSDD-SV	GTGMVGAPAC	GDVMKLQIKV
257311_Bparapertussis@NP_884287	-MAYSSKVLD	HYENPRNVGS	F-DKGDD-SV	GTGMVGAPAC	GDVMKLQIKV
1017264_Bpertussis@YP_005589900	-MAYSSKVLD	HYENPRNVGS	F-DKGDD-SV	GTGMVGAPAC	GDVMKLQIKV
284812_Spombe@NP_592967	RRMYHKNVLD	HYNNPRNVGT	L-PKGDP-DV	GIGLVGAPAC	GDVMRLAIRV
559292_Scerevisiae@NP_014869	KRLYHPKVID	HYTNPRNVGS	M-DKSLA-NV	GTGIVGAPAC	GDVIKLQIQV
559292_Scerevisiae@NP_015190	KRLYHPKVIE	HYTHPRNVGS	L-DKKLP-NV	GTGLVGAPAC	GDVMRLQIKV
9606_Hsapiens@NP_998760	ARLYHKKVVD	HYENPRNVGS	L-DKTSK-NV	GTGLVGAPAC	GDVMKLQIQV
9606_Hsapiens@NP_055116	SVDLSTQVVD	HYENPRNVGS	L-DKTSK-NV	GTGLVGAPAC	GDVMKLQIQV
3702_Athaliana@NP_193953	LRTYHENVID	HYDNPRNVGS	F-DKNDP-NV	GTGLVGAPAC	GDVMKLQIKV
3702_Athaliana@NP_192317	SRLYHPNVID	HYDNPRNVGS	F-DKNDP-NV	GTGLVGAPQC	GDVMKLQVKF
3702_Athaliana@NP_186751	RRLYHENVID	HFENPRNVGS	F-NRNDP-NV	GTGLVGAPAC	GDLMSLQIKV
7227_Dmelanogaster@NP_649840	VALYHENVVE	HYENPRNVGS	L-DKKDV-TV	GTGLVGAPAC	GDVMKLQIKV
6239_Celegans@NP_502658	VAQYHEKVID	HYENPRNVGS	L-DKNDP-SV	GTGIVGAPAC	GDVMKLQIRV

```
                                                   101
243233_Mcapsulatus@YP_112782              N-E-E----G --VIEDAKFK TYGCGSAIAS SSLVTEWVKG KTVDEALAIK
458817_Shalifaxensis@YP_001673798         D-D-N----G --IIEDAKFK TYGCGSAIAS SSLVTEWVKG KTVAEALAIK
398579_Spealeana@YP_001501349             D-D-N----G --IIEDAKFK TYGCGSAIAS SSLVTEWVKG KTVAEALAIK
557598_Lhongkongensis@YP_002796852        N-E-N----G --VIEDAKFK TYGCGSAIAS SSLVTEWVKG KTLDEALAIK
521006_Ngonorrhoeae@YP_002001906          N-D-E----G --IIEDAKFK TYGCGSAIAS SSLITEWVKG KSLDDALAIK
489653_Nlactamica@YP_004048810            N-D-E----G --IIEDAKFK TYGCGSAIAS SSLITEWVKG KSLDDALAIK
935589_Nmeningitidis@YP_005903962         N-D-E----G --IIEDAKFK TYGCGSAIAS SSLITEWVKG KSLDDALAIK
395495_Lcholodnii@YP_001790394            N-PLT----G --MIEDAKFK TYGCGSAIAS SSLVTEWVKG KTLDQALAIK
999541_Bgladioli@YP_004361365             G-S-D----G --VIEDAKFK TYGCGSAIAS SSLVTEWVKG KTLDQALAIK
983917_Rgelatinosus@YP_005439140          DPS-T----G --VIEDARFK TYGCGSAIAS SSLVTEWVKG KTLEQALAIK
1416914_Ppnomenusa@YP_008837319           N-E-A----G --VIEDAKFK TYGCGSAIAS SSLVTEWVKG KTLDQALAIK
1380774_Psp@YP_008881415                  N-E-A----G --VIEDAKFK TYGCGSAIAS SSLVTEWVKG KTLDQALAIK
1249661_Bthailandensis@YP_007917844       G-E-N----G --VIEDAKFK TYGCGSAIAS SSLVTEWVKG KTLDEALAIK
320388_Bmallei@YP_993528                  G-A-D----G --VIEDAKFK TYGCGSAIAS SSLVTEWVKG KTLDEALAIK
536230_Bpseudomallei@YP_002897469         G-A-D----G --VIEDAKFK TYGCGSAIAS SSLVTEWVKG KTLDEALAIK
395019_Bmultivorans@YP_001579332          G-A-D----G --VIEDAKFK TYGCGSAIAS SSLVTEWVKG KTLDEALAIK
626418_Bglumae@YP_002912250               G-T-D----G --VIEDAKFK TYGCGSAIAS SSLVTEWVKG KTLDEALAIK
365044_Pnaphthalenivor@YP_981803          N-A-E----G --VIEDAKFK TYGCGSAIAS SSLVTEWVRG RTLDEALAIK
266265_Bxenovorans@YP_553871              N-R-E----G --VIEDAKFK TYGCGSAIAS SSLVTEWVRG RTLDEALAIR
269482_Bvietnamiensis@YP_001115172        N-A-D----G --VIEDARFK TYGCGSAIAS SSLVTEWVRG RTLDEALAIK
583345_Mmobilis@YP_003049105              N-D-S----G --IIEDAKFK TYGCGSAIAS SSLVTEWLKG RTIEEAYAIK
666681_Mversatilis@YP_003674921           N-D-S----G --IIEDAKFK TYGCGSAIAS SSLVTEWLKG RTIEEAYAIK
391038_Bphymatum@YP_001857685             G-A-D----G --VIEDAKFK TYGCGSAIAS SSLVTEWVKG KTLDEALSIK
398577_Bambifaria@YP_001808732            S-A-D----G --VIEDAKFK TYGCGSAIAS SSLVTEWVKG KTLDEALSIK
269482_Bvietnamiensis@YP_001120041        G-A-D----G --VIEDAKFK TYGCGSAIAS SSLVTEWVKG KTLDEALSIK
406425_Bcenocepacia@YP_001765425          G-A-D----G --VIEDAKFK TYGCGSAIAS SSLVTEWVKG KTLDEALSIK
1009846_Bcepacia@YP_006615433             G-A-D----G --VIEDAKFK TYGCGSAIAS SSLVTEWVKG KTLDEALSIK
482957_Blata@YP_369669                    G-A-D----G --VIEDAKFK TYGCGSAIAS SSLVTEWVKG KTLDEALSIK
748280_Psp@YP_004848592                   N-E-N----G --VIEDAKFK TYGCGSAIAS SSLVTEWVKG KTLDEAMSIK
1229205_Bphenoliruptrix@YP_006834626      G-A-D----G --IIEDAKFK TYGCGSAIAS SSLVTEWVKG KTLDEAMSIK
1163617_Sdenitrificans@YP_008546117       N-K-D----G --VIEDAKFK TYGCGSAIAS SSLVTEWVKG KTLDEALTIK
266265_Bxenovorans@YP_559453              G-A-D----G --IIEDAKFK TYGCGSAIAS SSLVTEWVKG KTLDQAMSIK
398527_Bphytofirmans@YP_001896197         G-A-D----G --IIEDAKFK TYGCGSAIAS SSLVTEWVKG KTLDQAMSIK
375286_Jsp@YP_001352943                   G-A-D----G --VIQDAKFK TYGCGSAIAS SSLVTEWVKG KTLDQAMSIK
204773_Harsenicoxydans@YP_001100494       G-A-D----G --VIEDAKFK TYGCGSAIAS SSLVTEWVKG KTLDQALSIK
580332_Slithotrophicus@YP_003524869       G-K-D----G --LIEDAKFK TYGCGSAIAS SSLVTEWVKG KTVDQALAIK
159087_Daromatica@YP_285167               N-K-S----G --VIEDAKFK TYGCGSAIAS SSLVTEWVKG KTVDQALSIK
882378_Brhizoxinica@YP_004029636          G-A-D----G --VIEDAKFK TYGCGSAIAS SSLVTEWVKG KTLDQALSIK
85643_Tsp@YP_002355840                    G-K-D----G --VIEDAKFK TYGCGSAIAS SSLVTEWVKG KTLDQALDIK
757424_Hseropedicae@YP_003776230          D-H-N----G --IIEDAKFK TYGCGSAIAS SSLVTEWLKG RTLDQALDIK
522306_CAccumulibacter@YP_003167247       G-K-G----G --VIEDAKFK TYGCGSAIAS SSLVTEWVKG KTIDQALDIK
76114_Aaromaticum@YP_160687               G-K-D----G --VIEDAKFK TYGCGSAIAS SSLVTEWVKG KTVDQALEIK
381666_Reutropha@YP_725668                N-E-A----G --VIEDAKFK TYGCGSAIAS SSLVTEWVKG KTVDQALEIK
977880_Ctaiwanensis@YP_002005171          N-E-A----G --VIEDAKFK TYGCGSAIAS SSLVTEWVKG KTVDQALEIK
1042878_Cnecator@YP_004684939             N-E-A----G --VIEDAKFK TYGCGSAIAS SSLVTEWVKG KTVDQALEIK
266264_Cmetallidurans@YP_583181           N-E-A----G --VIEDAKFK TYGCGSAIAS SSLVTEWVKG KTVDQALEIK
395494_Gcapsiferriform@YP_003846450       G-K-D----G --IIEDAKFK TYGCGSAIAS SSLVTEWVKG KTVDQALQIK
859655_Rsolanacearum@YP_005996961         N-E-Q----G --VIEDAKFK TYGCGSAIAS SSLVTEWVKG KTLDQALEIR
402626_Rpickettii@YP_001898467            N-E-Q----G --VIEDAKFK TYGCGSAIAS SSLVTEWVKG KTLDQALEIR
543913_bproteobacteriu@YP_007502653       N-D-Q----G --VIEDAKFK TYGCGSAIAS SSLVTEWVKG KTLDQALEIK
62928_Asp@YP_933519                       G-K-D----G --VIEDAKFK TYGCGSAIAS SSLVTEWVKG KTLDQALEIK
640081_Dsuillum@YP_005028573              N-K-E----G --VIEDAKFK TYGCGSAIAS SSLVTEWVKG KSLDQALEIK
452638_Pnecessarius@YP_001797351          N-D-Q----G --VIEDAKFK TYGCGSAIAS SSLVTEWVKG KTLDQAFEIK
292415_Tdenitrificans@YP_314923           G-K-D----G --LIEDAKFK TYGCGSAIAS SSLVTEWVKG KTLDQAMEIK
265072_Mflagellatus@YP_544916             N-D-Q----G --IIEDAKFK TYGCGSAIAS SSLVTELIKG KSLDQAMEIK
582744_Mglucosetrophus@YP_003051619       N-E-A----G --VIEDAKFK TYGCGSAIAS SSLVTEWIKG KTLDQVMEIK
887061_Msp@YP_004040241                   N-E-A----G --VIEDAKFK TYGCGSAIAS SSLVTEWIKG KTLDQVMEIK
891398_CTremblaya@YP_004707015            R---H----G --MVDEVRFQ TYGCGSARAA SSLVARWIEG RPLEEALEVR
426114_Tarsenitoxydans@YP_003623866       N-D-Q----G --VIEDARFK TYGCGSAIAS SSLVTEWVKG KTLDQALEIR
75379_Tintermedia@YP_003642782            N-D-Q----G --VIEDARFK TYGCGSAIAS SSLVTEWVKG KTLDQALEIR
937774_Tequigenitalis@YP_004130301        N-D-S----G --IIEDAKFK TYGCGSAIAS SSLVTEWVKG KSLDQAMDIK
1008459_Tasinigenitalis@YP_004874474      N-D-A----G --IIEDARFK TYGCGSAIAS SSLVTEWVKG KSLDQAMDIK
634503_Eictaluri@YP_002934567             N-N-E----G --IIEDARFK TYGCGSAIAS SSLVTEWVKG KSLEQAAEIK
718251_Etarda@YP_005700147                N-N-E----G --IIEDARFK TYGCGSAIAS SSLVTEWVKG KSLEQAAEIK
259536_Parcticus@YP_264760                D-D-N----G --IIEDARFK TYGCGSAIAS SSLVTEWLKG KSLDQAGEIK
335284_Pcryohalolentis@YP_580918          D-D-N----G --IIEDARFK TYGCGSAIAS SSLVTEWLKG KSLDQAGEIK
571800_Psp@YP_008163576                   N-D-N----G --IIEDARFK TYGCGSAIAS SSLVTEWLKG KSLDQAGEIK
420662_Mpetroleiphilum@YP_001021452       NPA-T----G --LIEDAKFK TYGCGSAIAS SSLVTEWVKG KSLDEALTIK
243365_Cviolaceum@NP_900763               G-A-D----G --VIEDAKFK TYGCGSAIAS SSLVTEWVKG KSLDEALAIK
1208919_CKinetoplastiba@YP_007459239      N-D-D----G --FIEDAKFK TYGCGSAIAS SSLVTEWVKG KSIDQALSIR
983545_Gsp@YP_004435367                   G-A-T----G --IIEDAKFK TYGCGSAIAS SSLVTEWVKG KSIDDAVKIS
342610_Patlantica@YP_660819               G-A-N----G --IIEDAKFK TYGCGSAIAS SSLVTEWVKG KSIDDAVKIS
743299_Aferrivorans@YP_004783591          N-G-Q----G --IIEDAKFK TYGCGSAIAS SSLVTEWVKG KTLDEAMTIR
243159_Aferrooxidans@YP_002425162         N-G-Q----G --IIEDAKFK TYGCGSAIAS SSLVTEWVKG KTLDEAMAIK
1005048_Cfungivorans@YP_004753777         G-A-D----G --IIQDAKFK TYGCGSAIAS SSLVTEWVKG KTLDQAMSIK
669502_CProfftella@YP_008343646           N-K-N----G --VITDAKFK TYGCGSAIAS SSLVTEWVKG KTLDEAMSIK
757424_Hseropedicae@YP_003776535          G-A-D----G --VIQDAKFK TYGCGSAIAS SSLVTEWVKG KTLDEALSIK
395495_Lcholodnii@YP_001790080            DPA-T----G --KIADARFK TYGCGSAIAS SSLVTEWVKG KTLDEALTIK
257310_Bbronchiseptica@NP_888820          N-E-A----G --VIEDARFK TYGCGSAIAS SSLVTEWVKG KTLDEAMNIR
990288_Acaldus@YP_004748177               N-A-Q----G --IIEDARFK TYGCGSAIAS SSLVTEWVKG KTLDEAMTIK
990288_Acaldus@YP_004748110               N-E-E----G --IIEDARFK TYGCGSAIAS SSLVTEWVKG KTLDEALTIQ
983917_Rgelatinosus@YP_005436383          DPS-T----G --LIEDARFK TYGCGSAIAS SSLVTEWVKG KTLDQALTIK
360910_Bavium@YP_786035                   N-S-E----G --VIEDARFK TYGCGSAIAS SSLVTEWVKG KTLDQALSIR
1036672_Akashmirensis@YP_006379415        N-K-E----G --VIEDARFK TYGCGSAIAS SSLVTEWVKG KTLDQALDIR
340100_Bpetrii@YP_001631386               S-E-S----G --VIEDARFK TYGCGSAIAS SSLVTEWVKG KTLDEALDIR
523791_Kkoreensis@YP_003146685            N-E-Q----G --IIEDAKFK TYGCGSAIAS SSLITEWVKG KTVDEAQTIK
156889_Mmarinus@YP_866958                 D-D-N----G --VIEDAKFK TFGCGSAIAS SSLVTEWIKG KTIDEALTIK
```

```
404589_Asp@YP_001377835                 N-G-E----G --VIEDAKFK TFGCGSAIAS SSLVTEWVKG KTVDEALTIK
290397_Adehalogenans@YP_463806          S-D-D----G --IIEDAKFK TFGCGSAIAS SSLVTEWVKG RTVEEALTIK
447217_Asp@YP_002132996                 S-D-D----G --VIEDAKFK TFGCGSAIAS SSLVTEWVKG RTVEEALTIK
455488_Adehalogenans@YP_002491042       S-D-D----G --VIEDAKFK TFGCGSAIAS SSLVTEWVKG RTVEEALTIK
378806_Saurantiaca@YP_003955403         T-D-E----G --VIEDAKFK TFGCGSAIAS SSLVTEWVKG KTVDQAMTIS
246197_Mxanthus@YP_633158               S-E-D----G --LIEDARFK TFGCGSAIAS SSLVTEWVKG KTVDQAMTIS
483219_Mfulvus@YP_004669542             S-E-D----G --LIEDARFK TFGCGSAIAS SSLVTEWVKG KTVDQAMTIS
1144275_Ccoralloides@YP_005368444       S-D-T----G --VIEDARFK TFGCGSAIAS SSLVTEWVKG KTVDQAMTIS
1278073_Mstipitatus@YP_007362455        S-D-D----G --LIEDARFK TFGCGSAIAS SSLVTEWVKG KTVDQAMTIS
448385_Scellulosum@YP_001617979         G-E-G----G --VIEDAKFK TFGCGSAIAS SSLATEWLKG KTIDEAETIK
399739_Pmendocina@YP_001188990          N-E-S----G --VIEDAKFK TYGCGSAIAS SSLATEWMKG KTLDEAESIK
1294143_Pdenitrificans@YP_007659193     N-E-Q----G --VIEDAKFK TYGCGSAIAS SSLATEWMKG KTLDEAESIK
208964_Paeruginosa@NP_252502            N-E-Q----G --VIEDAKFK TYGCGSAIAS SSLATEWMKG KTLDEAETIK
322710_Avinelandii@YP_002801150         N-E-Q----G --IIEDAKFK TYGCGSAIAS SSLATEWMKG RTLEEAETIK
644801_Pstutzeri@YP_007239445           N-E-Q----G --VIEDAKFK TYGCGSAIAS SSLATEWMKG KTLEEAETIK
1207075_Psp@YP_007027941                N-D-A----G --IIEDAKFK TYGCGSAIAS SSLATEWMKG KTLDEAETIK
1114970_Pfluorescens@YP_005206437       N-E-Q----G --IIEDAKFK TYGCGSAIAS SSLATEWMKG KTLDEAETIK
220664_Pprotegens@YP_262044             N-E-Q----G --IIEDAKFK TYGCGSAIAS SSLATEWMKG KTLDEAETIK
351746_Pputida@YP_001266219             N-E-Q----G --VIEDAKFK TYGCGSAIAS SSLATEWMKG KTLDEAETIK
1435058_Pmonteilii@YP_008958359         N-E-Q----G --VIEDAKFK TYGCGSAIAS SSLATEWMKG KTLDEAETIK
743720_Pfulva@YP_004475358              N-E-Q----G --VIEDAKFK TYGCGSAIAS SSLATEWMKG KTLDEAETIK
264730_Psyringae@YP_273568              N-E-Q----G --VIEDAKFK TYGCGSAIAS SSLATEWMKG KTLDEAETIK
384676_Pentomophila@YP_606728           N-E-Q----G --VIEDAKFK TYGCGSAIAS SSLATEWMKG KTLDEAETIK
1430440_Mgryphiswaldens@YP_008937260    S-P-E----G --IIEDAKFK TFGCGSAIAS SSLVTEWVKG KTLDEAAAIK
749219_Mcatarrhalis@YP_003626770        S-D-D----G --IIEDAKFK TYGCGSAIAS SSLVTEWLKG KTLDQAAAIK
62977_Asp@YP_046090                     N-N-D----G --VIEEARFK TYGCGSAIAS SSLVTEWLKG KTLDQAQAIK
436717_Aoleivorans@YP_003732380         N-D-S----G --VIEEARFK TYGCGSAIAS SSLVTEWLKG KTLDEAQAIK
400667_Abaumannii@YP_001084660          N-D-N----G --VIEEARFK TYGCGSAIAS SSLVTEWLKG KTLDEAQAIK
871585_Acalcoaceticus@YP_004995269      N-D-S----G --VIEEARFK TYGCGSAIAS SSLVTEWLKG KTLDEAQAIK
511062_Osp@YP_005091814                 S-E-D----G --IIEDAKFK TYGCGSAIAS SSLITEWVKG KSLDEAASIK
318167_Sfrigidimarina@YP_751106         N-A-D----G --IIEDAKFK TYGCGSAIAS SSLVTEWVKG KSVDEAATIK
326297_Samazonensis@YP_927171           N-D-A----G --IIEDAKFK TYGCGSAIAS SSLVTEWVKG KSIEEAAAIK
318161_Sdenitrificans@YP_562467         S-A-D----G --IIEDAKFK TYGCGSAIAS SSLVTEWVKG KSVEQAGSIK
167879_Cpsychrerythrae@YP_267876        S-D-D----G --IIEDAKFK TYGCGSAIAS SSLVTEWVKG KSIEEAGEIK
637905_Sviolacea@YP_003556341           D-D-K----G --MIQDAKFK TYGCGSAIAS SSLITEWVKG KSIEEAAAIK
225849_Spiezotolerans@YP_002311055      D-D-N----G --IIEDAKFK TYGCGSAIAS SSLVTEWVKG KTVEEAGAIK
323850_Sloihica@YP_001094441            D-D-N----G --IIEDAKFK TYGCGSAIAS SSLVTEWVKG KTIEEAQAIK
425104_Ssediminis@YP_001474605          D-K-D----G --MIEDAKFK TYGCGSAIAS SSLVTEWVKG KTIEEAAAIK
392500_Swoodyi@YP_001760156             D-A-D----G --VIEDAKFK TYGCGSAIAS SSLVTEWVKG KTIEEAAAIK
60481_Ssp@YP_737869                     G-A-D----G --IIEDAKFK TYGCGSAIAS SSLVTEWVKG KTIEQAAAIK
211586_Soneidensis@NP_717861            G-A-D----G --IIEDAKFK TYGCGSAIAS SSLVTEWVKG KTIEQAAAIK
693973_Sbaltica@YP_005273706            G-A-D----G --IIQDAKFK TYGCGSAIAS SSLVTEWVKG KTIEQAAAIK
399804_Sputrefaciens@YP_006009782       G-A-D----G --IIQDAKFK TYGCGSAIAS SSLVTEWVKG KTIEQAAAIK
298386_Pprofundum@YP_128966             S-D-E----G --IIEDAKFK TYGCGSAIAS SSLITEWVKG KSLDEAAAIK
28173_Vnigripulchritu@YP_008621944      S-P-E----G --IIEDAKFK TYGCGSAIAS SSLVTEWVKG KSIDEAAAIK
1219076_Valginolyticus@YP_008537102     T-P-E----G --IIEDAKFK TYGCGSAIAS SSLVTEWVKG KSVDEAAAIK
150340_Vsp@YP_003286982                 T-P-E----G --IIEDAKFK TYGCGSAIAS SSLVTEWVKG KSVDEAAAIK
345073_Vcholerae@YP_001216234           S-P-E----G --IIEDAKFK TYGCGSAIAS SSLVTEWVKG KSIDEAAAIK
223926_Vparahaemolytic@NP_796976        T-P-E----G --IIEDAKFK TYGCGSAIAS SSLVTEWVKG KSIDEAAAIK
882102_Vanguillarum@YP_004566935        T-P-E----G --IIEDAKFK TYGCGSAIAS SSLVTEWVKG KSIDEAAAIK
575788_Vsplendidus@YP_002416251         S-A-E----G --IIEDAKFK TYGCGSAIAS SSLVTEWVKG KSIDEAAAIK
1129794_Gpsychrophila@YP_007545568      D-K-N----G --IIEDAKFK TYGCGSAIAS SSLVTEWVKG KSIDEASTIS
196600_Vvulnificus@NP_933549            T-P-E----G --IIEDAKFK TYGCGSAIAS SSLVTEWVKG KSIEEAASIK
903510_Vfurnissii@YP_004993913          S-P-E----G --IIEDAKFK TYGCGSAIAS SSLVTEWVKG KSIDEAASIK
338187_Vcampbellii@YP_001444276         T-P-E----G --IIEDAKFK TYGCGSAIAS SSLVTEWVKG KSIDEAASIK
338187_Vcampbellii@YP_008526292         T-P-E----G --IIEDAKFK TYGCGSAIAS SSLVTEWVKG KSIDEAASIK
314282_Psp@YP_007639631                 N-K-D----G --IIEDAKFK TYGCGSAIAS SSLVTEWVKG KTLEEAGAIT
357804_Pingrahamii@YP_942749            G-E-N----G --LIEDAKFK TYGCGSAIAS SSLVTEWVKG KTLEQAGTIS
326442_Phaloplanktis@YP_341160          S-A-D----G --IIEDAKFK TYGCGSAIAS SSLVTEWVKG KTLEQAATIK
234831_Psp@YP_004067531                 S-A-E----G --VIEDAKFK TYGCGSAIAS SSLVTEWVKG KTLDEAATIK
595494_Tauensis@YP_002893203            N-E-D----G --VIEDAKFK TYGCGSAIAS SSLVTEWVKG KTLEEAAAIK
396588_Tsulfidiphilus@YP_002513559      N-A-E----G --VIEDAKFK TYGCGSAIAS SSLLTEWVKG KTLDEAAAIK
396588_Tsulfidiphilus@YP_002514125      N-A-E----G --VIEDAKFK TYGCGSAIAS SSLLTEWVKG KTLDEAAAIK
998088_Averonii@YP_004393031            S-D-E----G --IIEDAKFK TYGCGSAIAS SSLVTEWVKG KTLDEAAGIK
380703_Ahydrophila@YP_856284            S-D-D----G --IIEDAKFK TYGCGSAIAS SSLVTEWVKG KTLDEAAGIK
382245_Asalmonicida@YP_001142383        S-D-D----G --IIEDAKFK TYGCGSAIAS SSLVTEWVKG KTLDEAAGIK
342108_Mmagneticum@YP_422390            S-A-E----G --IIEDAKFK TFGCGSAIAS SSLVTEWVKG KTLDEAASIK
1245471_Presinovorans@YP_008101472      N-E-Q----G --VIEDAKFK TYGCGSAIAS SSLATEWMKG KTLDEAASIK
365044_Pnaphthalenivor@YP_982518        NPI-T----G --VIEDARFK TYGCGSAIAS SSLVTEWVKG KTLDQAASIK
296591_Psp@YP_549004                    NPA-T----G --VIEDARFK TYGCGSAIAS SSLVTEWVKG KTLDEAASIK
232721_Asp@YP_986388                    NPD-T----G --VIEDARFK TYGCGSAIAS SSLVTEWVKG KTLDEAASLK
535289_Aebreus@YP_002553097             NPD-T----G --VIEDARFK TYGCGSAIAS SSLVTEWVKG KTLDEAASLK
365046_Rtataouinensis@YP_004618947      NPA-T----G --VIEDARFK TYGCGSAIAS SSLVTEWVKG KTLDEAASLK
946483_CSymbiobacter@YP_008681130       SPD-T----G --KIEDARFK TFGCGSAIAS SSLVTEWVKG KTLDEAVALR
312309_Vfischeri@YP_204001              S-P-E----G --VIEDAKFK TYGCGSAIAS SSLVTEWVKG KTLDEAAALK
595537_Vparadoxus@YP_004156043          NPE-T----G --VIEDARFK TYGCGSAIAS SSLVTEWVKG KTLDEAAALK
316275_Asalmonicida@YP_002262235        S-P-E----G --IIEDAKFK TYGCGSAIAS SSLVTEWVKG KTLDEAAALK
391735_Veiseniae@YP_997138              NPQ-T----G --VIEDARFK TYGCGSAIAS SSLVTEWVKG KTLDEAAAVK
397945_Acitrulli@YP_970793              NPE-T----G --VIEDARFK TYGCGSAIAS SSLVTEWVKG KTLDEAAALK
643561_Aavenae@YP_004235248             NPE-T----G --VIEDARFK TYGCGSAIAS SSLVTEWVKG KTLDEAAALK
398578_Dacidovorans@YP_001565014        NPE-T----G --VIEDARFK TYGCGSAIAS SSLVTEWVKG KTLDEAAALK
742013_Dsp@YP_004488168                 NPE-T----G --VIEDARFK TYGCGSAIAS SSLVTEWVKG KTLDEAAALK
338969_Aferrireducens@YP_523433         NSE-T----G --VIEDARFK TYGCGSAIAS SSLVTEWVKG KTLDQAAALK
596154_Adenitrificans@YP_004388278      NPE-T----G --VIEDARFK TYGCGSAIAS SSLVTEWVKG KTLDEAAALK
688245_Ctestosteroni@YP_003278774       NPA-T----G --VIEDARFK TYGCGSAIAS SSLVTEWVKG KTLDEAAALK
1266738_Pmirabilis@YP_008398502         D-D-N----G --VIEDARFK TYGCGSAIAS SSLVTEWMKG KTLDEAEAIK
1124991_Mmorganii@YP_007505117          D-D-N----G --VIEDARFK TYGCGSAIAS SSLVTEWMKG KTLDEAEAIK
406817_Xnematophila@YP_003713469        N-N-E----G --IIEDARFK TYGCGSAIAS SSLVTEWMKG KSLEQAEAIK
```

```
291112_Pasymbiotica@YP_003040200        N-N-E----G --IIEDARFK TYGCGSAIAS SSLVTEWMKG KSLEQAEAIK
243265_Pluminescens@NP_930506           N-N-E----G --IIEDARFK TYGCGSAIAS SSLVTEWMKG KSLEQAEAIK
406818_Xbovienii@YP_003468915           N-N-E----G --IIEDARFK TYGCGSAIAS SSLVTEWMKG KSLEQAEAIK
273123_Ypseudotubercul@YP_071365        N-E-A----G --IIEDARFK TYGCGSAIAS SSLVTEWMKG KSLEQAEAIK
393305_Yenterocolitica@YP_001005390     N-E-A----G --IIEDARFK TYGCGSAIAS SSLVTEWMKG KSLDQAETIK
1346614_Sliquefaciens@YP_008231656      N-N-E----G --IIEDARFK TYGCGSAIAS SSLVTEWMKG KSLDQAEAIK
1348660_Splymuthica@YP_008160622        N-N-E----G --IIEDARFK TYGCGSAIAS SSLVTEWMKG KSLDQAEAIK
399741_Sproteamaculans@YP_001479850     N-N-E----G --IIEDARFK TYGCGSAIAS SSLVTEWMKG KSLDQAEAIK
1249634_Smarcescens@YP_007345780        N-D-A----G --IIEDARFK TYGCGSAIAS SSLVTEWMKG KSLDQAEAIK
187410_Ypestis@NP_668657                N-E-A----G --IIEDARFK TYGCGSAIAS SSLVTEWMKG KSLDQAEAIK
1157951_Pstuartii@YP_006215182          N-D-N----G --IIEDARFK TYGCGSAIAS SSLVTEWMKG KSLDEAEAIK
344609_Sboydii@YP_001881320             N-D-E----G --IIEDARFK TYGCGSAIAS SSLVTEWVKG KSLVEAQAIK
104623_Ssp@YP_008524031                 N-N-D----G --IIEDARFK TYGCGSAIAS SSLVTEWVKG KSLSEAEAIK
561229_Dzeae@YP_003003456               N-E-Q----G --IIEDARFK TYGCGSAIAS SSLVTEWVKG KSLNEAESIK
590409_Ddadantii@YP_003334610           N-E-Q----G --IIEDARFK TYGCGSAIAS SSLVTEWVKG KSLTEAESIK
561230_Pcarotovorum@YP_003018589        N-N-E----G --IIEDARFK TYGCGSAIAS SSLVTEWVKG KSLNEAEAIK
561231_Pwasabiae@YP_003258666           N-N-E----G --IIEDARFK TYGCGSAIAS SSLVTEWVKG KSLNEAEAIK
1166016_Psp@YP_006282128                N-N-E----G --IIEDARFK TYGCGSAIAS SSLVTEWVKG KSLNEAEAIK
218491_Patrosepticum@YP_051325          N-N-E----G --IIEDARFK TYGCGSAIAS SSLVTEWVKG KSLNEAEAIK
99287_Senterica@NP_461477               N-D-E----G --IIEDARFK TYGCGSAIAS SSLVTEWVKG KSLDEAQAIK
218493_Sbongori@YP_004731144            N-D-E----G --IIEDARFK TYGCGSAIAS SSLVTEWVKG KSLDEAQAIK
290339_Csakazakii@YP_001436836          N-N-E----G --IIEDARFK TYGCGSAIAS SSLVTEWVKG KSLDEAQAIK
693216_Cturicensis@YP_003211492         N-N-E----G --IIEDARFK TYGCGSAIAS SSLVTEWVKG KSLDEAQAIK
399742_Esp@YP_001177741                 N-N-E----G --IIEDARFK TYGCGSAIAS SSLVTEWVKG KSLDEAQAIK
640513_Easburiae@YP_004829762           N-N-E----G --IIEDARFK TYGCGSAIAS SSLVTEWVKG KSLDEAQAIK
693444_Ebacterium@YP_007339349          N-N-E----G --IIEDARFK TYGCGSAIAS SSLVTEWVKG KSLDEAQAIK
290338_Ckoseri@YP_001451852             N-N-E----G --IIEDARFK TYGCGSAIAS SSLVTEWVKG KSLDEAQAIK
484021_Kpneumoniae@YP_002920722         N-N-E----G --IIEDARFK TYGCGSAIAS SSLVTEWVKG KSLDEAQAIK
637910_Crodentium@YP_003366009          N-N-E----G --IIEDARFK TYGCGSAIAS SSLVTEWVKG KSLDEAQAIK
640131_Kvariicola@YP_003438138          N-N-E----G --IIEDARFK TYGCGSAIAS SSLVTEWVKG KSLDEAQAIK
1191061_Koxytoca@YP_006500071           N-N-E----G --IIEDARFK TYGCGSAIAS SSLVTEWVKG KSLDEAQAIK
1286170_Rornithinolytic@YP_007872306    N-N-E----G --IIEDARFK TYGCGSAIAS SSLVTEWVKG KSLDEAQAIK
511145_Ecoli@NP_417024                  N-D-E----G --IIEDARFK TYGCGSAIAS SSLVTEWVKG KSLDEAQAIK
585054_Efergusonii@YP_002381825         N-D-E----G --IIEDARFK TYGCGSAIAS SSLVTEWVKG KSLDEAQAIK
198214_Sflexneri@NP_708368              N-D-E----G --IIEDARFK TYGCGSAIAS SSLVTEWVKG KSLDEAQAIK
216599_Ssonnei@YP_005457544             N-D-E----G --IIEDARFK TYGCGSAIAS SSLVTEWVKG KSLDEAQAIK
300267_Sdysenteriae@YP_404261           N-D-E----G --IIEDARFK TYGCGSAIAS SSLVTEWVKG KSLDEAQAIK
716541_Ecloacae@YP_003614361            N-N-E----G --IIEDARFK TYGCGSAIAS SSLVTEWVKG KSLDEAQAIK
701347_Elignolyticus@YP_003940771       N-N-E----G --IIEDARFK TYGCGSAIAS SSLVTEWVKG KSLDEAQAIK
630626_Sblattae@YP_006318650            N-N-D----G --IIEDARFK TYGCGSAIAS SSLVTEWVKG KSLDEAQAIK
1028307_Eaerogenes@YP_004590373         N-N-E----G --IIEDARFK TYGCGSAIAS SSLVTEWVKG KSLDEAQAIK
557723_Hparasuis@YP_002474707           N-E-Q----G --IIEDARFK TYGCGSAIAS SSLITEWVKG KSLDEAQAIK
1005058_Ganatis@YP_004420698            N-D-N----G --IIEDAKFK TYGCGSAIAS SSLITEWVKG KSLDEAQAIK
1075089_Pmultocida@YP_005176279         S-E-E----G --IIEDAKFK TYGCGSAIAS SSLITEWVKG KSLDEAQAIK
205914_Hsomnus@YP_718490                N-D-E----G --IIEDAKFK TYGCGSAIAS SSLITEWVKG KSLDEAQAIK
1171377_Btrehalosi@YP_007548422         N-D-S----G --IIEDARFK AYGCGSAIAS SSLITEWVKG KSLDEAAAIK
233412_Hducreyi@NP_873560               N-D-D----G --IIEDARFK AYGCGSAIAS SSLITEWVKG KSLDEAGAIK
416269_Apleuropneumoni@YP_001053631     N-D-Q----G --IIEDARFK AYGCGSAIAS SSLITEWVKG KSLEEAGAIK
696748_Asuis@YP_006816777               N-D-Q----G --IIEDARFK AYGCGSAIAS SSLITEWVKG KSLEEAGAIK
1366053_Mhaemolytica@YP_008338465       N-D-Q----G --IIEDARFK AYGCGSAIAS SSLITEWVKG KSLEEAGAIK
1334187_Hinfluenzae@YP_008544790        D-D-N----G --IIEDAKFK TYGCGSAIAS SSLITEWVKG KSLEEAGAIK
634176_Aaphrophilus@YP_003007650        N-D-E----G --IIEDAKFK TYGCGSAIAS SSLITEWVKG KSLEEAGAIK
694569_Aactinomycetemc@YP_006286355     N-D-D----G --IIEDAKFK TYGCGSAIAS SSLITEWVKG KSLEEAGAIK
862965_Hparainfluenzae@YP_004822800     N-D-S----G --IIEDAKFK TYGCGSAIAS SSLITEWVKG KSLDEAGAIK
221988_Msucciniciprodu@YP_088916        N-E-E----G --IIEDAKFK TYGCGSAIAS SSLITEWVKG KSLDEAGAIK
339671_Asuccinogenes@YP_001344170       N-D-N----G --IIEDAKFK TYGCGSAIAS SSLITEWVKG KSLDEAGAIK
550540_Fbalearica@YP_003914035          S-E-G----G --VIEDAKFK TYGCGSAIAS SSLVTEWVKG KTLDEAEQIK
396595_Tsp@YP_003460082                 N-D-D----G --VIEDAKFK TYGCGSAIAS SSLLTEWVRG KTLEEASQIK
413404_CRuthia@YP_903798                D-D-N----G --VIEDAKFK TYGCGSAIAS SSLLTEWVKG KTLNEAAEIK
412965_CVesicomyosociu@YP_001219373     D-D-N----G --VIEDAKFK TYGCGSAIAS SSLLTQWVKG KTLDEATKIK
1208921_CKinetoplastiba@YP_007448562    N-G-D----G --IIEDACFK TYGCGSAIAS SSLVTEWIKG KSLTQAEEIS
561501_Baphidicola@YP_002468877         N-E-K----G --IIEDACFK TYGCGSAIAS SSLVTEWVKG KSIEEAESIK
357244_Otsutsugamushi@YP_001248706      N-D-H----G --IIEDAKFK TFGCGSAIAS SALVTEWIKS QSLSDAEQIK
1003201_Rtyphi@YP_005427548             D-D-D----E --IITDAKFK TFGCGSAIAS SSLVTELVKG RSVEDAQEIK
1105111_CRickettsia@YP_005365544        D-D-D----G --IITDAKFK TFGCGSAIAS SSLVTEWVKG RSVEDAEIIK
272944_Rconorii@NP_360366               D-D-D----G --IITDAKFK TFGCGSAIAS SSLVTEWVKG RSVEDAETIK
452659_Rrickettsii@YP_001650107         D-D-D----G --IITDAKFK TFGCGSAIAS SSLVTEWVKG RSVEDAETIK
347255_Rafricae@YP_002845312            D-D-D----G --IITDAKFK TFGCGSAIAS SSLVTEWVKG RSVEDAETIK
562019_Rpeacockii@YP_002916645          D-D-D----G --IITDAKFK TFGCGSAIAS SSLVTEWVKG RSVEDAETIK
1105113_Rrhipicephali@YP_005390607      D-D-D----G --IITDAKFK TFGCGSAIAS SSLVTEWVKG RSVEDAETIK
416276_Rmassiliae@YP_001499486          D-D-D----G --IITDAKFK TFGCGSAIAS SSLVTEWVKG RSVEDAETIK
1105108_Rparkeri@YP_005392983           D-D-N----G --IITDAKFK TFGCGSAIAS SSLVTEWVKG RSVEDAETIK
1105114_Rmontanensis@YP_005391246       D-D-D----G --VITDAKFK TFGCGSAIAS SSLVTEWVKG RSVEDAETIK
1032845_Rheilongjiangen@YP_004764467    D-D-D----G --IITDAKFK TFGCGSAIAS SSLVTEWVKG RSVEDAETIK
652620_Rjaponica@YP_004884928           D-D-D----G --IITDAKFK TFGCGSAIAS SSLVTEWVKG RSVEDAETIK
481009_Rphilipii@YP_005300823           D-D-D----G --IITDAKFK TFGCGSAIAS SSLVTEWVKG RSVEDAETIK
1105109_Rslovaca@YP_005426472           D-D-D----G --IITDAKFK TFGCGSAIAS SSLVTEWVKG RSVENAETIK
1105107_Rcanadensis@YP_005299497        D-D-N----G --IITDAKFK TFGCGSAIAS SSLVTEWVKG RSINDAETIK
336407_Rbellii@YP_538116                D-D-N----G --IITDAKFK TFGCGSAIAS SSLVTEWVKG RSIDDAEEIK
315456_Rfelis@YP_246860                 D-D-D----G --IITDAKFK TFGCGSAIAS SSLVTEWVKG RSINDAGEIK
293614_Rakari@YP_001493572              D-D-D----G --IITDAKFK TFGCGSAIAS SSLVTEWVKG RSINDAGEIK
1105110_Raustralis@YP_005414962         D-D-D----G --IITDAKFK TFGCGSAIAS SSLVTEWVKG RSINDAGEIK
434131_Nristicii@YP_003081518           D-N-N----G --CIVDAKFK TFGCGSAIAS SSLATEYLIG KSIEEAEKIK
222891_Nsennetsu@YP_506192              D-N-N----G --CIVDAKFK TFGCGSAIAS SSLATEYLIG KSIEEAEKIK
330214_CNitrospira@YP_003799619         E-N-D----T --IV-DAKFK TFGCGSAIAS SSLATEWLKG KTVEEAGKIK
984262_Sgrandis@YP_005322201            S-E-D----G --IIQEAKFK TFGCGSAIAS SSLATEWLKG KSVDEAASID
100901_Wendosymbiont@YP_006555868       N-D-K----G --VIEDAKFK TFGCGSAIAS SSLLTEMIKG KAIKDATQIK
66084_Wsp@YP_002727379                  N-D-K----G --VIEDAKFK TFGCGSAIAS SSLLTEMIKG RTISDVTQIK
```

```
1423892_Emuris@YP_008929080              D-D-N----G --RIIDAKFK TFGCGAAIAA SSLATELIKG KTIDEAQQLK
574556_Acentrale@YP_003328545            D-E-N----G --TIVDAKFK TFGCGAAIAA SSLATERIKG KTIEEACMLK
320483_Amarginale@YP_002563600           D-E-N----G --TIVDAKFK TFGCGAAIAA SSLATERIKG KTIEEACMLK
1184253_Aphagocytophilu@YP_008332775     D-E-N----G --KIVDAKFK TFGCGAAIAA SELATERIMG KTIDEASLLK
205920_Echaffeensis@YP_507440            D-D-N----G --KIIDAKFK TFGCGAAIAA SSLATELIKG KTIDEAHQLK
254945_Eruminantium@YP_180281            D-D-N----G --KIIDAKFK TFGCGAAIAA SSLATELIKG KTVDEAHELK
254945_Eruminantium@YP_197308            D-D-N----G --KIIDAKFK TFGCGAAIAA SSLATELIKG KTVDEAHELK
269484_Ecanis@YP_303050                  D-N-N----G --RIIDAKFK TFGCGAAIAA SSLATELIKG KTVDEAHQLK
517418_Cthalassium@YP_001997584          D-EKT----G --IIEDARFK TFGCGSAIAS SSLATEMLKG KTIDEASEIK
485917_Pheparinus@YP_003091837           D-E-N----N --VITDAKFK TFGCGSAIAS SSLATEWLKG KSIDEALTID
762903_Psaltans@YP_004273747             D-E-N----N --VITDAKFK TFGCGSAIAS SSLATEWLKG KTIDEAVTID
929556_Scanadensis@YP_006255074          D-E-N----N --LITDAKFK TFGCGSAIAS SSLATEWLKG KTIDQALEID
760192_Hhydrossis@YP_004446110           DPA-T----S --TIVDAKFK TFGCGSAIAS SSLATEWLKG KTIDQALTID
1162668_Lferrooxidans@YP_005469656       N-D-D----G --IIEEAKFK TFGCGSAIAS SSLATEWVKG KTIDEALKIK
1048260_Lferriphilum@YP_006765680        N-D-A----G --VIEEAKFK TFGCGSAIAS SSLATEWVKG KTVEEALQIK
240015_Acapsulatum@YP_002755573          NPE-T----Q --VIEEAKFK TFGCGSAIAS SSLATEWVKG KTVEEALQIK
204669_CKoribacter@YP_589562             NPE-T----N --IIEDAKFK TFGCGSAIAS SSLATEWVKG KTVDEALGIK
1198114_Gtundricola@YP_004219465         NPE-T----Q --VIEDAKFK TFGCGSAIAS SSLATEWVKG KTVAEALQIS
926566_Troseus@YP_006423699              NPD-T----Q --VIEDAKFK TFGCGSAIAS SSLATEWVKG KTVAEALAIS
401053_Tsaanensis@YP_004180969           NPT-T----Q --IIEDAKFK TFGCGSAIAS SSLATEWVKG KTVAEALAIS
682795_Gmallensis@YP_005055878           NPE-T----Q --VIEDAKFK TFGCGSAIAS SSLATEWVKG KTVAEALTIS
696127_CMidichloria@YP_004679282         N-PKT----Q --IIEDVKFK TFGCLSAIAS SSLATERVKG KTLAEAARIK
871271_CZinderia@YP_003878048            Y---K----G --IIKNIRFK TYGCGSAIAS SYLTTKIIKN RTIEETLLIK
657324_Bfibrisolvens@YP_007819038        N---D----E -QIIEDVKFE TFGCGSAIAS SSMATELIMG KPVSEALQLT
768670_Cnitroreducens@YP_004051749       D---D----N -GVIQDVKFK TFGCGAAIAS SSMATELLKG KTVEEVLELT
717231_Fsinusarabici@YP_004603876        N---D----D -GVVEDVKFK TFGCGAAIAS SSMATELLKG KTVEEILELT
639282_Ddesulfuricans@YP_003496772       N---D----E -GIVEDVKFK TFGCGAAIAS SSMATELIKG KRVEDVLRLT
522772_Dacetiphilus@YP_003503972         N---D----D -MIVEDVKFK TFGCGAAIAS SSMATELMIG KSVEELLQLT
693746_Ovalericigenes@YP_004880314       D---K----D TQTIRDVKFN TFGCGSAIAT SSMATEMIKG KPVKDALALS
657321_Rbromii@YP_007781362              D---N----D --IITDVKFK TFGCGAAIAT SSMATELIKG KSINDALKLT
657322_Fprausnitzii@YP_007799942         E---N----N --VIVDVKFL TFGCGAAIAT SSMATDLIKG KTVDEALKLT
213810_Rchampanellensi@YP_007828576      Q---N----G --VLTDVKFK TFGCGAAVAT SSMATELIKG KPIEEALQLT
657319_[siraeum@YP_007775950             E---D----N --TIKDVKFK TFGCGAAIAT SSIATEMIKG KPLSEALKLT
293826_Ametalliredigen@YP_001320267      D---N----D --MITDVKFK TFGCGSAIAT SSMATEMIQG KTVKEVLQLT
350688_Aoremlandii@YP_001513205          E---N----D --VIVDVKFK TFGCGSAIAT SSMATEMIKG KTVKEALQLT
546269_Falocis@YP_005054244              K---D----N --RIEDVKFK TYGCGSAIAS SSMATEMIKG KTIDEVLEVT
1511_[sticklandii@YP_003936518           E---D----N --VIQDVKFK TYGCGSAIAS SSMATEMIKG KTVQEALNLT
699034_Pdifficile@YP_006198328           D---G----D --VIKDVKFK TFGCGSAIAS SSMATEMIKG KTIKDALELT
1128398_[acidurici@YP_006788189          D---N----D --VITDVKFK TFGCGSAIAS SSMATEMIKG KTIKEALEFT
572544_Ipolytropus@YP_003967871          E---N----D --IITDVKFR TFGCASAIAT SSISTEMILN KNVSEALQLT
469604_Fnucleatum@YP_008477745           D---N----D --IITDVKFR TFGCASAIAS SSISTDMIIG KTVEDALKVT
580340_Tlienii@YP_004932350              E---N----D --VITDIKFE TFGCASAIAS SSMVTEMVKG KTLDEALKIT
891968_Amobile@YP_006444325              K---D----D --RIVDVKFE TFGCAAAIAT SSMVTEMVKG KTLDEALKVT
525903_Tacidaminovoran@YP_003316727      R---D----G --KIEDVRFQ TFGCAAAIAT SSMVTEMVKG KTIDEALNVT
697281_Maustraliensis@YP_004463371       D---G----D --IITDVKFQ TFGCAAAIAT SSMATELIKG KTLDEALAIT
309799_Dthermophilum@YP_002251521        E---N----G --VIVDVKFK TLGCGAAIAT SSIATEMIKG KTIEEALKIT
368407_Mmarisnigri@YP_001047821          E---G----D --RIVDARFQ TFGCAAAIAS SSMATRMILG MTIAEAWNLS
903814_Elimosum@YP_003961665             S---D----D -GIVEDASFK TFGCGAAIAT SSMATEMIKG KTLEEAAALK
931626_Awoodii@YP_005268656              N---D----D -GVIEDASFK TFGCGAAVAS SSMATDMIIG KTIEEAGKFK
410358_Mlabreanum@YP_001029708           E---N----D --IIVDAKFQ TFGCGAAIAS SSMATEMIKG KTLEEAWTLS
679926_Mpetrolearius@YP_003895855        R---D----D --IIEDVKFQ TFGCGAAIAS SSMATEMIKG KTVKEAWDLS
86416_Cpasteurianum@YP_007940944         E---D----N --KIEDVKFK TFGCGSAIAS SSMATELIKG KTIEEAWDLT
929506_Cbotulinum@YP_004396224           E---D----N --IIKDVKFK TFGCGSAIAS SSMATELVKG KTLEDAWKLT
386415_Cnovyi@YP_878355                  E---D----N --IIKDVKFK TFGCGSAIAS SSMATELVKG KTLEEAWKLT
748727_Cljungdahlii@YP_003779382         E---D----D --VIKDIKFK TFGCGSAIAS SSMATELIKG KTLEDAWELT
1341692_Cautoethanogenu@YP_008700753     E---D----D --VIKDIKFK TFGCGSAIAS SSMATELIKG KTLEDAWELT
747365_Tnarugense@YP_004437867           K---D----N --IIEDVKYE TYGCASAIAS SSIASELIKG KTLEEAWKIT
651822_Ffastidiosum@YP_007827223         N---D---RN --VIEDVKFK TFGCASAIAS SSMATELIKG KTIDEAWDMT
572547_Acolombiense@YP_003553035         E---N----D --HIVDIKFK TFGCASAIAS SSMATELVMG KTIEEAWNIT
212717_Ctetani@NP_781697                 E---D----N --IIKDVKFK TFGCGSAIAS SSMATELIKG KTLEEAWELS
431943_Ckluyveri@YP_001394709            E---D----N --IIKDIKFK TFGCGSAIAS SSMATEIIKG KTLEEAWSLT
573061_Ccellulovorans@YP_003843335       E---N----D --IIVDVKFK TFGCGSAIAS SSMATELIKG KTLDEAWQLS
508767_Cbotulinum@YP_001920515           E---D----N --IIKDVKFK TFGCGSAIAS SSMATEMIKG KTLDEAWELS
931276_Csaccharoperbut@YP_007454264      E---D----N --IIKDVKFK TFGCGSAIAS SSMATELIKG KTLDEAWELT
290402_Cbeijerinckii@YP_001308237        E---D----N --IIKDVKFK TFGCGSAIAS SSMATELIKG KTLDEAWELT
1345695_Csaccharobutyli@YP_008674363     E---D----N --IIKDVKFK TFGCGSAIAS SSMATELIKG KTLDEAWELT
269797_Mbarkeri@YP_305925                E---D----N --RIADVKFQ TFGCGAAIAS SSMATEMIKG KTLEEAWKLS
188937_Macetivorans@NP_617616            E---G----D --RIVDVKFQ TFGCGAAIAS SSMATELIKG KILKEAWELS
195103_Cperfringens@YP_696469            E---N----N --IVEDVKFE TYGCGSAIAS SSMATELIKG KTVEEAWELS
593750_Mformicica@YP_007249963           E---N----N --IITDAKFK TFGCGAAIAS SSMATELVKG KTLEEAWDVS
456442_Mboonei@YP_001405283              E---N----N --IVVDAKFK TFGCGAAIAS SSMATELVRG KTLEEAWQLS
521011_Mpalustris@YP_002466865           R---E----N --RIEDVKFQ TFGCGAAVAS SSIATELIKG KTLDEAWETT
323259_Mhungatei@YP_503638               K---D----N --IITDVKFQ TFGCGAAVAS SSMATELIKG KTLEEAWEVS
547558_Mmahii@YP_003541558               E---D----N --VIADAKFM TFGCGAAIAS SSMATELIKG KTLEQAWKVT
644295_Mevestigatum@YP_003727500         E---D----N --IIQDAKFM TFGCGAAIAS SSMATELIKG KTLEEAWDVS
351627_Csaccharolyticu@YP_001181022      E---N----G --IIVDAKFK TFGCGAAVAT SSMATEMVKG KTIEEALQIT
521460_Cbescii@YP_002573580              E---N----G --IIVDAKFK TFGCGAAVAT SSMATEMVKG KTIEEALQIT
632292_Chydrothermalis@YP_003992133      E---N----G --IIVDAKFK TFGCGAAVAT SSMATEMVKG KTIEEALQIT
632348_Ckronotskyensis@YP_004023692      E---N----G --IIVDAKFK TFGCGAAVAT SSMATEMVKG KTIEEALQIT
632335_Ckristjanssonii@YP_004026829      E---N----G --IIVDAKFK TFGCGAAVAT SSMATEMVKG KTIEEALQIT
632516_Clactoaceticus@YP_004798736       E---N----G --IIVDAKFK TFGCGAAVAT SSMATEMVKG KTIEEALQIT
608506_Cobsidiansis@YP_003840166         E---N----G --IIVDAKFK TFGCGAAVAT SSMATEMVKG KTIEEALQIT
632518_Cowensensis@YP_004002802          E---N----G --IIVDAKFK TFGCGAAVAT SSIATEMVKG KTIEEALQIT
1121335_[stercorarium@YP_007372524       E---N----N --VIVDAKFK TFGCGAAVAT SSIATELIKG KTIDEALKVT
1121335_[stercorarium@YP_007678972       E---N----N --VIVDAKFK TFGCGAAVAT SSIATELIKG KTIDEALKVT
755731_Csp@YP_005148252                  E---D----N --IIVDAKFK TFGCGAAVAT SSMATELVKG KTVEEAMKIT
394503_Ccellulolyticum@YP_002506259      E---D----N --IVVDAKFK TFGCGAAVAT SSMATELVKG KTVEEAMNIT
720554_[clariflavum@YP_005046370         E---N----D --IIKDAKFK TFGCGAAVAT SSMATELVIG KTIDEALKIT
```

```
203119_Rthermocellum@YP_001037149        E---N----D --VIVDAKFK TFGCGAAVAT SSMATELVKG KTIEEALKIT
642492_Clentocellum@YP_004309737         E---D----G --IIKDAKFK TFGCGAAVAT SSMATELVIG KTIEEALKIT
1384484_Aequolifaciens@YP_008663926      D---E---NN --VIQDAKFK TFGCGAAIAT SSMATVLVKG KTVQEALKVT
515619_Erectale@YP_002936246             D---D---NG --IIQEAKFK TFGCGAAVAT SSMATELVKG KTIQEALEVT
718255_Rintestinalis@YP_007780168        D---D---NG --IIQEAKFK TFGCGAAVAT SSMATELVKG KTIQEALEVT
657315_Rintestinalis@YP_007830593        D---D---NG --IIQEAKFK TFGCGAAVAT SSMATELVKG KTIQEALEVT
357809_Lphytofermentan@YP_001560003      D---D---NQ --VIQDVKFK TFGCGAAVAT SSMATELVKG LTVQEALTVT
610130_[saccharolyticu@YP_003823446      D---D---NQ --IIRDVKFK TFGCGAAVAT SSMATEMVKG KSVQEAMAVT
717608_[cf@YP_007849106                  D---E---NQ --IIRDVKFK TFGCGAAVAT SSMATELVKG KSIKEAMQVT
245012_bproducing@YP_007810477           D---E---NQ --IIRDVKFK TFGCGAAVAT SSMATELVKG KSIKEAMQVT
657313_Rtorques@YP_007786815             D---E---NQ --IIRDVKFK TFGCGAAVAT SSMATEMVKG KSIQEAMEVT
245018_bproducing@YP_007788847           D---D---NG --IIQDVKFK TFGCGAAVAT SSMATELVKG KNIQQALEVT
585394_Rhominis@YP_004837975             D---E---NQ --VIRDCKFK TFGCGAAVAT SSMATELVKG KTIYEALQIT
1054217_Tarchaeon@YP_007688600           D---E---NG --IIRDCKFK TFGCGAAIAS SSMATEMVKG KSIHEALEIT
657323_Rsp@YP_007784833                  D---D--ETH --IIKDCKFK TFGCGAAVAT SSMATELVMG KTIEEAMEVT
657314_Robeum@YP_007804234               D---D--ETH --IIKECKFK TFGCGAAVAT SSMATEMVKG KTIEEAMQIT
717962_Ccatus@YP_007770008               D---E---NQ --VIRDVKFK TFGCGAAVAT SSMATELVKG KTIYEALEVT
552811_Dlykanthroporep@YP_003758020      K---D----G --IITDSSFK TFGCGAAIAT SSMVTEMVKG KTIKEALEIS
246194_Chydrogenoforma@YP_361011         D---G----D --KITDVKFK TFGCGAAIAT SSMVTEMVKG KTIEEAMKIT
696281_Druminis@YP_004546667             E---D----N --IIKDIKFK TFGCGAAVAT SSMVTEMAKG KTLEEALQIT
868595_Dcarboxydivoran@YP_004497987      E---D----N --VIKDIKFK TFGCGAAVAT SSMVTEMAMG KTIDEALKIS
349161_Dreducens@YP_001112128            E---D----N --IIKDIKFK TFGCGAAVAT SSMVTEMAMG KTIDEALTIT
634498_Mruminantium@YP_003424309         D---D----D -EVIQDIKFK TFGCGAAIAT SSMITEIAKG MAVDEALKIT
406327_Mvannielii@YP_001322697           E---D----E --IIKDIKFK TFGCGAAIAT SSMITELSKG KPINEALKIT
679901_Mzhilinae@YP_004616912            K---D----D --ILEDVKFK TFGCGAAIAT SSMITEMAKG KTIEEALRIT
224719_Msp@YP_008075510                  D---D---ND --VIRDVKFK TFGCGAAIAT SSMITELAIG KTVDEAYNIT
339860_Mstadtmanae@YP_448034             D---D---DN --KLSDVKFS TFGCGAAIAT SSMITEMAIG MDVDEAYHIT
1295009_CMethanomassili@YP_008071572     K---D----N --TITDVSFQ TLGCGAAIAS GSMITQMAKG MNIDDALNIQ
420247_Msmithii@YP_001272836             K---D----D --VITDISFQ TFGCGAAIAT SSMITEIAIG KTLEEALKIS
1041930_Mconradii@YP_005380864           K---D----N --VITDIKFK TFGCAAAIAT SSIITTMAKG MTLEDAEKIG
304371_Mpaludicola@YP_003355464          K---D----N --KLSDIKFK TFGCAAAIAT SSIITTMAKG MTLEDAVKIS
1379702_Msp@YP_008915990                 E---D----D --VITDIKFK TFGCGAAIAT SSMITEMAIG KTLDEALKIT
351160_Marvoryzae@YP_686139              K---D----D --RIEDIKFK TFGCGAAIAT SSMVTVMAKG KTLDEALKIT
990316_Mconcilii@YP_004384883            K---D----D --RIDDIKFR TFGCGSAIAV SSMVTEMAKG KILDEAIKIT
521011_Mpalustris@YP_002466708           E---K----D --VITDIKFK TFGCGSAIAT ASMVTELAKG KTLDEAEKIS
1201294_Mbourgensis@YP_006544991         R---E----N --VIEDIRFR TFGCGSAIAT SSMVTDLAKG KTLEEALQIT
368407_Mmarisnigri@YP_001048051          K---D----N --VIEDIRFR TFGCGSAIAT SSMVTDLAKG KTLDEAMRLT
635013_Tpotens@YP_003640662              E---D----N --IIKDIKFK TFGCGAAIAT SSMVTEMVKG KTIDEALEIT
457570_Nthermophilus@YP_001916489        N---N----N -EEIEDIKFK TFGCGAAIAT SSIVTEMAKG KHINEARDIK
574087_Aarabaticum@YP_003827335          E---D----D --VITDIKFK TFGCGAAIAT SSITTEMVKG KTLDEAEELT
748449_Hhalobius@YP_007315822            N---D----G --KINDIKFK TFGCGAAIAT SSIATELVKG KSIDEAKELS
338963_Pcarbinolicus@YP_006717600        E---N----D --IIEDVKFQ TYGCGAAIAS SSMMSELVIG KTLQQALDLT
498761_Hmodesticaldum@YP_001680434       E---D----N --IIKDVKFK TFGCGAAIAT SSMVTELIKG KTIDEALGLT
768704_Dmeridiei@YP_006620851            E---G----D --IIKDVKFK TFGCGAAVAT SSMVTEMVKG KTIDEAMEIS
768706_Dorientis@YP_004969159            E---G----D --IIKDVKFK TFGCGAAVAT SSMVTEMVKG KTIDEALEIS
1147129_Dsp@YP_006909410                 E---N----D --IIKDIKFK TFGCGAAVAT SSMVTEMVKG KSVNEALDIS
138119_Dhafniense@YP_518658              E---G----D --IIKDVKFK TFGCGAAVAT SSMVTEMVKG KSIDEALQIT
645991_Sglycolicus@YP_004266248          E---D----D --IIKDVKFK TFGCGAAVAT SSMVTEMVKG KTVEEAMNVT
646529_Dacidiphilus@YP_006467680         E---G----D --IIKDVKFK TFGCGAAVAT SSMVTEMVKG KTLEEALEIS
272564_Dhafniense@YP_002460033           E---G----D --IIKDVKFK TFGCGAAVAT SSMVTEMVKG KTIEEALKIT
756499_Ddehalogenans@YP_006431093        D---G----D --IIKDVKFK TFGCGAAVAT SSMVTEMVKG KTIEEALKIT
871963_Ddichloroelimin@YP_007221311      E---G----D --IIKDVKFK TFGCGAAVAT SSMVTEMVKG KTIEEALQIT
767817_Dgibsoniae@YP_007945765           D---N----E --VITDIKFK TFGCGAAIAT SSMVTEMAKG KTIEEALQLS
485916_Dacetoxidans@YP_003191710         E---D----N --IIKDIKFK TFGCGAAVAT SSMVTELAKG KTLEEALELS
555079_Toceani@YP_003825515              K---D----D --VIEDIKFK TFGCGAAIAT SSMVTEMVKG KTIEEALKVS
349307_Mthermophila@YP_843171            G---D----E --KIEDIKFR TFGCGAAIAT SSMITEMARG KSLEEAMRIT
760568_Dkuznetsovii@YP_004517387         K---D----N --IIEDIKFK TFGCGAAIAT SSMVTELVKG KTLEEAMQVS
373903_Horenii@YP_002508640              K---D----D --TIEDIKFK TFGCGAAVAT SSMVTELVKG KKIDEALKVS
370438_Pthermopropioni@YP_001211605      E---D----N --VIKDIKFK TFGCGAAIAS GSMLTEMAKG KTIEEALQIT
264732_Mthermoacetica@YP_430496          E---D----G --IIKDVKFK TFGCGAAIAT SSMVTEMVKG KTVEEALKIS
96561_Doleovorans@YP_001528642           Q---D----D --RIADIKFQ TFGCGSAIAV SSMLTELAKG KTLEEAKKIT
471821_uTermite@YP_001956213             K---D----N --KIEDVKFK TFGCGAAIAV SSMVSEMVLG KTLEEVMNLT
429009_Adegensii@YP_003238425            K---E----D --RIAEIKFQ TFGCAAAIAV ASMISEMAKG KHITEALKIT
706587_Dtiedjei@YP_006449617             N---N----N --VLEDVKFK TFGCGAAIAV SSMVSEMAKG KTLDEAMQIS
589924_Fplacidus@YP_003435557            K---D----D --KIVDIKFQ TFGCAAAIAT SSMATELAKG KTLEEALKIT
224325_Afulgidus@NP_069024               K---D----N --RIEDIKFQ TFGCAAAIAT SSMATEMAKG KTIEEALKIT
224325_Afulgidus@NP_069399               K---D----N --RIEDIKFQ TFGCAAAIAT SSMATEMAKG KTIEEALKIT
416591_Tlettingae@YP_001470347           E---D----D --RITDIKFE SYGCAANIAT ASMMTEVVKG LTLEEAKKLN
688269_Tthermarum@YP_004660083           E---N----D --RIVDIKFE SYGCAANIAT ASMMTEVVKG MTIEEAKKIT
374847_CKorarchaeum@YP_001737319         R---D----G --VIEDAKFE SYGCAANIAA ASVLTEIVKG KRLEEAWNID
583356_Iaggregans@YP_003860035           E---RIDNID --VIKEATFE SYGCAANIAA ASIVTEMIKG KDLRYAWNIT
272947_Rprowazekii@NP_220862             G-D-D----E --IITDAKFK TFGCGSAIAS SSLVTEWIKG KSVEDAKEIK
1282356_Ppoae@YP_007398800               N-D-A----G --VIEDAKFK TYGCGSAIAS SSLATEWMKG KTLDEAVTIS
994484_Pbrassicacearum@YP_004352099      N-E-Q----G --IIEDAKFK TYGCGSAIAS SSLATEWMKG KTLDEAETIK
882944_Languillarum@YP_008488748         T-P-E----G --IIEDAKFK TYGCGSAIAS SSLVTEWVKG KSIDEAAAIK
1167634_Axylosoxidans@YP_008032542       N-E-A----G --VIEDARFK TYGCGSAIAS SSLVTEWVKG KTLDEAMNIR
257311_Bparapertussis@NP_884287          N-E-A----G --VIEDARFK TYGCGSAIAS SSLVTEWVKG KTLDEAMNIR
1017264_Bpertussis@YP_005589900          N-E-A----G --VIEDARFK TYGCGSAIAS SSLVTEWVKG KTLDEAMNIR
284812_Spombe@NP_592967                  N-K-D----G --VIEDVKFK TFGCGSAIAS SSYVTTMVKG MTLEEASKIK
559292_Scerevisiae@NP_014869             N-DKS----G --IIENVKFK TFGCGSAIAS SSYMTELVRG MSLDEAVKIK
559292_Scerevisiae@NP_015190             N-DST----G --VIEDVKFK TFGCGSAIAS SSYMTELVQG MTLDDAAKIK
9606_Hsapiens@NP_998760                  D-E-K----G --KIVDARFK TFGCGSAIAS SSLATEWVKG KTVEEALTIK
9606_Hsapiens@NP_055116                  D-E-K----G --KIVDARFK TFGCGSAIAS SSLATEWVKG KTVEEALTIK
3702_Athaliana@NP_193953                 D-EKT----G --QIVDARFK TFGCGSAIAS SSVATEWVKG KAMEDVLTIK
3702_Athaliana@NP_192317                 D-G-S----G --QIIDAKFK TFGCGSAIAA SSVATEWVKG KSVEEVLTIK
3702_Athaliana@NP_186751                 D-D-S----G --QIIDTRFK TFGCGSAIAS SSVASEWIKG KTLDEVMTIK
7227_Dmelanogaster@NP_649840             D-E-N----G --KIVDAKFK TFGCGSAIAS SSLATEWVKG KSIDEAGKLK
6239_Celegans@NP_502658                  D-D-N----G --KIIEAKFK TFGCGSAIAS SSLATEWING KTIDYASKIK
```

$_{99}$LPPVK$_{103}$ X_{108}

```
                                        151          -------     *
243233_Mcapsulatus@YP_112782            NSDIAEAL-A LPPVKIHCSV LAEDAIKAAV SDYKAKLGVA DD-GYEIRKS
458817_Shalifaxensis@YP_001673798       NTDIAEEL-A LPPVKIHCSI LAEDAIKAAL EEYKTKQSK- ----------
398579_Spealeana@YP_001501349           NTDIAEEL-A LPPVKIHCSI LAEDAIKAAL DEYKTKQSK- ----------
557598_Lhongkongensis@YP_002796852      NTAIAEEL-A LPPVKIHCSI LAEDAIKAAV KDYREKHGSQ E---------
521006_Ngonorrhoeae@YP_002001906        NSEIAEEL-E LPPVKIHCSI LAEDAVKAAV ADYRKRQENR ----------
489653_Nlactamica@YP_004048810          NSEIAEEL-E LPPVKIHCSI LAEDAVKAAV ADYRKRQENR ----------
935589_Nmeningitidis@YP_005903962       NSEIAEEL-E LPPVKIHCSI LAEDAVKAAV ADYRKRQENR ----------
395495_Lcholodnii@YP_001790394          NTAIAEEL-A LPPVKIHCSI LAEDAIKAAV ADYRHRHDAH IE-QPACSPA
999541_Bgladioli@YP_004361365           NTQIAEEL-A LPPVKIHCSI LAEDAIKAAV ADYRKRHDAT AE-DGQAA--
983917_Rgelatinosus@YP_005439140        NTAIAEEL-A LPPVKIHCSI LAEDAIKAAV ADYRQRHAGP GG-LIEERAC
1416914_Ppnomenusa@YP_008837319         NTEIAQEL-A LPPVKIHCSI LAEDAIKAAV ADYKQKHGQT AD-AA-----
1380774_Psp@YP_008881415                NTEIAQEL-A LPPVKIHCSI LAEDAIKAAV ADYKQKHGQT AD-AA-----
1249661_Bthailandensis@YP_007917844     NTQIAEEL-A LPPVKIHCSI LAEDAIKAAV ADYKKRHEMA ED-GKAAA--
320388_Bmallei@YP_993528                NTQIAEEL-A LPPVKIHCSI LAEDAIKAAV ADYKKRHETA ED-GKAAA--
536230_Bpseudomallei@YP_002897469       NTQIAEEL-A LPPVKIHCSI LAEDAIKAAV ADYKKRHETA ED-GKAAA--
395019_Bmultivorans@YP_001579332        NTQIAEEL-A LPPVKIHCSI LAEDAIKAAV ADYKKRHDTK QD-DQAAA--
626418_Bglumae@YP_002912250             NTQIAEEL-A LPPVKIHCSI LAEDAIKAAV ADYRKRHDAT AE-DGQAA--
365044_Pnaphthalenivor@YP_981803        NSDIAEEL-A LPPVKIHCSI LAEDAIKAAV ADFRARHPAG SG-AATILTE
266265_Bxenovorans@YP_553871            NTQIAEEL-A LPPVKIHCSI LAEDAIKAAV ADFRSRQAIQ GT-AADSLQL
269482_Bvietnamiensis@YP_001115172      NTDIAEEL-A LPPVKIHCSI LAEDAIKAAV ADFRSRHPGA GG-ETLAEQP
583345_Mmobilis@YP_003049105            NSEIAEEL-A LPPVKIHCSV LAEDAIKAAV ADIKAKQAAK EN-A------
666681_Mversatilis@YP_003674921         NSEIAEEL-A LPPVKIHCSV LAEDAIKAAV ADIKAKQAAK VT-A------
391038_Bphymatum@YP_001857685           NTQIAEEL-A LPPVKIHCSI LAEDAIKAAV ADYKQRHGET AK-AGQSA--
398577_Bambifaria@YP_001808732          NTQIAEEL-A LPPVKIHCSI LAEDAIKAAV ADYKKRHDTT EG-DQAAA--
269482_Bvietnamiensis@YP_001120041      NTQIAEEL-A LPPVKIHCSI LAEDAIKAAV ADYKKRHDTK EG-DQAAA--
406425_Bcenocepacia@YP_001765425        NTQIAEEL-A LPPVKIHCSI LAEDAIKAAV ADYKKRHDTK ES-DQAAA--
1009846_Bcepacia@YP_006615433           NTQIAEEL-A LPPVKIHCSI LAEDAIKAAV ADYKKRHDTT EG-DQAAA--
482957_Blata@YP_369669                  NTQIAEEL-A LPPVKIHCSI LAEDAIKAAV ADYKKRHDTT EG-DQAAA--
748280_Psp@YP_004848592                 NTEIAEEL-A LPPVKIHCSI LAEDAIKAAV SDYKQKHGK- ----------
1229205_Bphenoliruptrix@YP_006834626    NTQIAEEL-A LPPVKIHCSI LAEDAIKAAV ADYKQRHGEA VA-ESDKQHA
1163617_Sdenitrificans@YP_008546117     NTQIAEEL-A LPPVKIHCSV LAEDAIKAAV ADYKSKHGAM VE-TAACTGS
266265_Bxenovorans@YP_559453            NTQIAEEL-A LPPVKIHCSI LAEDAIKAAV ADYKQRHGEA VV-ADEKQHA
398527_Bphytofirmans@YP_001896197       NTQIAEEL-A LPPVKIHCSI LAEDAIKAAV ADYKQRHGEA ASVEGDKQHA
375286_Jsp@YP_001352943                 NTQIAEEL-A LPPVKIHCSI LAEDAIKAAV LDYKTKHGVA ES-KEAA---
204773_Harsenicoxydans@YP_001100494     NTQIAEEL-A LPPVKIHCSI LAEDAIKAAV LDYKTKHGVA EA-EKAV---
580332_Slithotrophicus@YP_003524869     NTHIAEEL-A LPPVKIHCSI LAEDAIKAAV ADYKAKNGAT LE-TAACNGN
159087_Daromatica@YP_285167             NTEIAEEL-A LPPVKIHCSI LAEDAIKAAV ADYKKKHGE- ----------
882378_Brhizoxinica@YP_004029636        NSQIAEEL-A LPPVKIHCSI LAEDAIKAAV ADYKKRRVAV AG-EPAVQAG
85643_Tsp@YP_002355840                  NTQIAEEL-A LPPVKIHCSI LAEDAIKAAV ADYKKKHEA- ----------
757424_Hseropedicae@YP_003776230        NTAIADEL-A LPPVKIHCSI LAEDAIKAAV ADYRSRHDAA PE-VQQAAPA
522306_CAccumulibacter@YP_003167247     NTQIADEL-A LPPVKIHCSI LAEDAIKAAV ADYRKKHAES V---------
76114_Aaromaticum@YP_160687             NTQIAEEL-A LPPVKIHCSI LAEDAIKAAV ADYKKKQG-- ----------
381666_Reutropha@YP_725668              NTQIAEEL-A LPPVKIHCSI LAEDAIKAAV EDYKKKHGAE QK-AA-----
977880_Ctaiwanensis@YP_002005171        NTQIAEEL-A LPPVKIHCSI LAEDAIKAAV EDYKKKHGAE QK-AA-----
1042878_Cnecator@YP_004684939           NTQIAEEL-A LPPVKIHCSI LAEDAIKAAV EDYKKKHGAE QK-AA-----
266264_Cmetallidurans@YP_583181         NTQIAEEL-A LPPVKIHCSI LAEDAIKAAV DDYKKKHPGA EQ-KAA----
395494_Gcapsiferriform@YP_003846450     NTQIAEEL-A LPPVKIHCSI LAEDAIKAAV ADYKSKHGEN VE-TSACTGS
859655_Rsolanacearum@YP_005996961       NTQIAEEL-A LPPVKIHCSI LAEDAIKAAV ADYKEKHGTA EQ-KAA----
402626_Rpickettii@YP_001898467          NTAIAEEL-A LPPVKIHCSI LAEDAIKAAV ADYKEKHGSA EQ-KAA----
543913_bproteobacteriu@YP_007502653     NSLIAEEL-A LPPVKIHCSI LAEDAIKAAV ADYKEKHPAQ ----------
62928_Asp@YP_933519                     NTQIAEEL-A LPPVKIHCSI LAEDAIKAAV ADYKKKHEA- ----------
640081_Dsuillum@YP_005028573            NTDIAEEL-A LPPVKIHCSI LAEDAIKAAV ADYKKKHGDN ----------
452638_Pnecessarius@YP_001797351        NSLIAEEL-A LPPVKIHCSI LAEDAIKAAV ADYKEKHPAK ----------
292415_Tdenitrificans@YP_314923         NTAIAEEL-A LPPVKIHCSI LAEDAIKAAV ADYKQKKGLE ----------
265072_Mflagellatus@YP_544916           NSDIAQEL-A LPPVKIHCSV LAEDAIRAAV ADLKAKQGAK DQ-QVA----
582744_Mglucosetrophus@YP_003051619     NSDIAEEL-A LPPVKIHCSV LAEDAIRAAV ADLKAKQGNT QE-AA-----
887061_Msp@YP_004040241                 NSDIAEEL-A LPPVKIHCSV LAEDAIRAAV ADLKAKQGNT QE-AA-----
891398_CTremblaya@YP_004707015          NSAIAQEL-G LPPVKIHCSI LAEEALRAAV ADYRSKTSQQ CG-G------
426114_Tarsenitoxydans@YP_003623866     NSQIAEEL-A LPPVKIHCSI LAEDAIKAAV SDYQQRHGRT AG-QALATEQ
75379_Tintermedia@YP_003642782          NSQIAEEL-A LPPVKIHCSI LAEDAIKAAV SDYQQRHGRN AG-QALATEQ
937774_Tequigenitalis@YP_004130301      NTDIAEEL-A LPPVKIHCSI LAEDAIKAAI SDYRKKKEGK V---------
1008459_Tasinigenitalis@YP_004874474    NTDIAEEL-A LPPVKIHCSI LAEDAIKAAI NDYRHKKEGK S---------
634503_Eictaluri@YP_002934567           NTDIAEEL-A LPPVKIHCSI LAEDAIKAAI ADYKNKHSAE ----------
718251_Etarda@YP_005700147              NTDIAEEL-A LPPVKIHCSI LAEDAIKAAI ADYKNKHSAE ----------
259536_Parcticus@YP_264760              NSDIAEEL-A LPPVKVHCSV LAEDAIKAAI SDYKGKHIPA ----------
335284_Pcryohalolentis@YP_580918        NSHIAEEL-A LPPVKVHCSV LAEDAIKAAI SDYKGKHVPA ----------
571800_Psp@YP_008163576                 NSHIAEEL-A LPPVKVHCSV LAEDAIKAAI SDYKGKHVPA ----------
420662_Mpetroleiphilum@YP_001021452     NTQIAEEL-A LPPVKIHCSI LAEDAIKAAV SDYKAKHGNA QP-AAATATH
243365_Cviolaceum@NP_900763             NTAIAEEL-A LPPVKIHCSI LAEDAIKAAV EDYKQKHGK- ----------
1208919_CKinetoplastiba@YP_007459239    NVDIAEEL-A LPPVKIHCSI LAEDAIKSAI EDYRIKNKNN TI--------
983545_Gsp@YP_004435367                 NTDIAEEL-A LPPVKIHCSI LAEDAIKAAV EDYKKKHL-- ----------
342610_Patlantica@YP_660819             NMDIAEEL-A LPPVKIHCSI LAEDAIKAAV EDYKKKHQ-- ----------
743299_Aferrivorans@YP_004783591        NSHIAEEL-E LPPVKIHCSV LAEDAIKAAV EDYRNKQGGA QV-AAPAEMA
243159_Aferrooxidans@YP_002425162       NSQIAEEL-E LPPVKIHCSV LAEDAIKAAV EDYRKKQGGA ST-AAQPEMA
1005048_Cfungivorans@YP_004753777       NTQIAEEL-A LPPVKIHCSI LAEDAIKAAV EDYKAKHGEQ KQ-AA-----
669502_CProfftella@YP_008343646         NTDIAEEL-V LPPLKIHCSI LAEDAIKLAI ESYKDKQKNI KN-K------
757424_Hseropedicae@YP_003776535        NTQIAEEL-A LPPVKIHCSI LAEDAIKAAV QDYKAKHGAE QK-AA-----
395495_Lcholodnii@YP_001790080          NTHIAEEL-A LPPVKIHCSI LAEDAIKAAV DDYKAKHAQ- ----------
257310_Bbronchiseptica@NP_888820        NTQIAEEL-A LPPVKIHCSI LAEDAIKAAV QDYKTKHGAE TP-EAVAAN-
990288_Acaldus@YP_004748177             NSQIAEEL-E LPPVKIHCSV LAEDAIKAAV QDYLQKHGSA AG-EAKVAAE
990288_Acaldus@YP_004748110             NRQIAEEL-E LPPVKIHCSV LAEDAIKAAV QDYRRKHGKG SG-SEAD---
983917_Rgelatinosus@YP_005436383        NTDIAEEL-A LPPVKIHCSI LAEDAIKAAV QDYKAKHGEA VA-TQH----
360910_Bavium@YP_786035                 NTQIAEEL-A LPPVKIHCSI LAEDAIKAAV QDYKTKHGAA EA-VTAD---
1036672_Akashmirensis@YP_006379415      NKQIAEEL-A LPPVKIHCSI LAEDAIKAAV QDYKDKHTG- ----------
340100_Bpetrii@YP_001631386             NTQIAEEL-A LPPVKIHCSI LAEDAIKAAV KDYKDKHAAP AD-AQGETAA
523791_Kkoreensis@YP_003146685          NTQIAEEL-A LPPVKIHCSV LAEDAIKAAV KDYKEKHGQ- ----------
156889_Mmarinus@YP_866958               NKDVAEEL-A LPPVKVHCSV LAEDAIKAAV KDYREKQQKK GA--------
```

```
                                                          99LPPVK103  X108
                                                          -------     *
404589_Asp@YP_001377835                NTDVANEL-N LPPVKIHCSV LAEDAIKAAV ADWQKKRGVA PT-VKPDTVQ
290397_Adehalogenans@YP_463806         NTDVANEL-N LPPVKIHCSV LAEDAIKAAV ADWQKKHGRE PS-VKPNTVQ
447217_Asp@YP_002132996                NTDVANEL-N LPPVKIHCSV LAEDAIKAAV ADWQKKHGRE PS-VKPNTVQ
455488_Adehalogenans@YP_002491042      NTDVANEL-N LPPVKIHCSV LAEDAIKAAV ADWQKKHGRE PS-VKPNTVQ
378806_Saurantiaca@YP_003955403        NKDVAREL-S LPPVKIHCSV LAEDAIKAAI EDFKKKRQGR AP-KQTA---
246197_Mxanthus@YP_633158              NKDVAREL-S LPPVKIHCSV LAEDAIKAAI EDFKKKRAAR QA-KAS----
483219_Mfulvus@YP_004669542            NKDVAREL-S LPPVKIHCSV LAEDAIKAAI EDFKKKRAAR QA-KAS----
1144275_Ccoralloides@YP_005368444      NKDVAREL-A LPPVKIHCSV LAEDAIKAAI EDFKKKRASR QA--------
1278073_Mstipitatus@YP_007362455       NKDVAREL-A LPPVKIHCSV LAEDAIKAAI EDFKKKRAAR KA-QAS----
448385_Scellulosum@YP_001617979        NSMIAEEL-H LPPVKIHCSV LAEDAIKSAI ADFRAKQQAK RE-LAGAAPS
399739_Pmendocina@YP_001188990         NTQLAEEL-A LPPVKIHCSV LAEDAIKAAV RDYKQKKGLL ----------
1294143_Pdenitrificans@YP_007659193    NTTIAEEL-A LPPVKIHCSV LAEDAIKAAV RDYKQKKGLL ----------
208964_Paeruginosa@NP_252502           NTTIAEEL-A LPPVKIHCSV LAEDAIKAAV RDYKQKKGLL ----------
322710_Avinelandii@YP_002801150        NTQIAEEL-A LPPVKIHCSV LAEDAIKAAV RDYKHKKGLV ----------
644801_Pstutzeri@YP_007239445          NTQLAEEL-A LPPVKIHCSV LAEDAIKAAV RDYREKKGLL ----------
1207075_Psp@YP_007027941               NTQLAEEL-A LPPVKIHCSV LAEDAIKAAV RDYKQKKGLI ----------
1114970_Pfluorescens@YP_005206437      NTQLAEEL-A LPPVKIHCSV LAEDAIKAAV RDYKQKKGLI ----------
220664_Pprotegens@YP_262044            NTQLAEEL-A LPPVKIHCSV LAEDAIKAAV RDYKQKKGLI ----------
351746_Pputida@YP_001266219            NTQLAEEL-A LPPVKIHCSV LAEDAIKAAV RDYKQKKGLI ----------
1435058_Pmonteilii@YP_008958359        NTQLAEEL-A LPPVKIHCSV LAEDAIKAAV RDYKQKKGLI ----------
743720_Pfulva@YP_004475358             NTQLAEEL-A LPPVKIHCSV LAEDAIKAAV RDYKQKKGLL ----------
264730_Psyringae@YP_273568             NTQLAEEL-A LPPVKIHCSV LAEDAIKAAV RDYKQKKGLL ----------
384676_Pentomophila@YP_606728          NTQLAEEL-A LPPVKIHCSV LAEDAIKAAV RDYKQKKGLI ----------
1430440_Mgryphiswaldens@YP_008937260   NTDIANEL-A LPPVKIHCSV LAEDAIKAAI KDYKSKSGQT ED-EKAAG--
749219_Mcatarrhalis@YP_003626770       NKDIANEL-A LPPVKVHCSV LAEDAIKAAI EDYQTKAQA- ----------
62977_Asp@YP_046090                    NIDIANEL-A LPPVKVHCSV LAEDAIKAAI EDYRNKRSKA ----------
436717_Aoleivorans@YP_003732380        NIDIATEL-A LPPVKVHCSV LAEDAIKAAI EDYRTKKSKA ----------
400667_Abaumannii@YP_001084660         NIDIATEL-A LPPVKVHCSV LAEDAIKAAI EDYRSKKSKA ----------
871585_Acalcoaceticus@YP_004995269     NIDIATEL-A LPPVKVHCSV LAEDAIKAAI DDYRSKKSKA ----------
511062_Osp@YP_005091814                NTDIAEEL-A LPPVKIHCSI LAEDAIKAAI DDYKQKKNLA ----------
318167_Sfrigidimarina@YP_751106        NADIAEEL-A LPPVKIHCSI LAEDAIKAAI EEYKSRQAK- ----------
326297_Samazonensis@YP_927171          NTDIAEEL-A LPPVKIHCSI LAEDAIKAAL EEYKHKQAK- ----------
318161_Sdenitrificans@YP_562467        NADIAQEL-A LPPVKIHCSI LAEDAIKAAL DEYKSRQVK- ----------
167879_Cpsychrerythrae@YP_267876       NTAIAEEL-A LPPVKIHCSI LAEDAIKAAI EDYKSKQSK- ----------
637905_Sviolacea@YP_003556341          NTDIAEEL-A LPPVKIHCSI LAEDAIKAAI EDYKSKQAK- ----------
225849_Spiezotolerans@YP_002311055     NTDIAEEL-A LPPVKIHCSI LAEDAIKAAL DEYKTKQSK- ----------
323850_Sloihica@YP_001094441           NTDIAEEL-A LPPVKIHCSI LAEDAIKAAL DEYKAKHEK- ----------
425104_Ssediminis@YP_001474605         NTDIAEEL-A LPPVKIHCSI LAEDAIKAAI DDYKSKQTK- ----------
392500_Swoodyi@YP_001760156            NTDIAEEL-A LPPVKIHCSI LAEDAIKAAI DDYKSKQDK- ----------
60481_Ssp@YP_737869                    NTDIAEEL-A LPPVKIHCSI LAEDAIKAAI DEYKSKQAK- ----------
211586_Soneidensis@NP_717861           NTDIAEEL-A LPPVKIHCSI LAEDAIKAAI DEYKSKQAK- ----------
693973_Sbaltica@YP_005273706           NTDIAEEL-A LPPVKIHCSI LAEDAIKAAI DEYKSKQAK- ----------
399804_Sputrefaciens@YP_006009782      NTDIAEEL-A LPPVKIHCSI LAEDAIKAAI DEYKSKQAQ- ----------
298386_Pprofundum@YP_128966            NSAIAEEL-E LPPVKIHCSI LAEDAIKAAV SDYKKKHEAN ----------
28173_Vnigripulchritu@YP_008621944     NAEIAEEL-E LPPVKVHCSI LAEDAIKAAV SDYKKKHDS- ----------
1219076_Valginolyticus@YP_008537102    NSEIAEEL-E LPPVKVHCSI LAEDAIKAAV ADYKKKHQE- ----------
150340_Vsp@YP_003286982                NSEIAEEL-E LPPVKVHCSI LAEDAIKAAV ADYKKKHQE- ----------
345073_Vcholerae@YP_001216234          NSEIAEEL-E LPPVKIHCSI LAEDAIKAAV ADYKKKHQH- ----------
223926_Vparahaemolytic@NP_796976       NSEIAEEL-E LPPVKVHCSI LAEDAIKAAV ADYKKKHQE- ----------
882102_Vanguillarum@YP_004566935       NSEIAEEL-E LPPVKVHCSI LAEDAIKAAV ADYKKKHQQ- ----------
575788_Vsplendidus@YP_002416251        NSEIAEEL-E LPPVKVHCSI LAEDAIKAAV ADYKKKH--- ----------
1129794_Gpsychrophila@YP_007545568     NMDIAEEL-A LPPVKIHCSI LAEDAIKAAV ADYKSRQV-- ----------
196600_Vvulnificus@NP_933549           NSEIAEEL-E LPPVKVHCSI LAEDAIKAAV ADYKKKHQL- ----------
903510_Vfurnissii@YP_004993913         NSEIAEEL-E LPPVKVHCSI LAEDAIKAAV ADYKKKHEL- ----------
338187_Vcampbellii@YP_001444276        NSEIAEEL-E LPPVKVHCSI LAEDAIKAAV ADYKKKHQE- ----------
338187_Vcampbellii@YP_008526292        NSEIAEEL-E LPPVKVHCSI LAEDAIKAAV ADYKKKHQE- ----------
314282_Psp@YP_007639631                NTEITAEL-A LPPVKIHCSI LAEDAIKAAI ADYRSKNS-- ----------
357804_Pingrahamii@YP_942749           NTQISAEL-A LPPVKIHCSI LAEDAIKAAI TDYKSKHPNA ----------
326442_Phaloplanktis@YP_341160         NTDISAEL-E LPPVKIHCSI LAEDAIQAAI ANYKSKQAK- ----------
234831_Psp@YP_004067531                NTDISAEL-E LPPVKIHCSI LAEDAIQAAI ADYKSKHTN- ----------
595494_Tauensis@YP_002893203           NTDIAEEL-A LPPVKIHCSI LAEDAIKAAI ADYKHKKGLN ----------
396588_Tsulfidiphilus@YP_002513559     NTQIAEEL-A LPPVKIHCSV LAEDAIKAAI ADFKQKSGAE DE-RKIA---
396588_Tsulfidiphilus@YP_002514125     NTQIAEEL-A LPPVKIHCSV LAEDAIKAAI ADFKQKSGAE EP-AKKTA--
998088_Averonii@YP_004393031           NTDIAEEL-A LPPVKIHCSI LAEDAIKAAI ADYKQKKGL- ----------
380703_Ahydrophila@YP_856284           NTDIAEEL-A LPPVKIHCSI LAEDAIKAAI ADYKQKKGL- ----------
382245_Asalmonicida@YP_001142383       NTDIAEEL-A LPPVKIHCSI LAEDAIKAAI ADYKQKKGL- ----------
342108_Mmagneticum@YP_422390           NTDIAHEL-A LPPVKIHCSV LAEDAIKAAI ADYKKKSG-- ----------
1245471_Presinovorans@YP_008101472     NTTIAEEL-A LPPVKIHCSV LAEDAIKAAV HDYKQKKGLL ----------
365044_Pnaphthalenivor@YP_982518       NSAIAEEL-A LPPVKIHCSI LAEDAIKAAV NDYKQKHSIA EA-AH-----
296591_Psp@YP_549004                   NSAIAEEL-A LPPVKIHCSI LAEDAIKAAV NDYKQKHAH- ----------
232721_Asp@YP_986388                   NSQIAEEL-A LPPVKIHCSI LAEDAIKAAV NDYKAKHGAA VE-AA-----
535289_Aebreus@YP_002553097            NSQIAEEL-A LPPVKIHCSI LAEDAIKAAV NDYKAKHGAA VE-AA-----
365046_Rtataouinensis@YP_004618947     NSQIAEEL-A LPPVKIHCSI LAEDAIKAAV EDYKKKRTH- ----------
946483_CSymbiobacter@YP_008681130      NSDIAREL-A LPPVKVHCSI LAEDAIKAAV SDYRNKHGDT PA-QPDTP--
312309_Vfischeri@YP_204001             NAEIAEEL-E LPPVKVHCSI LAEDAIKAAV SDYRNKHQD- ----------
595537_Vparadoxus@YP_004156043         NAQIAEEL-A LPPVKIHCSI LAEDAIKAAV SDYKAKHGAK TA-ESAAEAV
316275_Asalmonicida@YP_002262235       NAEIAEEL-E LPPVKVHCSI LAEDAIKAAV LDYRNKH--- ----------
391735_Veiseniae@YP_997138             NSEIAQEL-A LPPVKIHCSI LAEDAIKAAV LDYKTKRMAA AA-VA-----
397945_Acitrulli@YP_970793             NAEIAQEL-A LPPVKIHCSI LAEDAIKAAV QDYKAKHSAL AG-A------
643561_Aavenae@YP_004235248            NAEIAQEL-A LPPVKIHCSI LAEDAIKAAV QDYKAKHSAL AG-A------
398578_Dacidovorans@YP_001565014       NSQIAEEL-A LPPVKVHCSI LAEDAIKAAV DDYRAKRTAV VA-A------
742013_Dsp@YP_004488168                NSQIAEEL-A LPPVKVHCSI LAEDAIKAAV DDYRAKRTAV VA-A------
338969_Aferrireducens@YP_523433        NSEIAEEL-A LPPVKIHCSI LAEDAIKAAV DDYRKKHVAA LT-N------
596154_Adenitrificans@YP_004388278     NSQIAEEL-A LPPVKIHCSI LAEDAIKAAV NDYKAKHEAA VE-AA-----
688245_Ctestosteroni@YP_003278774      NSQIAEEL-A LPPVKVHCSI LAEDAIKAAV NDYRAKRTAT EA--------
1266738_Pmirabilis@YP_008398502        NTAIAEEL-E LPPVKIHCSI LAEDAIKAAI ADYKSKRQGK ----------
1124991_Mmorganii@YP_007505117         NTAIAEEL-E LPPVKIHCSI LAEDAIKAAI ADYKSKRRGN ----------
406817_Xnematophila@YP_003713469       NTQIADEL-E LPPVKIHCSI LAEDAIKAAI ADYKSKRQAK ----------
```

$_{99}$LPPVK$_{103}$ X$_{108}$

```
                                                    -------    *
291112_Pasymbiotica@YP_003040200            NTEIADEL-E LPPVKIHCSI LAEDAIKAAI ADYKSKRQGK -----------
243265_Pluminescens@NP_930506               NTEIADEL-E LPPVKIHCSI LAEDAIKAAI ADYKSKRQGR -----------
406818_Xbovienii@YP_003468915               NTAIADEL-E LPPVKIHCSI LAEDAIKAAI ADYKSKREAK -----------
273123_Ypseudotubercul@YP_071365            NTQIAEEL-E LPPVKIHCSI LAEDAIKAAI ADYKSKHTAK -----------
393305_Yenterocolitica@YP_001005390         NTQIAEEL-E LPPVKIHCSI LAEDAIKAAI ADYKSKHTAK -----------
1346614_Sliquefaciens@YP_008231656          NTQIAEEL-E LPPVKIHCSI LAEDAIKAAI ADYKSKHNAK -----------
1348660_Splymuthica@YP_008160622            NTQIAEEL-E LPPVKIHCSI LAEDAIKAAI ADYKSKHSAK -----------
399741_Sproteamaculans@YP_001479850         NTQIAEEL-E LPPVKIHCSI LAEDAIKAAI ADYKSKHSAK -----------
1249634_Smarcescens@YP_007345780            NTEIAEEL-E LPPVKIHCSI LAEDAIKAAI ADYKSKHDAK -----------
187410_Ypestis@NP_668657                    NTQIAEEL-E LPPVKIHCSI LAEDAIKAAI ADYKSKHTAK -----------
1157951_Pstuartii@YP_006215182              NTAIAEEL-E LPPVKIHCSI LAEDAIKAAI ADYKSKRQGK -----------
344609_Sboydii@YP_001881320                 NTDIAEEL-E LPPVKIHCSI LAEDAIKAAI ADYKSKREAK -----------
104623_Ssp@YP_008524031                     NTQIAEEL-E LPPVKIHCSI LAEDAIKAAI ADYKSKHDAN -----------
561229_Dzeae@YP_003003456                   NTQIAEEL-E LPPVKIHCSI LAEDAIKAAI ADYKSKRGDK -----------
590409_Ddadantii@YP_003334610               NTQIAEEL-E LPPVKIHCSI LAEDAIKAAI ADYKSKRSDK -----------
561230_Pcarotovorum@YP_003018589            NTQIAEEL-E LPPVKIHCSI LAEDAIKAAI ADYKSKRDAQ -----------
561231_Pwasabiae@YP_003258666               NTQIAEEL-E LPPVKIHCSI LAEDAIKAAI ADYKSKRDAQ -----------
1166016_Psp@YP_006282128                    NTQIAEEL-E LPPVKIHCSI LAEDAIKAAI ADYKSKRDAQ -----------
218491_Patrosepticum@YP_051325              NTQIAEEL-E LPPVKIHCSI LAEDAIKAAI ADYKSKRDAQ -----------
99287_Senterica@NP_461477                   NTDIADEL-E LPPVKIHCSI LAEDAIKAAI ADYKSKREAK -----------
218493_Sbongori@YP_004731144                NTDIADEL-E LPPVKIHCSI LAEDAIKAAI ADYKSKREAK -----------
290339_Csakazakii@YP_001436836              NTDIADEL-E LPPVKIHCSI LAEDAIKAAI ADYKSKREAK -----------
693216_Cturicensis@YP_003211492             NTDIADEL-E LPPVKIHCSI LAEDAIKAAI ADYKSKREAK -----------
399742_Esp@YP_001177741                     NTDIADEL-E LPPVKIHCSI LAEDAIKAAI ADYKSKREAK -----------
640513_Easburiae@YP_004829762               NTDIADEL-E LPPVKIHCSI LAEDAIKAAI ADYKSKREAK -----------
693444_Ebacterium@YP_007339349              NTDIADEL-E LPPVKIHCSI LAEDAIKAAI ADYKSKREAK -----------
290338_Ckoseri@YP_001451852                 NTDIADEL-E LPPVKIHCSI LAEDAIKAAI ADYKSKREAK -----------
484021_Kpneumoniae@YP_002920722             NTDIADEL-E LPPVKIHCSI LAEDAIKAAI ADYKSKREAK -----------
637910_Crodentium@YP_003366009              NTDIADEL-E LPPVKIHCSI LAEDAIKAAI ADYKSKREAK -----------
640131_Kvariicola@YP_003438138              NTDIADEL-E LPPVKIHCSI LAEDAIKAAI ADYKSKREAK -----------
1191061_Koxytoca@YP_006500071               NTDIADEL-E LPPVKIHCSI LAEDAIKAAI ADYKSKREAK -----------
1286170_Rornithinolytic@YP_007872306        NTDIADEL-E LPPVKIHCSI LAEDAIKAAI ADYKSKREAK -----------
511145_Ecoli@NP_417024                      NTDIADEL-E LPPVKIHCSI LAEDAIKAAI ADYKSKREAK -----------
585054_Efergusonii@YP_002381825             NTDIAEEL-E LPPVKIHCSI LAEDAIKAAI ADYKSKREAK -----------
198214_Sflexneri@NP_708368                  NTDIAEEL-E LPPVKIHCSI LAEDAIKAAI ADYKSKREAK -----------
216599_Ssonnei@YP_005457544                 NTDIAEEL-E LPPVKIHCSI LAEDAIKAAI ADYKSKREAK -----------
300267_Sdysenteriae@YP_404261               NTDIAEEL-E LPPVKIHCSI LAEDAIKAAI ADYKSKREAK -----------
716541_Ecloacae@YP_003614361                NTDIAEEL-E LPPVKIHCSI LAEDAIKAAI ADYKSKREAK -----------
701347_Elignolyticus@YP_003940771           NTDIAEEL-E LPPVKIHCSI LAEDAIKAAI ADYKSKREAK -----------
630626_Sblattae@YP_006318650                NTDIAEEL-E LPPVKIHCSI LAEDAIKAAI ADYKSKREAK -----------
1028307_Eaerogenes@YP_004590373             NTDIAEEL-E LPPVKIHCSI LAEDAIKAAI ADYKSKREAK -----------
557723_Hparasuis@YP_002474707               NSDIAEEL-E LPPVKVHCSI LAEDAIKAAI ADYKEKNGK- -----------
1005058_Ganatis@YP_004420698                NSDIAAEL-E LPPVKVHCSI LAEDAIKAAI ADYKAKNSK- -----------
1075089_Pmultocida@YP_005176279             NSQIAEEL-E LPPVKVHCSI LAEDAIKAAI ADYKAKKEAK -----------
205914_Hsomnus@YP_718490                    NSQIAEEL-E LPPVKVHCSI LAEDAIKAAI ADYKAKKAE- -----------
1171377_Btrehalosi@YP_007548422             NSDIAEEL-E LPPVKVHCSI LAEDAIKAAI ADYKEKQGK- -----------
233412_Hducreyi@NP_873560                   NSDIAEEL-E LPPVKVHCSI LAEDAIKAAI ADYKEKQAK- -----------
416269_Apleuropneumoni@YP_001053631         NSDIAEEL-E LPPVKVHCSI LAEDAIKAAI ADYKEKQAK- -----------
696748_Asuis@YP_006816777                   NSDIAEEL-E LPPVKVHCSI LAEDAIKAAI ADYKEKQAK- -----------
1366053_Mhaemolytica@YP_008338465           NSDIAEEL-E LPPVKVHCSI LAEDAIKAAI ADYKEKQAK- -----------
1334187_Hinfluenzae@YP_008544790            NSQIAEEL-E LPPVKVHCSI LAEDAIKAAI ADYKAKQGE- -----------
634176_Aaphrophilus@YP_003007650            NSQIAEEL-E LPPVKVHCSI LAEDAIKAAI ADYKAKKGS- -----------
694569_Aactinomycetemc@YP_006286355         NSQIAEEL-E LPPVKVHCSI LAEDAIKAAI ADYKAKKGS- -----------
862965_Hparainfluenzae@YP_004822800         NSQIAEEL-E LPPVKVHCSI LAEDAIKAAI ADYKNKQGK- -----------
221988_Msucciniciprodu@YP_088916            NSQIAEEL-E LPPVKVHCSI LAEDAIKAAI ADYKSKKGA- -----------
339671_Asuccinogenes@YP_001344170           NSQIAEEL-E LPPVKVHCSI LAEDAIKAAI ADYKAKKGE- -----------
550540_Fbalearica@YP_003914035              NTAIAEEL-A LPPVKIHCSI LAEDAIKAAI ADYKSKQDK- -----------
396595_Tsp@YP_003460082                     NTQIAEEL-A LPPVKIHCSV LAEDAIKAAI SNYQERHGQN EQ-KTA----
413404_CRuthia@YP_903798                    NSDIAEEL-S LPPVKIHCSV LAEDAIKAAI NDIKSKA--- -----------
412965_CVesicomyosociu@YP_001219373         NSDIAEEL-S LPPVKIHCSV LAEDAIKAAI NDIKSKI--- -----------
1208921_CKinetoplastiba@YP_007448562        NMQIAEEL-A LPPVKIHCSI LAEDAIRSAI SDYKLKNKID -----------
561501_Baphidicola@YP_002468877             NTTIVEEL-D LPPVKIHCSI LAEDAIKAAI SDYKRKKIN- -----------
357244_Otsutsugamushi@YP_001248706          NSKIAEHL-A LLPIKMHCSM LAEDAIKAAI VDYKSKQNKT -----------
1003201_Rtyphi@YP_005427548                 NTEIAKEL-S LPPVKLHCSL LAEDAIKAAI ADYKQKKGNK KD-S------
1105111_CRickettsia@YP_005365544            NTEIAKEL-S LPPVKLHCSL LAEDAIKAAI ADYKQKKESK KD-S------
272944_Rconorii@NP_360366                   NTEIAKEL-S LPPVKLHCSL LAEDAIKAAI ADYKQKKESK KD-S------
452659_Rrickettsii@YP_001650107             NTEIAKEL-S LPPVKLHCSL LAEDAIKAAI ADYKQKKESK KD-S------
347255_Rafricae@YP_002845312                NTEIAKEL-S LPPVKLHCSL LAEDAIKAAI ADYKQKKESK KD-S------
562019_Rpeacockii@YP_002916645              NTEIAKEL-S LPPVKLHCSL LAEDAIKAAI ADYKQKKESK KD-S------
1105113_Rrhipicephali@YP_005390607          NTEIAKEL-S LPPVKLHCSL LAEDAIKAAI ADYKQKKESK KD-S------
416276_Rmassiliae@YP_001499486              NTEIAKEL-S LPPVKLHCSL LAEDAIKAAI ADYKQKKESK KD-S------
1105108_Rparkeri@YP_005392983               NTEIAKEL-S LPPVKLHCSL LAEDAIKAAI ADYKQKKESK KD-S------
1105114_Rmontanensis@YP_005391246           NTEIAKEL-S LPPVKLHCSL LAEDAIKAAI ADYKQKKESK KD-S------
1032845_Rheilongjiangen@YP_004764467        NTEIAKEL-S LPPVKLHCSL LAEDAIKAAI ADYKQKKESK KD-S------
652620_Rjaponica@YP_004884928               NTEIAKEL-S LPPVKLHCSL LAEDAIKAAI ADYKQKKESK KD-S------
481009_Rphilipii@YP_005300823               NTEIAQEL-S LPPVKLHCSL LAEDAIKAAI ADYKQKKESK KD-S------
1105109_Rslovaca@YP_005426472               NTEIAKEL-S LPPVKLHCSL LAEDAIKAAI ADYKQKKESK KD-S------
1105107_Rcanadensis@YP_005299497            NTEIAKEL-S LPPVKLHCSL LAEDAIKAAI ADYKQKKQAK KT-LKT----
336407_Rbellii@YP_538116                    NTEIAKEL-S LPPVKLHCSL LAEDAIKAAI ADYKLKKENK KD-S------
315456_Rfelis@YP_246860                     NTEIAKEL-S LPPVKLHCSL LAEDAIKAAI ADYKQKKESK KD-S------
293614_Rakari@YP_001493572                  NTEIAKEL-S LPPVKLHCSL LAEDAIKAAI ADYKQKKESK KD-S------
1105110_Raustralis@YP_005414962             NTEIAREL-S LPPVKLHCSL LAEDAIKAAI ADYKQKKESK KD-S------
434131_Nristicii@YP_003081518               NTEIASTL-C LPPIKMHCSM LAEDAIKAAI KDFREKQAAS ST-EEANNEN
222891_Nsennetsu@YP_506192                  NTEIASTL-C LPPIKMHCSM LAEDAIKAAI KDFREKQVTS ST-EEAGNEN
330214_CNitrospira@YP_003799619             NTDIVQEL-N LPPVKIHCSV LAEDAIKAAL ADYQKKADSK -----------
984262_Sgrandis@YP_005322201                NMAIVEEL-E LPPVKIHCSV LAEDAIKAAI KDYQEKNAKA -----------
100901_Wendosymbiont@YP_006555868           NTQIVKEL-S LPPVKIHCSV LAEDAIKAAI HDYQSKQSKS NN---------
66084_Wsp@YP_002727379                      NTQIVEEL-S LPPVKIHCSV LAEDAIKAAI HDYQSKQKN- -----------
```

```
                                           99LPPVK103   X108
                                             -------      *
1423892_Emuris@YP_008929080            NTVLVKEL-S LPPVKIHCSL LAEDAVKAAI NDYNMKQANK KD-KLSSLSE
574556_Acentrale@YP_003328545          NTVLAKEL-S LPPVKIHCSL LAEDAVKAAV HDYKSKQQIA KG-DKC----
320483_Amarginale@YP_002563600         NTVLAKEL-S LPPVKIHCSL LAEDAVKAAV HDYKSKQQIA KG-DKC----
1184253_Aphagocytophilu@YP_008332775   NTVLAKEL-S LPPVKIHCSI LAEDAVKAAI NDYRKKQEAV GN--------
205920_Echaffeensis@YP_507440          NTVLAKEL-S LPPVKIHCSL LAEDAVKAAI KDYNMKQATK KD-KLSSSNE
254945_Eruminantium@YP_180281          NTVLAKEL-S LPPVKIHCSL LAEDAVKAAI NDYHMKQANK KN-ATKNPNE
254945_Eruminantium@YP_197308          NTVLAKEL-S LPPVKIHCSL LAEDAVKAAI NDYHMKQANK KN-ATKNPNE
269484_Ecanis@YP_303050                NTVLAKEL-S LPPVKIHCSL LAEDAIKAAI NDYNMKQENR KS-NTAFSSD
517418_Cthalassium@YP_001997584        NTALVSEL-S LPPVKIHCSV LAEDAIKAAI DDFRKKQAAK HA-KSETSAS
485917_Pheparinus@YP_003091837         NMDIVEEL-A LPPVKIHCSV LAEDAIKSAI NDYRVKNGME PF-ELAKSHH
762903_Psaltans@YP_004273747           NMDIVEEL-A LPPVKIHCSV LAEDAIKSAI NDYRVKNGLP AL-EIEKSHH
929556_Scanadensis@YP_006255074        NMDIVEEL-A LPPVKIHCSV LAEDAIKSAI NDYRVKNGME PI-ALEKSHH
760192_Hhydrossis@YP_004446110         NMTIVEEL-A LPPVKIHCSV LAEDAIRGAI ADYQQKNGIE VA-VAEKAH-
1162668_Lferrooxidans@YP_005469656     NTDIVQEL-N LPPVKIHCSV LAEDAIKGAI QDYQDKKSR- ----------
1048260_Lferriphilum@YP_006765680      NTDIVQEL-N LPPVKIHCSV LAEDAIKSAI NDYRAKKTQT SE-KSEKANV
240015_Acapsulatum@YP_002755573        NTDIVKEL-A LPPVKIHCSV LAEDAIRAAI GDWKKKNGVA ET-EAAQQTV
204669_CKoribacter@YP_589562           NTDIVKEL-S LPPVKIHCSV LAEDAIRAAI GDWKKKNNQ- ----------
1198114_Gtundricola@YP_004219465       NTDIVKEL-A LPPVKIHCSV LAEDAIRAAI GDWKKKNNVA ET-EGAAVAV
926566_Troseus@YP_006423699            NTEIVKEL-A LPPVKIHCSV LAEDAIRAAI GDWKKKNNVA ET-EPALVGA
401053_Tsaanensis@YP_004180969         NTDIVKEL-A LPPVKIHCSV LAEDAIRAAI GDWKKKNGQP ES-EPALVGA
682795_Gmallensis@YP_005055878         NTDIVKEL-A LPPVKIHCSV LAEDAIRAAI GDWKKKNGVA EE-AGVAVAA
696127_CMidichloria@YP_004679282       NSEIAQEL-S LPPVKIHCSV LAEDAISSAI RDYKEKWEIG DK--------
871271_CZinderia@YP_003878048          NLDIANFL-L LSPIKIHCSI LAEEVIKFSI KNYYYKNNYK YD-Y------
657324_Bfibrisolvens@YP_007819038      NKAVTEALDG LPAHKLHCSV LAEEAIKSAV KNYYDNNGIE YD-PKDFPDP
768670_Cnitroreducens@YP_004051749     NNAIVEALDG LPPAKIHCSV MAEEAIEAAL KDYYKRIGKD PS-IVDEMKA
717231_Fsinusarabici@YP_004603876      NNAIVEALEG LPPAKIHCSV MAEEAIEAAL KDYYERVGKD PK-IVDEIKE
639282_Ddesulfuricans@YP_003496772     NEAIVEALDG LPPAKIHCSV MAEEAIEAAL KDYYQRIGKD PK-IVDEMKE
522772_Dacetiphilus@YP_003503972       NDAIVEALGG LPPAKIHCSV MAEEAIEEAL KDYYKSKGED PS-VVDDMKA
693746_Ovalericigenes@YP_004880314     NKAVVEALDG LPAYKLHCSV LAEQAVKAAV KDYYDKNGIP YG-KDLTICN
657321_Rbromii@YP_007781362            NKAVMEALDG LPPVKVHCSV LAEQAIKAAV SDYYKRRGVD PE-PIVGNVP
657322_Fprausnitzii@YP_007799942       NKAVVEALEG LPPVKVHCSV LAEQAVKAAL SDYYRRQGID PE-PIVGKLE
213810_Rchampanellensi@YP_007828576    NKAVVEALDG LPAVKLHCSV LAEQAIKAAL SDYYRRQGID PG-PLVGEVH
657319_[siraeum@YP_007775950           NKAVVEALDG LPPAKLHCSV LAEQAIKAAI ADYYTRQGID PT-PFVGEIK
293826_Ametalliredigen@YP_001320267    NKAVAEALDG LPPVKMHCSV LAEQAVKAAI YDYAQKNNLH YE-DLDDFNP
350688_Aoremlandii@YP_001513205        NKAVAEALDG LPPVKMHCSV LAEQAVKAAI YNYAKENNVH YE-ELEGFVP
546269_Falocis@YP_005054244            NKAVAEALDG LPPVKMHCSV LAEEAVVAAL QDYAKKNNLE IP-GITDRVL
1511_[sticklandii@YP_003936518         NKAVAEALDG LPPVKMHCSV LAEQAIKKAL QDYALKNNLT IE-GLTDVEF
699034_Pdifficile@YP_006198328         NKAVAEALDG LPPVKMHCSV LAEQAVKAAL IDYAQKNNIH IP-ELDGYVI
1128398_[acidurici@YP_006788189        NKAVVEALDG LPPAKIHCSV LAEQAVKSAL IDYAKKNNID IP-ELEKFEL
572544_Ipolytropus@YP_003967871        NKAVAEALDG LPPAKMHCSV LAEEGIKAAI EDYMGKK--- ----------
469604_Fnucleatum@YP_008477745         NKAVVEALGG LPAVKMHCSV LAEEAIKMAI EDYIAKRDGK KA-Q------
580340_Tlienii@YP_004932350            NKDVLEELGG LPPQKIHCSL LAEEGIKAAI EDYRRRKEGK K---------
891968_Amobile@YP_006444325            NKDVAEALGG LPPQKLHCSL LAEEGIKAAI EDYLAKRGND NK-KESGEKQ
525903_Tacidaminovoran@YP_003316727    NKDVAEALGG LPPEKVHCSL LAEQGIRAAI EDYLRRQRGE SP-EPTGGSC
697281_Maustraliensis@YP_004463371     NKTVADALDG LPAIKMHCSV LAEDGLRAAI EDYKRKMGLI TE-DEMAKPH
309799_Dthermophilum@YP_002251521      NKAVTEALDG LPPNKLHCSV LAEEAIKAAI EDYLNKQKNK ----------
368407_Mmarisnigri@YP_001047821        NTDVVDALGG LPEPKVHCSL LARDAIRAAI DDFRTRRGLE PL-ERGSGCC
903814_Elimosum@YP_003961665           NSEVVEALGG LPTEKIHCSV LAAEAIQAAI EDYKSKHNA- ----------
931626_Awoodii@YP_005268656            NSDVVDALGG LPSEKIHCSV LAAEAIQAAI ADYQNNKA-- ----------
410358_Mlabreanum@YP_001029708         NKAVAEALDG LPPNKMHCSV LAEETIHKAI NDYLVKSGRE PW-GDEECSS
679926_Mpetrolearius@YP_003895855      NKAVAEALEG LPPQKLHCSV LAEEAIHKAI NDYRAKNGLE PW-EEKGGHQ
86416_Cpasteurianum@YP_007940944       NKAVAEALDG LPPVKMHCSV LAEEAIHKAI NAYRESVGLE AW-EMKTHSE
929506_Cbotulinum@YP_004396224         NKAVAEALEG LPPVKMHCSV LAEEAIHTAI NDYRVKQGLE PW-KMKVHSD
386415_Cnovyi@YP_878355                NKAVAEALEG LPPVKMHCSV LAEEAIHTAI NDYRVKQGLE PW-DMKVHSD
748727_Cljungdahlii@YP_003779382       NKAVAEALDG LPPVKMHCSV LAEEAIHKAI NDYRESQGLE PW-ATKNHSE
1341692_Cautoethanogenu@YP_008700753   NKAVAEALDG LPPVKMHCSV LAEEAIHKAI NDYRESQGLE PW-ATKNHSE
747365_Tnarugense@YP_004437867         NKAVAEALDG LPPIKMHCSV LAEEAIHKAI NDYRKSHNLE LW-EERTHAE
651822_Ffastidiosum@YP_007827223       NQAVAEALDG LPPVKMHCSV LAEEAIHAAL NDYRTKHGMA AI-PMEKHDD
572547_Acolombiense@YP_003553035       NSAVAKALDG LPPIKMHCSV LAEEAIHGAI NDYRRRQGLE PW-EDKSHDE
212717_Ctetani@NP_781697               NKAVADALDG LPPVKMHCSV LAEEAIHKAI NDYRESIGLE PW-EMKVHSD
431943_Ckluyveri@YP_001394709          NKAVADALDG LPPVKMHCSV LAEEAIHKAI NDYRESQGLE PW-KIQTHSE
573061_Ccellulovorans@YP_003843335     NKAVAEALDG LPPVKMHCSV LAEEAIHKAI NDYRVKNGLE PI-ATEHHSD
508767_Cbotulinum@YP_001920515         NKAVAEALDG LPPVKMHCSV LAEEAIHKAI NDYRAANGLD VI-PMKEHDH
931276_Csaccharoperbut@YP_007454264    NKAVAEALDG LPPVKMHCSV LAEEAIHKAI NDYRANNGLE VI-PMEEHSD
290402_Cbeijerinckii@YP_001308237      NKAVAEALDG LPPVKMHCSV LAEEAIHKAI NDYRAKNGLE VI-PMEEHSD
1345695_Csaccharobutyli@YP_008674363   NKAVAEALDG LPPVKMHCSV LAEEAIHKAI NDYRVNNGLE AK-PMEEHGD
269797_Mbarkeri@YP_305925              NKAVAEALDG LPPIKMHCSM LAEEAIHEAI NDYLKKKGLE PW-TE-----
188937_Macetivorans@NP_617616          NKAVAEALDG LPPIKMHCSM LAEEAIHEAI NDYLQKKGLE PW-D------
195103_Cperfringens@YP_696469          NKAVAEALDG LPPVKMHCSV LAEEAIHKAI NDYRQRQGLE PW-DMKEHSH
593750_Mformicica@YP_007249963         NKAVADALEG LPPIKMHCSV LAEEGIHKAI NNYRKKQGLP EW-EEKNPHS
456442_Mboonei@YP_001405283            NKAVAEALNG LPPIKMHCSV LAEEGIHKAI NDYRVKHGLE PW-QETSSHA
521011_Mpalustris@YP_002466865         NKAVAEALGG LPPIKMHCSV LAEEGIHKAI NDYRVKQGLE PI-PDPHAHA
323259_Mhungatei@YP_503638             NKAVAEALEG LPPIKMHCSV LAEEGIHKAI NDYRIKNGLE PW-EEKGGHT
547558_Mmahii@YP_003541558             NKAVAEALEG LPPVKMHCSV LAEEGIHKAI NDYREKQGLK PL-EEKPDAE
644295_Mevestigatum@YP_003727500       NKAVADALEG LPSIKMHCSV LAEEAIHKAI NDYRQKQNLP LW-GEKEEEK
351627_Csaccharolyticu@YP_001181022    NKAVAEALDG LPANKLHCSV LAEEAIKAAI EDYLNKQKQQ TQ-N------
521460_Cbescii@YP_002573580            NKAVAEALDG LPANKLHCSV LAEEAIKAAI EDYLSKQKSK NT-N------
632292_Chydrothermalis@YP_003992133    NKAVAEALDG LPANKLHCSV LAEEAIKAAI EDYLSKQKSK NT-N------
632348_Ckronotskyensis@YP_004023692    NKAVAEALDG LPANKLHCSV LAEEAIKAAI EDYLSKQKSK NT-N------
632335_Ckristjanssonii@YP_004026829    NKAVAEALDG LPANKLHCSV LAEEAIKAAI EDYLSKQKSK NT-N------
632516_Clactoaceticus@YP_004798736     NKAVAEALDG LPANKLHCSV LAEEAIKAAI EDYLSKQKSK NT-N------
608506_Cobsidiansis@YP_003840166       NKAVAEALDG LPANKLHCSV LAEEAIKAAI EDYLSKQKSK NT-N------
632518_Cowensensis@YP_004002802        NKAVAEALDG LPANKLHCSV LAEEAIKAAI EDYLSKQKSK NT-N------
1121335_[stercorarium@YP_007372524     NKAVVEALDG LPPAKLHCSV LAEEAIAAAI ADYKRRHGLD VE-NSECQGC
1121335_[stercorarium@YP_007678972     NKAVVEALDG LPPAKLHCSV LAEEAIAAAI ADYKRRHGLD VE-NSECQGC
755731_Csp@YP_005148252                NKAVAEALDG LPPAKMHCSN LAEEAIAAAL TDYRKRNGLI DP-NEGEDCT
394503_Ccellulolyticum@YP_002506259    NKAVAEALDG LPPAKMHCSN LAEEAIAAAL TDYRKRNGLV DS-NEGGDCT
720554_[clariflavum@YP_005046370       NKAVAEALDG LPPVKMHCSN LAEEGIKAAI EDYLKKTGRL SK-ECESEVC
```

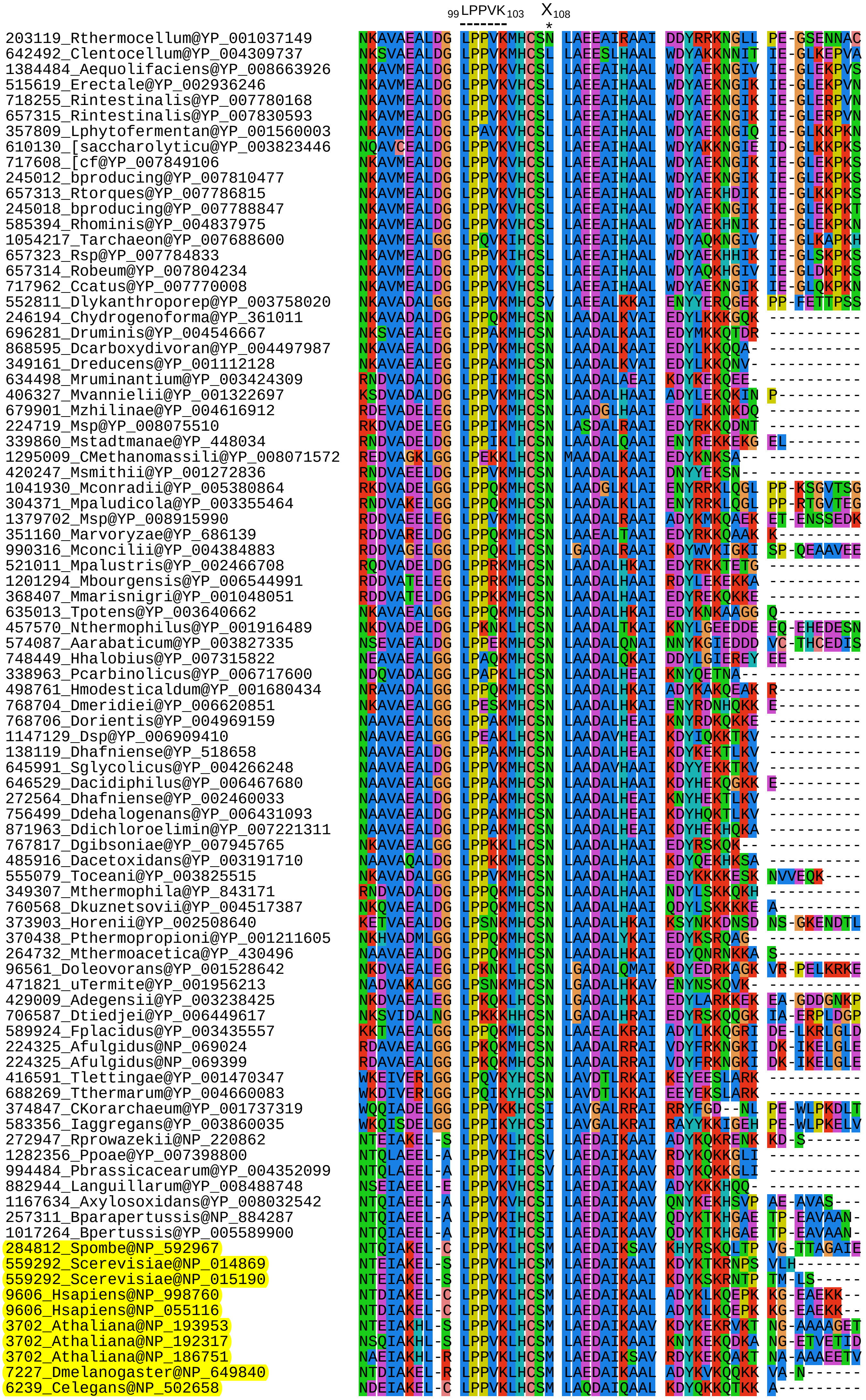

99 LPPVK 103
X 108
*
203119_Rthermocellum@YP_001037149 NKAVAEALDG LPPVKMHCSN LAEEAIRAAI DDYRRKNGLL PE-GSENNAC
642492_Clentocellum@YP_004309737 NKSVAEALDG LPPVKMHCSL LAEESLHAAL WDYAKKNNIT IE-GLKEPVA
1384484_Aequolifaciens@YP_008663926 NKAVMEALDG LPPVKVHCSL LAEEAIHAAL WDYAEKNGIV IE-GLEKPVS
515619_Erectale@YP_002936246 NKAVMEALDG LPPVKVHCSL LAEEAIHAAL WDYAEKNGIK IE-GLEKPVN
718255_Rintestinalis@YP_007780168 NKAVMEALDG LPPVKVHCSL LAEEAIHAAL WDYAEKNGIK IE-GLERPVN
657315_Rintestinalis@YP_007830593 NKAVMEALDG LPPVKVHCSL LAEEAIHAAL WDYAEKNGIK IE-GLERPVN
357809_Lphytofermentan@YP_001560003 NKAVMEALDG LPAVKVHCSL LAEEAIHAAL WDYAEKNGIQ IE-GLKKPKN
610130_[saccharolyticu@YP_003823446 NQAVCEALDG LPPVKVHCSL LAEEAIHAAL WDYAKKNGIE ID-GLKKPKS
717608_[cf@YP_007849106 NKAVMEALDG LPPVKVHCSL LAEEAIHAAL WDYAEKNGIK IE-GLEKPKS
245012_bproducing@YP_007810477 NKAVMEALDG LPPVKVHCSL LAEEAIHAAL WDYAEKNGIK IE-GLEKPKS
657313_Rtorques@YP_007786815 NKAVMEALDG LPPVKVHCSL LAEEAIHAAL WDYAEKHDIK IE-GLKKPKS
245018_bproducing@YP_007788847 NKAVMEALDG LPPVKVHCSL LAEEAIHAAL WDYAEKNGIK IE-GLEKPKT
585394_Rhominis@YP_004837975 NKAVMEALDG LPPVKVHCSL LAEEAIHAAL WDYAEKHNIK IE-GLEKPKN
1054217_Tarchaeon@YP_007688600 NKAVMEALGG LPQVKIHCSL LAEEAIHAAL WDYAQKNGIV IE-GLKAPKH
657323_Rsp@YP_007784833 NKAVMEALDG LPPVKIHCSL LAEEAIHAAL WDYAEKHHIK IE-GLSKPKS
657314_Robeum@YP_007804234 NKAVMEALDG LPPVKVHCSL LAEEAIHAAL WDYAQKHGIV IE-GLDKPKS
717962_Ccatus@YP_007770008 NKAVMEALDG LPPVKVHCSL LAEEAIHAAL WDYAEKNGIK IE-GLQKPKN
552811_Dlykanthroporep@YP_003758020 NKAVADALGG LPPVKMHCSV LAEEALKKAI ENYYERQGEK PP-FETTPSS
246194_Chydrogenoforma@YP_361011 NKAVADALDG LPPQKMHCSN LAADALKVAI EDYLKKKGQK ----------
696281_Druminis@YP_004546667 NKSVAEALEG LPPAKMHCSN LAADALKAAI EDYMKKQTDR ----------
868595_Dcarboxydivoran@YP_004497987 NKAVAEALDG LPPVKMHCSN LAADALKAAI EDYLKKQQA- ----------
349161_Dreducens@YP_001112128 NKAVAEALEG LPPAKMHCSN LAADALKVAI EDYLKKQNV- ----------
634498_Mruminantium@YP_003424309 RNDVADALDG LPPIKMHCSN LAADALAEAI KDYKEKQEE- ----------
406327_Mvannielii@YP_001322697 KSDVADALDG LPPVKMHCSN LAADALHAAI ADYLEKQKIN P---------
679901_Mzhilinae@YP_004616912 RDEVADELEG LPPVKMHCSN LAADGLHAAI EDYLKKNKDQ ----------
224719_Msp@YP_008075510 RKDVADELEG LPPIKMHCSN LASDALRAAI EDYRKKQDNT ----------
339860_Mstadtmanae@YP_448034 RNDVADELDG LPPIKLHCSN LAADALQAAI ENYREKKEKG EL--------
1295009_CMethanomassili@YP_008071572 REDVAGKLGG LPEKKLHCSN MAADALKAAI EDYKNKSA-- ----------
420247_Msmithii@YP_001272836 RNDVAEELDG LPPVKMHCSN LAADALKAAI DNYYEKSN-- ----------
1041930_Mconradii@YP_005380864 RKDVADELGG LPPQKMHCSN LAADGLKLAI ENYRRKLQGL PP-KSGVTSG
304371_Mpaludicola@YP_003355464 RNDVAKELGG LPPQKMHCSN LAADALKLAI ENYRRKLQGL PP-RTGVTEG
1379702_Msp@YP_008915990 RDDVAEELEG LPPVKMHCSN LAADALRAAI ADYKMKQAEK ET-ENSSEDK
351160_Marvoryzae@YP_686139 RDDVARELDG LPPQKMHCSN LAAEALTAAI EDYRKKQAAK K---------
990316_Mconcilii@YP_004384883 RDDVAGELGG LPPQKLHCSN LGADALRAAI KDYWVKIGKI SP-QEAAVEE
521011_Mpalustris@YP_002466708 RQDVADELDG LPPRKMHCSN LAADALHKAI EDYRKKTETG ----------
1201294_Mbourgensis@YP_006544991 RDDVATELEG LPPRKMHCSN LAADALHAAI RDYLEKEKKA ----------
368407_Mmarisnigri@YP_001048051 RDDVATELDG LPPKKMHCSN LAADALHAAI EDYREKQKKE ----------
635013_Tpotens@YP_003640662 NKAVAEALGG LPPQKMHCSN LAADALHKAI EDYKNKAAGG Q---------
457570_Nthermophilus@YP_001916489 NKDVADELDG LPKNKLHCSN LAADALTKAI KNYLGEEDDE EQ-EHEDESN
574087_Aarabaticum@YP_003827335 NSEVAEALDG LPPEKMHCSN LAADALQNAI NNYKGIEDDD VC-THCEDIS
748449_Hhalobius@YP_007315822 NEAVAEALGG LPAQKMHCSN LAADALQKAI DDYLGIEREY EE--------
338963_Pcarbinolicus@YP_006717600 NDQVADALGG LPAPKLHCSN LAADALHEAI KNYQETNA-- ----------
498761_Hmodesticaldum@YP_001680434 NRAVADALGG LPPQKMHCSN LAADALHKAI ADYKAKQEAK R---------
768704_Dmeridiei@YP_006620851 NKAVAEALGG LPESKMHCSN LAADALHKAI ENYRDNHQKK E---------
768706_Dorientis@YP_004969159 NAAVAEALGG LPPAKMHCSN LAADALHEAI KNYRDKQKKE ----------
1147129_Dsp@YP_006909410 NAAVAEALGG LPEAKLHCSN LAADAVHEAI KDYIQKKTKV ----------
138119_Dhafniense@YP_518658 NAAVAEALDG LPPAKMHCSN LAADALHEAI KDYKEKTLKV ----------
645991_Sglycolicus@YP_004266248 NAAVAEALDG LPPVKMHCSN LAADAVHEAI KDYYEKKTKV ----------
646529_Dacidiphilus@YP_006467680 NAAVAEALGG LPPAKMHCSN LAADALHAAI KDYHEKQGKK E---------
272564_Dhafniense@YP_002460033 NAAVAEALDG LPPAKMHCSN LAADALHEAI KNYHEKTLKV ----------
756499_Ddehalogenans@YP_006431093 NAAVAEALDG LPPAKMHCSN LAADALHEAI KDYHQKTLKV ----------
871963_Ddichloroelimin@YP_007221311 NAAVAEALDG LPPAKMHCSN LAADALHEAI KDYHEKHQKA ----------
767817_Dgibsoniae@YP_007945765 NKAVAEALGG LPPKKLHCSN LAADALHAAI EDYRSKQK-- ----------
485916_Dacetoxidans@YP_003191710 NAAVAQALDG LPPKKMHCSN LAADALHAAI KDYQEKHKSA ----------
555079_Toceani@YP_003825515 NKAVADALGG LPPVKMHCSN LAADALHAAI EDYKKKKESK NVVEQK----
349307_Mthermophila@YP_843171 RNDVADALDG LPPQKMHCSN LAADALHAAI NDYLSKKQKH ----------
760568_Dkuznetsovii@YP_004517387 NKQVAEALDG LPPQKMHCSN LAADALHAAI QDYLSKKKKE A---------
373903_Horenii@YP_002508640 KETVAEALDG LPSNKMHCSN LAADALHKAI KSYNKKDNSD NS-GKENDTL
370438_Pthermopropioni@YP_001211605 NKHVADMLGG LPPQKMHCSN LAADALYKAI EDYKSRQAG- ----------
264732_Mthermoacetica@YP_430496 NAAVAEALDG LPPQKMHCSN LAADALHKAI EDYQNRNKKA S---------
96561_Doleovorans@YP_001528642 NKDVAEALEG LPKNKLHCSN LGADALQMAI KDYEDRKAGK VR-PELKRKE
471821_uTermite@YP_001956213 NADVAKALGG LPSNKMHCSN LGADALHKAV ENYNSKQVK- ----------
429009_Adegensii@YP_003238425 NKDVAEALEG LPKQKLHCSN LGADALHKAI EDYLARKKEK EA-GDDGNKP
706587_Dtiedjei@YP_006449617 NKSVIDALNG LPKKKHHCSN LGADALHRAI EDYRSKQQGK IA-ERPLDGP
589924_Fplacidus@YP_003435557 KKTVAEALGG LPPQKMHCSN LAAEALKRAI ADYLKKQGRI DE-LKRLGLD
224325_Afulgidus@NP_069024 RDAVAEALGG LPKQKMHCSN LAADALRRAI VDYFRKNGKI DK-IKELGLE
224325_Afulgidus@NP_069399 RDAVAEALGG LPKQKMHCSN LAADALRRAI VDYFRKNGKI DK-IKELGLE
416591_Tlettingae@YP_001470347 WKEIVERLGG LPQVKYHCSN LAVDTLRKAI KEYEESLARK ----------
688269_Tthermarum@YP_004660083 WKDIVERLGG LPQIKYHCSN LAVDTLKKAI EEYEKSLARK ----------
374847_CKorarchaeum@YP_001737319 WQQIADELGG LPPVKKHCSI LAVGALRRAI RRYFGD--NL PE-WLPKDLT
583356_Iaggregans@YP_003860035 WKQISDELGG LPPIKYHCSI LAVGALKRAI RAYYKKIGEH PE-WLPKELV
272947_Rprowazekii@NP_220862 NTEIAKEL-S LPPVKLHCSL LAEDAIKAAI ADYKQKRENK KD-S------
1282356_Ppoae@YP_007398800 NTQLAEEL-A LPPVKIHCSV LAEDAIKAAV RDYKQKKGLI ----------
994484_Pbrassicacearum@YP_004352099 NTQLAEEL-A LPPVKIHCSV LAEDAIKAAV RDYKQKKGLI ----------
882944_Languillarum@YP_008488748 NSEIAEEL-E LPPVKVHCSI LAEDAIKAAV ADYKKKHQQ- ----------
1167634_Axylosoxidans@YP_008032542 NTQIAEEL-A LPPVKVHCSI LAEDAIKAAV QNYKEKHSVP AE-AVAS---
257311_Bparapertussis@NP_884287 NTQIAEEL-A LPPVKIHCSI LAEDAIKAAV QDYKTKHGAE TP-EAVAAN-
1017264_Bpertussis@YP_005589900 NTQIAEEL-A LPPVKIHCSI LAEDAIKAAV QDYKTKHGAE TP-EAVAAN-
284812_Spombe@NP_592967 NTQIAKEL-C LPPVKLHCSM LAEDAIKSAV KHYRSKQLTP VG-TTAGAIE
559292_Scerevisiae@NP_014869 NTEIAKEL-S LPPVKLHCSM LAEDAIKAAI KDYKTKRNPS VLH-------
559292_Scerevisiae@NP_015190 NTEIAKEL-S LPPVKLHCSM LAEDAIKAAI KDYKSKRNTP TM-LS-----
9606_Hsapiens@NP_998760 NTDIAKEL-C LPPVKLHCSM LAEDAIKAAL ADYKLKQEPK KG-EAEKK--
9606_Hsapiens@NP_055116 NTDIAKEL-C LPPVKLHCSM LAEDAIKAAL ADYKLKQEPK KG-EAEKK--
3702_Athaliana@NP_193953 NTEIAKHL-S LPPVKLHCSM LAEDAIKAAV KDYKEKRVKT NG-AAAAGET
3702_Athaliana@NP_192317 NSQIAKHL-S LPPVKLHCSM LAEDAIKAAI KNYKEKQDKA NG-ETVETID
3702_Athaliana@NP_186751 NAEIAKHL-R LPPVKLHCSM LAEDAIKSAV RDYKEKQAKT NA-AAAEETV
7227_Dmelanogaster@NP_649840 NTDIAKEL-R LPPVKLHCSM LAEDAIKAAL ADYKVKQQKK VA-N------
6239_Celegans@NP_502658 NDEIAKEL-C LPPVKLHCSM LAQDAIQAAL KDYQKKQTKK A---------

```
                                      201
243233_Mcapsulatus@YP_112782          -AA------- ---------- ---------- ----------
458817_Shalifaxensis@YP_001673798     ---------- ---------- ---------- ----------
398579_Spealeana@YP_001501349         ---------- ---------- ---------- ----------
557598_Lhongkongensis@YP_002796852    ---------- ---------- ---------- ----------
521006_Ngonorrhoeae@YP_002001906      ---------- ---------- ---------- ----------
489653_Nlactamica@YP_004048810        ---------- ---------- ---------- ----------
935589_Nmeningitidis@YP_005903962     ---------- ---------- ---------- ----------
395495_Lcholodnii@YP_001790394        -KAAA----- ---------- ---------- ----------
999541_Bgladioli@YP_004361365         ---------- ---------- ---------- ----------
983917_Rgelatinosus@YP_005439140      -SPSSPDQQH SEAA------ ---------- ----------
1416914_Ppnomenusa@YP_008837319       ---------- ---------- ---------- ----------
1380774_Psp@YP_008881415              ---------- ---------- ---------- ----------
1249661_Bthailandensis@YP_007917844   ---------- ---------- ---------- ----------
320388_Bmallei@YP_993528              ---------- ---------- ---------- ----------
536230_Bpseudomallei@YP_002897469     ---------- ---------- ---------- ----------
395019_Bmultivorans@YP_001579332      ---------- ---------- ---------- ----------
626418_Bglumae@YP_002912250           ---------- ---------- ---------- ----------
365044_Pnaphthalenivor@YP_981803      -QPVCASSTP APTL------ ---------- ----------
266265_Bxenovorans@YP_553871          -ADQPVCSSN PN-------- ---------- ----------
269482_Bvietnamiensis@YP_001115172    -VCTSTPN-- ---------- ---------- ----------
583345_Mmobilis@YP_003049105          ---------- ---------- ---------- ----------
666681_Mversatilis@YP_003674921       ---------- ---------- ---------- ----------
391038_Bphymatum@YP_001857685         ---------- ---------- ---------- ----------
398577_Bambifaria@YP_001808732        ---------- ---------- ---------- ----------
269482_Bvietnamiensis@YP_001120041    ---------- ---------- ---------- ----------
406425_Bcenocepacia@YP_001765425      ---------- ---------- ---------- ----------
1009846_Bcepacia@YP_006615433         ---------- ---------- ---------- ----------
482957_Blata@YP_369669                ---------- ---------- ---------- ----------
748280_Psp@YP_004848592               ---------- ---------- ---------- ----------
1229205_Bphenoliruptrix@YP_006834626  ---------- ---------- ---------- ----------
1163617_Sdenitrificans@YP_008546117   -DCSK----- ---------- ---------- ----------
266265_Bxenovorans@YP_559453          ---------- ---------- ---------- ----------
398527_Bphytofirmans@YP_001896197     ---------- ---------- ---------- ----------
375286_Jsp@YP_001352943               ---------- ---------- ---------- ----------
204773_Harsenicoxydans@YP_001100494   ---------- ---------- ---------- ----------
580332_Slithotrophicus@YP_003524869   -K-------- ---------- ---------- ----------
159087_Daromatica@YP_285167           ---------- ---------- ---------- ----------
882378_Brhizoxinica@YP_004029636      -EAAA----- ---------- ---------- ----------
85643_Tsp@YP_002355840                ---------- ---------- ---------- ----------
757424_Hseropedicae@YP_003776230      -PKQYPICAP IAD------- ---------- ----------
522306_CAccumulibacter@YP_003167247   ---------- ---------- ---------- ----------
76114_Aaromaticum@YP_160687           ---------- ---------- ---------- ----------
381666_Reutropha@YP_725668            ---------- ---------- ---------- ----------
977880_Ctaiwanensis@YP_002005171      ---------- ---------- ---------- ----------
1042878_Cnecator@YP_004684939         ---------- ---------- ---------- ----------
266264_Cmetallidurans@YP_583181       ---------- ---------- ---------- ----------
395494_Gcapsiferriform@YP_003846450   -C-------- ---------- ---------- ----------
859655_Rsolanacearum@YP_005996961     ---------- ---------- ---------- ----------
402626_Rpickettii@YP_001898467        ---------- ---------- ---------- ----------
543913_bproteobacteriu@YP_007502653   ---------- ---------- ---------- ----------
62928_Asp@YP_933519                   ---------- ---------- ---------- ----------
640081_Dsuillum@YP_005028573          ---------- ---------- ---------- ----------
452638_Pnecessarius@YP_001797351      ---------- ---------- ---------- ----------
292415_Tdenitrificans@YP_314923       ---------- ---------- ---------- ----------
265072_Mflagellatus@YP_544916         ---------- ---------- ---------- ----------
582744_Mglucosetrophus@YP_003051619   ---------- ---------- ---------- ----------
887061_Msp@YP_004040241               ---------- ---------- ---------- ----------
891398_CTremblaya@YP_004707015        ---------- ---------- ---------- ----------
426114_Tarsenitoxydans@YP_003623866   -AQAA----- ---------- ---------- ----------
75379_Tintermedia@YP_003642782        -AQAA----- ---------- ---------- ----------
937774_Tequigenitalis@YP_004130301    ---------- ---------- ---------- ----------
1008459_Tasinigenitalis@YP_004874474  ---------- ---------- ---------- ----------
634503_Eictaluri@YP_002934567         ---------- ---------- ---------- ----------
718251_Etarda@YP_005700147            ---------- ---------- ---------- ----------
259536_Parcticus@YP_264760            ---------- ---------- ---------- ----------
335284_Pcryohalolentis@YP_580918      ---------- ---------- ---------- ----------
571800_Psp@YP_008163576               ---------- ---------- ---------- ----------
420662_Mpetroleiphilum@YP_001021452   ---------- ---------- ---------- ----------
243365_Cviolaceum@NP_900763           ---------- ---------- ---------- ----------
1208919_CKinetoplastiba@YP_007459239  ---------- ---------- ---------- ----------
983545_Gsp@YP_004435367               ---------- ---------- ---------- ----------
342610_Patlantica@YP_660819           ---------- ---------- ---------- ----------
743299_Aferrivorans@YP_004783591      -SAQAAH--- ---------- ---------- ----------
243159_Aferrooxidans@YP_002425162     -GAQAAH--- ---------- ---------- ----------
1005048_Cfungivorans@YP_004753777     ---------- ---------- ---------- ----------
669502_CProfftella@YP_008343646       ---------- ---------- ---------- ----------
757424_Hseropedicae@YP_003776535      ---------- ---------- ---------- ----------
395495_Lcholodnii@YP_001790080        ---------- ---------- ---------- ----------
257310_Bbronchiseptica@NP_888820      ---------- ---------- ---------- ----------
990288_Acaldus@YP_004748177           -AASH----- ---------- ---------- ----------
990288_Acaldus@YP_004748110           ---------- ---------- ---------- ----------
983917_Rgelatinosus@YP_005436383      ---------- ---------- ---------- ----------
360910_Bavium@YP_786035               ---------- ---------- ---------- ----------
1036672_Akashmirensis@YP_006379415    ---------- ---------- ---------- ----------
340100_Bpetrii@YP_001631386           -AAVAN---- ---------- ---------- ----------
523791_Kkoreensis@YP_003146685        ---------- ---------- ---------- ----------
156889_Mmarinus@YP_866958             ---------- ---------- ---------- ----------
```

```
404589_Asp@YP_001377835               -GDRAELLER GVLKSGI--- ---------- ----------
290397_Adehalogenans@YP_463806        -GDRSALIEK GVLKSGI--- ---------- ----------
447217_Asp@YP_002132996               -GDRSALIEK GVLKSGI--- ---------- ----------
455488_Adehalogenans@YP_002491042     -GDRSALIEK GVLKSGI--- ---------- ----------
378806_Saurantiaca@YP_003955403       ---------- ---------- ---------- ----------
246197_Mxanthus@YP_633158             ---------- ---------- ---------- ----------
483219_Mfulvus@YP_004669542           ---------- ---------- ---------- ----------
1144275_Ccoralloides@YP_005368444     ---------- ---------- ---------- ----------
1278073_Mstipitatus@YP_007362455      ---------- ---------- ---------- ----------
448385_Scellulosum@YP_001617979       -VPAAAAATA SAE------- ---------- ----------
399739_Pmendocina@YP_001188990        ---------- ---------- ---------- ----------
1294143_Pdenitrificans@YP_007659193   ---------- ---------- ---------- ----------
208964_Paeruginosa@NP_252502          ---------- ---------- ---------- ----------
322710_Avinelandii@YP_002801150       ---------- ---------- ---------- ----------
644801_Pstutzeri@YP_007239445         ---------- ---------- ---------- ----------
1207075_Psp@YP_007027941              ---------- ---------- ---------- ----------
1114970_Pfluorescens@YP_005206437     ---------- ---------- ---------- ----------
220664_Pprotegens@YP_262044           ---------- ---------- ---------- ----------
351746_Pputida@YP_001266219           ---------- ---------- ---------- ----------
1435058_Pmonteilii@YP_008958359       ---------- ---------- ---------- ----------
743720_Pfulva@YP_004475358            ---------- ---------- ---------- ----------
264730_Psyringae@YP_273568            ---------- ---------- ---------- ----------
384676_Pentomophila@YP_606728         ---------- ---------- ---------- ----------
1430440_Mgryphiswaldens@YP_008937260  ---------- ---------- ---------- ----------
749219_Mcatarrhalis@YP_003626770      ---------- ---------- ---------- ----------
62977_Asp@YP_046090                   ---------- ---------- ---------- ----------
436717_Aoleivorans@YP_003732380       ---------- ---------- ---------- ----------
400667_Abaumannii@YP_001084660        ---------- ---------- ---------- ----------
871585_Acalcoaceticus@YP_004995269    ---------- ---------- ---------- ----------
511062_Osp@YP_005091814               ---------- ---------- ---------- ----------
318167_Sfrigidimarina@YP_751106       ---------- ---------- ---------- ----------
326297_Samazonensis@YP_927171         ---------- ---------- ---------- ----------
318161_Sdenitrificans@YP_562467       ---------- ---------- ---------- ----------
167879_Cpsychrerythrae@YP_267876      ---------- ---------- ---------- ----------
637905_Sviolacea@YP_003556341         ---------- ---------- ---------- ----------
225849_Spiezotolerans@YP_002311055    ---------- ---------- ---------- ----------
323850_Sloihica@YP_001094441          ---------- ---------- ---------- ----------
425104_Ssediminis@YP_001474605        ---------- ---------- ---------- ----------
392500_Swoodyi@YP_001760156           ---------- ---------- ---------- ----------
60481_Ssp@YP_737869                   ---------- ---------- ---------- ----------
211586_Soneidensis@NP_717861          ---------- ---------- ---------- ----------
693973_Sbaltica@YP_005273706          ---------- ---------- ---------- ----------
399804_Sputrefaciens@YP_006009782     ---------- ---------- ---------- ----------
298386_Pprofundum@YP_128966           ---------- ---------- ---------- ----------
28173_Vnigripulchritu@YP_008621944    ---------- ---------- ---------- ----------
1219076_Valginolyticus@YP_008537102   ---------- ---------- ---------- ----------
150340_Vsp@YP_003286982               ---------- ---------- ---------- ----------
345073_Vcholerae@YP_001216234         ---------- ---------- ---------- ----------
223926_Vparahaemolytic@NP_796976      ---------- ---------- ---------- ----------
882102_Vanguillarum@YP_004566935      ---------- ---------- ---------- ----------
575788_Vsplendidus@YP_002416251       ---------- ---------- ---------- ----------
1129794_Gpsychrophila@YP_007545568    ---------- ---------- ---------- ----------
196600_Vvulnificus@NP_933549          ---------- ---------- ---------- ----------
903510_Vfurnissii@YP_004993913        ---------- ---------- ---------- ----------
338187_Vcampbellii@YP_001444276       ---------- ---------- ---------- ----------
338187_Vcampbellii@YP_008526292       ---------- ---------- ---------- ----------
314282_Psp@YP_007639631               ---------- ---------- ---------- ----------
357804_Pingrahamii@YP_942749          ---------- ---------- ---------- ----------
326442_Phaloplanktis@YP_341160        ---------- ---------- ---------- ----------
234831_Psp@YP_004067531               ---------- ---------- ---------- ----------
595494_Tauensis@YP_002893203          ---------- ---------- ---------- ----------
396588_Tsulfidiphilus@YP_002513559    ---------- ---------- ---------- ----------
396588_Tsulfidiphilus@YP_002514125    ---------- ---------- ---------- ----------
998088_Averonii@YP_004393031          ---------- ---------- ---------- ----------
380703_Ahydrophila@YP_856284          ---------- ---------- ---------- ----------
382245_Asalmonicida@YP_001142383      ---------- ---------- ---------- ----------
342108_Mmagneticum@YP_422390          ---------- ---------- ---------- ----------
1245471_Presinovorans@YP_008101472    ---------- ---------- ---------- ----------
365044_Pnaphthalenivor@YP_982518      ---------- ---------- ---------- ----------
296591_Psp@YP_549004                  ---------- ---------- ---------- ----------
232721_Asp@YP_986388                  ---------- ---------- ---------- ----------
535289_Aebreus@YP_002553097           ---------- ---------- ---------- ----------
365046_Rtataouinensis@YP_004618947    ---------- ---------- ---------- ----------
946483_CSymbiobacter@YP_008681130     ---------- ---------- ---------- ----------
312309_Vfischeri@YP_204001            ---------- ---------- ---------- ----------
595537_Vparadoxus@YP_004156043        -H-------- ---------- ---------- ----------
316275_Asalmonicida@YP_002262235      ---------- ---------- ---------- ----------
391735_Veiseniae@YP_997138            ---------- ---------- ---------- ----------
397945_Acitrulli@YP_970793            ---------- ---------- ---------- ----------
643561_Aavenae@YP_004235248           ---------- ---------- ---------- ----------
398578_Dacidovorans@YP_001565014      ---------- ---------- ---------- ----------
742013_Dsp@YP_004488168               ---------- ---------- ---------- ----------
338969_Aferrireducens@YP_523433       ---------- ---------- ---------- ----------
596154_Adenitrificans@YP_004388278    ---------- ---------- ---------- ----------
688245_Ctestosteroni@YP_003278774     ---------- ---------- ---------- ----------
1266738_Pmirabilis@YP_008398502       ---------- ---------- ---------- ----------
1124991_Mmorganii@YP_007505117        ---------- ---------- ---------- ----------
406817_Xnematophila@YP_003713469      ---------- ---------- ---------- ----------
```

```
291112_Pasymbiotica@YP_003040200      ---------- ---------- ---------- ----------
243265_Pluminescens@NP_930506         ---------- ---------- ---------- ----------
406818_Xbovienii@YP_003468915         ---------- ---------- ---------- ----------
273123_Ypseudotubercul@YP_071365      ---------- ---------- ---------- ----------
393305_Yenterocolitica@YP_001005390   ---------- ---------- ---------- ----------
1346614_Sliquefaciens@YP_008231656    ---------- ---------- ---------- ----------
1348660_Splymuthica@YP_008160622      ---------- ---------- ---------- ----------
399741_Sproteamaculans@YP_001479850   ---------- ---------- ---------- ----------
1249634_Smarcescens@YP_007345780      ---------- ---------- ---------- ----------
187410_Ypestis@NP_668657              ---------- ---------- ---------- ----------
1157951_Pstuartii@YP_006215182        ---------- ---------- ---------- ----------
344609_Sboydii@YP_001881320           ---------- ---------- ---------- ----------
104623_Ssp@YP_008524031               ---------- ---------- ---------- ----------
561229_Dzeae@YP_003003456             ---------- ---------- ---------- ----------
590409_Ddadantii@YP_003334610         ---------- ---------- ---------- ----------
561230_Pcarotovorum@YP_003018589      ---------- ---------- ---------- ----------
561231_Pwasabiae@YP_003258666         ---------- ---------- ---------- ----------
1166016_Psp@YP_006282128              ---------- ---------- ---------- ----------
218491_Patrosepticum@YP_051325        ---------- ---------- ---------- ----------
99287_Senterica@NP_461477             ---------- ---------- ---------- ----------
218493_Sbongori@YP_004731144          ---------- ---------- ---------- ----------
290339_Csakazakii@YP_001436836        ---------- ---------- ---------- ----------
693216_Cturicensis@YP_003211492       ---------- ---------- ---------- ----------
399742_Esp@YP_001177741               ---------- ---------- ---------- ----------
640513_Easburiae@YP_004829762         ---------- ---------- ---------- ----------
693444_Ebacterium@YP_007339349        ---------- ---------- ---------- ----------
290338_Ckoseri@YP_001451852           ---------- ---------- ---------- ----------
484021_Kpneumoniae@YP_002920722       ---------- ---------- ---------- ----------
637910_Crodentium@YP_003366009        ---------- ---------- ---------- ----------
640131_Kvariicola@YP_003438138        ---------- ---------- ---------- ----------
1191061_Koxytoca@YP_006500071         ---------- ---------- ---------- ----------
1286170_Rornithinolytic@YP_007872306  ---------- ---------- ---------- ----------
511145_Ecoli@NP_417024                ---------- ---------- ---------- ----------
585054_Efergusonii@YP_002381825       ---------- ---------- ---------- ----------
198214_Sflexneri@NP_708368            ---------- ---------- ---------- ----------
216599_Ssonnei@YP_005457544           ---------- ---------- ---------- ----------
300267_Sdysenteriae@YP_404261         ---------- ---------- ---------- ----------
716541_Ecloacae@YP_003614361          ---------- ---------- ---------- ----------
701347_Elignolyticus@YP_003940771     ---------- ---------- ---------- ----------
630626_Sblattae@YP_006318650          ---------- ---------- ---------- ----------
1028307_Eaerogenes@YP_004590373       ---------- ---------- ---------- ----------
557723_Hparasuis@YP_002474707         ---------- ---------- ---------- ----------
1005058_Ganatis@YP_004420698          ---------- ---------- ---------- ----------
1075089_Pmultocida@YP_005176279       ---------- ---------- ---------- ----------
205914_Hsomnus@YP_718490              ---------- ---------- ---------- ----------
1171377_Btrehalosi@YP_007548422       ---------- ---------- ---------- ----------
233412_Hducreyi@NP_873560             ---------- ---------- ---------- ----------
416269_Apleuropneumoni@YP_001053631   ---------- ---------- ---------- ----------
696748_Asuis@YP_006816777             ---------- ---------- ---------- ----------
1366053_Mhaemolytica@YP_008338465     ---------- ---------- ---------- ----------
1334187_Hinfluenzae@YP_008544790      ---------- ---------- ---------- ----------
634176_Aaphrophilus@YP_003007650      ---------- ---------- ---------- ----------
694569_Aactinomycetemc@YP_006286355   ---------- ---------- ---------- ----------
862965_Hparainfluenzae@YP_004822800   ---------- ---------- ---------- ----------
221988_Msucciniciprodu@YP_088916      ---------- ---------- ---------- ----------
339671_Asuccinogenes@YP_001344170     ---------- ---------- ---------- ----------
550540_Fbalearica@YP_003914035        ---------- ---------- ---------- ----------
396595_Tsp@YP_003460082               ---------- ---------- ---------- ----------
413404_CRuthia@YP_903798              ---------- ---------- ---------- ----------
412965_CVesicomyosociu@YP_001219373   ---------- ---------- ---------- ----------
1208921_CKinetoplastiba@YP_007448562  ---------- ---------- ---------- ----------
561501_Baphidicola@YP_002468877       ---------- ---------- ---------- ----------
357244_Otsutsugamushi@YP_001248706    ---------- ---------- ---------- ----------
1003201_Rtyphi@YP_005427548           ---------- ---------- ---------- ----------
1105111_CRickettsia@YP_005365544      ---------- ---------- ---------- ----------
272944_Rconorii@NP_360366             ---------- ---------- ---------- ----------
452659_Rrickettsii@YP_001650107       ---------- ---------- ---------- ----------
347255_Rafricae@YP_002845312          ---------- ---------- ---------- ----------
562019_Rpeacockii@YP_002916645        ---------- ---------- ---------- ----------
1105113_Rrhipicephali@YP_005390607    ---------- ---------- ---------- ----------
416276_Rmassiliae@YP_001499486        ---------- ---------- ---------- ----------
1105108_Rparkeri@YP_005392983         ---------- ---------- ---------- ----------
1105114_Rmontanensis@YP_005391246     ---------- ---------- ---------- ----------
1032845_Rheilongjiangen@YP_004764467  ---------- ---------- ---------- ----------
652620_Rjaponica@YP_004884928         ---------- ---------- ---------- ----------
481009_Rphilipii@YP_005300823         ---------- ---------- ---------- ----------
1105109_Rslovaca@YP_005426472         ---------- ---------- ---------- ----------
1105107_Rcanadensis@YP_005299497      ---------- ---------- ---------- ----------
336407_Rbellii@YP_538116              ---------- ---------- ---------- ----------
315456_Rfelis@YP_246860               ---------- ---------- ---------- ----------
293614_Rakari@YP_001493572            ---------- ---------- ---------- ----------
1105110_Raustralis@YP_005414962       ---------- ---------- ---------- ----------
434131_Nristicii@YP_003081518         -TDNKS---- ---------- ---------- ----------
222891_Nsennetsu@YP_506192            -TENKS---- ---------- ---------- ----------
330214_CNitrospira@YP_003799619       ---------- ---------- ---------- ----------
984262_Sgrandis@YP_005322201          ---------- ---------- ---------- ----------
100901_Wendosymbiont@YP_006555868     ---------- ---------- ---------- ----------
66084_Wsp@YP_002727379                ---------- ---------- ---------- ----------
```

```
1423892_Emuris@YP_008929080             ---------- ---------- ---------- ----------
574556_Acentrale@YP_003328545           ---------- ---------- ---------- ----------
320483_Amarginale@YP_002563600          ---------- ---------- ---------- ----------
1184253_Aphagocytophilu@YP_008332775    ---------- ---------- ---------- ----------
205920_Echaffeensis@YP_507440           ---------- ---------- ---------- ----------
254945_Eruminantium@YP_180281           ---------- ---------- ---------- ----------
254945_Eruminantium@YP_197308           ---------- ---------- ---------- ----------
269484_Ecanis@YP_303050                 -E-------- ---------- ---------- ----------
517418_Cthalassium@YP_001997584         ---------- ---------- ---------- ----------
485917_Pheparinus@YP_003091837          ---------- ---------- ---------- ----------
762903_Psaltans@YP_004273747            ---------- ---------- ---------- ----------
929556_Scanadensis@YP_006255074         ---------- ---------- ---------- ----------
760192_Hhydrossis@YP_004446110          ---------- ---------- ---------- ----------
1162668_Lferrooxidans@YP_005469656      ---------- ---------- ---------- ----------
1048260_Lferriphilum@YP_006765680       ---------- ---------- ---------- ----------
240015_Acapsulatum@YP_002755573         -AAQ------ ---------- ---------- ----------
204669_CKoribacter@YP_589562            ---------- ---------- ---------- ----------
1198114_Gtundricola@YP_004219465        -AH------- ---------- ---------- ----------
926566_Troseus@YP_006423699             -SA------- ---------- ---------- ----------
401053_Tsaanensis@YP_004180969          -AVN------ ---------- ---------- ----------
682795_Gmallensis@YP_005055878          -H-------- ---------- ---------- ----------
696127_CMidichloria@YP_004679282        ---------- ---------- ---------- ----------
871271_CZinderia@YP_003878048           ---------- ---------- ---------- ----------
657324_Bfibrisolvens@YP_007819038       -EVESCHMH- ---------- ---------- ----------
768670_Cnitroreducens@YP_004051749      -KIKNRVKS- ---------- ---------- ----------
717231_Fsinusarabici@YP_004603876       -KIKNKETNQ EV-------- ---------- ----------
639282_Ddesulfuricans@YP_003496772      -KIKTEIAS- ---------- ---------- ----------
522772_Dacetiphilus@YP_003503972        -KVKKDN--- ---------- ---------- ----------
693746_Ovalericigenes@YP_004880314      QDCEHCAGE- ---------- ---------- ----------
657321_Rbromii@YP_007781362             -ECDSCGCEG ---------- ---------- ----------
657322_Fprausnitzii@YP_007799942        -EDCEHCESC GN-------- ---------- ----------
213810_Rchampanellensi@YP_007828576     -ECEGCCEGH ---------- ---------- ----------
657319_[siraeum@YP_007775950            -DDHCDSCCG I--------- ---------- ----------
293826_Ametalliredigen@YP_001320267     -NDDHDHHDD IEEE------ ---------- ----------
350688_Aoremlandii@YP_001513205         -DEDHDHHDH EEEE------ ---------- ----------
546269_Falocis@YP_005054244             -EDHEHDHE- ---------- ---------- ----------
1511_[sticklandii@YP_003936518          -DDDHDHHDD IEED------ ---------- ----------
699034_Pdifficile@YP_006198328          -DDAHDHDVE EEE------- ---------- ----------
1128398_[acidurici@YP_006788189         -IDDEDLHAH GHDEE----- ---------- ----------
572544_Ipolytropus@YP_003967871         ---------- ---------- ---------- ----------
469604_Fnucleatum@YP_008477745          ---------- ---------- ---------- ----------
580340_Tlienii@YP_004932350             ---------- ---------- ---------- ----------
891968_Amobile@YP_006444325             -CAKA----- ---------- ---------- ----------
525903_Tacidaminovoran@YP_003316727     -FSHHEDEEG MESRAAD--- ---------- ----------
697281_Maustraliensis@YP_004463371      -EHIHDCADD EAANG----- ---------- ----------
309799_Dthermophilum@YP_002251521       ---------- ---------- ---------- ----------
368407_Mmarisnigri@YP_001047821         -CSGEGGCDS CPGND----- ---------- ----------
903814_Elimosum@YP_003961665            ---------- ---------- ---------- ----------
931626_Awoodii@YP_005268656             ---------- ---------- ---------- ----------
410358_Mlabreanum@YP_001029708          -CGHDCDTCE L--------- ---------- ----------
679926_Mpetrolearius@YP_003895855       -EHHH----- ---------- ---------- ----------
86416_Cpasteurianum@YP_007940944        -AIHEHVHGN ---------- ---------- ----------
929506_Cbotulinum@YP_004396224          -HIHDEVHGK ---------- ---------- ----------
386415_Cnovyi@YP_878355                 -HIHDEVHGK ---------- ---------- ----------
748727_Cljungdahlii@YP_003779382        -TIHEHVHGN ---------- ---------- ----------
1341692_Cautoethanogenu@YP_008700753    -TIHEHVHGN ---------- ---------- ----------
747365_Tnarugense@YP_004437867          -VIKEHQCNN ---------- ---------- ----------
651822_Ffastidiosum@YP_007827223        ---------- ---------- ---------- ----------
572547_Acolombiense@YP_003553035        -LVDEHSHHF HHQG------ ---------- ----------
212717_Ctetani@NP_781697                -HIHDEVHG- ---------- ---------- ----------
431943_Ckluyveri@YP_001394709           -TIHEHVHGQ ---------- ---------- ----------
573061_Ccellulovorans@YP_003843335      -HIHEEVHGH ---------- ---------- ----------
508767_Cbotulinum@YP_001920515          DELHNMVHGE E--------- ---------- ----------
931276_Csaccharoperbut@YP_007454264     EDLHAAVHGE E--------- ---------- ----------
290402_Cbeijerinckii@YP_001308237       EDLHAAVHGE E--------- ---------- ----------
1345695_Csaccharobutyli@YP_008674363    -DLHDMVHGE ---------- ---------- ----------
269797_Mbarkeri@YP_305925               ---------- ---------- ---------- ----------
188937_Macetivorans@NP_617616           ---------- ---------- ---------- ----------
195103_Cperfringens@YP_696469           -DIHEHVHG- ---------- ---------- ----------
593750_Mformicica@YP_007249963          -HEHEDVTCE H--------- ---------- ----------
456442_Mboonei@YP_001405283             -HEHEEGIEC QH-------- ---------- ----------
521011_Mpalustris@YP_002466865          -HEADACDIP PSE------- ---------- ----------
323259_Mhungatei@YP_503638              -HSHDDLACE E--------- ---------- ----------
547558_Mmahii@YP_003541558              ---------- ---------- ---------- ----------
644295_Mevestigatum@YP_003727500        -PE------- ---------- ---------- ----------
351627_Csaccharolyticu@YP_001181022     ---------- ---------- ---------- ----------
521460_Cbescii@YP_002573580             ---------- ---------- ---------- ----------
632292_Chydrothermalis@YP_003992133     ---------- ---------- ---------- ----------
632348_Ckronotskyensis@YP_004023692     ---------- ---------- ---------- ----------
632335_Ckristjanssonii@YP_004026829     ---------- ---------- ---------- ----------
632516_Clactoaceticus@YP_004798736      ---------- ---------- ---------- ----------
608506_Cobsidiansis@YP_003840166        ---------- ---------- ---------- ----------
632518_Cowensensis@YP_004002802         ---------- ---------- ---------- ----------
1121335_[stercorarium@YP_007372524      -CCKCETESE DSEE------ ---------- ----------
1121335_[stercorarium@YP_007678972      -CCKCETESE DSEE------ ---------- ----------
755731_Csp@YP_005148252                 -GECSCCHHS HGREEDFEDE E--------- ----------
394503_Ccellulolyticum@YP_002506259     -GECSCCHHS HGREENFEDE E--------- ----------
720554_[clariflavum@YP_005046370        -GRRCDDLHH HLDEEDDD-- ---------- ----------
```

```
203119_Rthermocellum@YP_001037149        -GRRCDDIHH GIDDED---- ---------- ----------
642492_Clentocellum@YP_004309737         -DVHDHDHHD HDHEEGSGCC ---------- ----------
1384484_Aequolifaciens@YP_008663926      -DISEHDHGR DDEDDE---- ---------- ----------
515619_Erectale@YP_002936246             -DISEKEEAP EEDY------ ---------- ----------
718255_Rintestinalis@YP_007780168        -DISEKEE-- EEEY------ ---------- ----------
657315_Rintestinalis@YP_007830593        -DISEKEE-- EEEY------ ---------- ----------
357809_Lphytofermentan@YP_001560003      -DIHEEEEV- NEEY------ ---------- ----------
610130_[saccharolyticu@YP_003823446      -DIGEEEV-- EEVY------ ---------- ----------
717608_[cf@YP_007849106                  -DIGEEEE-- EEEY------ ---------- ----------
245012_bproducing@YP_007810477           -DIGEEEE-- EEEY------ ---------- ----------
657313_Rtorques@YP_007786815             -DIHEGEEAE EEEY------ ---------- ----------
245018_bproducing@YP_007788847           -DISEGEE-- EEEY------ ---------- ----------
585394_Rhominis@YP_004837975             -DISEGLE-- EEDY------ ---------- ----------
1054217_Tarchaeon@YP_007688600           -DIND----- ---------- ---------- ----------
657323_Rsp@YP_007784833                  -DIHEGEEDT EEEY------ ---------- ----------
657314_Robeum@YP_007804234               -DISEGEE-- EEEY------ ---------- ----------
717962_Ccatus@YP_007770008               -DISEGEE-- EEDY------ ---------- ----------
552811_Dlykanthroporep@YP_003758020      -DEHHH---- ---------- ---------- ----------
246194_Chydrogenoforma@YP_361011         ---------- ---------- ---------- ----------
696281_Druminis@YP_004546667             ---------- ---------- ---------- ----------
868595_Dcarboxydivoran@YP_004497987      ---------- ---------- ---------- ----------
349161_Dreducens@YP_001112128            ---------- ---------- ---------- ----------
634498_Mruminantium@YP_003424309         ---------- ---------- ---------- ----------
406327_Mvannielii@YP_001322697           ---------- ---------- ---------- ----------
679901_Mzhilinae@YP_004616912            ---------- ---------- ---------- ----------
224719_Msp@YP_008075510                  ---------- ---------- ---------- ----------
339860_Mstadtmanae@YP_448034             ---------- ---------- ---------- ----------
1295009_CMethanomassili@YP_008071572     ---------- ---------- ---------- ----------
420247_Msmithii@YP_001272836             ---------- ---------- ---------- ----------
1041930_Mconradii@YP_005380864           -EER------ ---------- ---------- ----------
304371_Mpaludicola@YP_003355464          -EEEHAEDGA SCPPEEHK-- ---------- ----------
1379702_Msp@YP_008915990                 -SSE------ ---------- ---------- ----------
351160_Marvoryzae@YP_686139              ---------- ---------- ---------- ----------
990316_Mconcilii@YP_004384883            -EVQEGDHCD TDEG------ ---------- ----------
521011_Mpalustris@YP_002466708           ---------- ---------- ---------- ----------
1201294_Mbourgensis@YP_006544991         ---------- ---------- ---------- ----------
368407_Mmarisnigri@YP_001048051          ---------- ---------- ---------- ----------
635013_Tpotens@YP_003640662              ---------- ---------- ---------- ----------
457570_Nthermophilus@YP_001916489        -ETQ------ ---------- ---------- ----------
574087_Aarabaticum@YP_003827335          -H-------- ---------- ---------- ----------
748449_Hhalobius@YP_007315822            -DHHHE---- ---------- ---------- ----------
338963_Pcarbinolicus@YP_006717600        ---------- ---------- ---------- ----------
498761_Hmodesticaldum@YP_001680434       ---------- ---------- ---------- ----------
768704_Dmeridiei@YP_006620851            ---------- ---------- ---------- ----------
768706_Dorientis@YP_004969159            ---------- ---------- ---------- ----------
1147129_Dsp@YP_006909410                 ---------- ---------- ---------- ----------
138119_Dhafniense@YP_518658              ---------- ---------- ---------- ----------
645991_Sglycolicus@YP_004266248          ---------- ---------- ---------- ----------
646529_Dacidiphilus@YP_006467680         ---------- ---------- ---------- ----------
272564_Dhafniense@YP_002460033           ---------- ---------- ---------- ----------
756499_Ddehalogenans@YP_006431093        ---------- ---------- ---------- ----------
871963_Ddichloroelimin@YP_007221311      ---------- ---------- ---------- ----------
767817_Dgibsoniae@YP_007945765           ---------- ---------- ---------- ----------
485916_Dacetoxidans@YP_003191710         ---------- ---------- ---------- ----------
555079_Toceani@YP_003825515              ---------- ---------- ---------- ----------
349307_Mthermophila@YP_843171            ---------- ---------- ---------- ----------
760568_Dkuznetsovii@YP_004517387         ---------- ---------- ---------- ----------
373903_Horenii@YP_002508640              -DKATS---- ---------- ---------- ----------
370438_Pthermopropioni@YP_001211605      ---------- ---------- ---------- ----------
264732_Mthermoacetica@YP_430496          ---------- ---------- ---------- ----------
96561_Doleovorans@YP_001528642           -SHDELHRVG DKCYCPYCDS EVSDGEKMCS ACLSDLTEEH
471821_uTermite@YP_001956213             ---------- ---------- ---------- ----------
429009_Adegensii@YP_003238425            -SCAR----- ---------- ---------- ----------
706587_Dtiedjei@YP_006449617             -EVCSCPVCD VNDPEEAPYC SSVCERTTGK ----------
589924_Fplacidus@YP_003435557            -KLSEEEHEE EVCEV----- ---------- ----------
224325_Afulgidus@NP_069024               -KELEKMEKG EMDDHGEYCE A--------- ----------
224325_Afulgidus@NP_069399               -KELEKMEKG EMDDHGEYCE A--------- ----------
416591_Tlettingae@YP_001470347           ---------- ---------- ---------- ----------
688269_Tthermarum@YP_004660083           ---------- ---------- ---------- ----------
374847_CKorarchaeum@YP_001737319         -SVERQALEE EEMIERIYGK LRR------- ----------
583356_Iaggregans@YP_003860035           -QEEKLVLEE EEMIERYHKS MKEVMGVVDR K---------
272947_Rprowazekii@NP_220862             ---------- ---------- ---------- ----------
1282356_Ppoae@YP_007398800               ---------- ---------- ---------- ----------
994484_Pbrassicacearum@YP_004352099      ---------- ---------- ---------- ----------
882944_Languillarum@YP_008488748         ---------- ---------- ---------- ----------
1167634_Axylosoxidans@YP_008032542       ---------- ---------- ---------- ----------
257311_Bparapertussis@NP_884287          ---------- ---------- ---------- ----------
1017264_Bpertussis@YP_005589900          ---------- ---------- ---------- ----------
284812_Spombe@NP_592967                  -SATA----- ---------- ---------- ----------
559292_Scerevisiae@NP_014869             ---------- ---------- ---------- ----------
559292_Scerevisiae@NP_015190             ---------- ---------- ---------- ----------
9606_Hsapiens@NP_998760                  ---------- ---------- ---------- ----------
9606_Hsapiens@NP_055116                  ---------- ---------- ---------- ----------
3702_Athaliana@NP_193953                 TQA------- ---------- ---------- ----------
3702_Athaliana@NP_192317                 STYLHGIGS- ---------- ---------- ----------
3702_Athaliana@NP_186751                 KA-------- ---------- ---------- ----------
7227_Dmelanogaster@NP_649840             ---------- ---------- ---------- ----------
6239_Celegans@NP_502658                  ---------- ---------- ---------- ----------
```
