# Supplementary material for: Turning Escherichia coli into a Frataxin-Dependent Organism
Source: PLoS Genet. 2015 May 21;11(5):e1005134. doi: 10.1371/journal.pgen.1005134 (PMC4440780; doi:10.1371/journal.pgen.1005134)
Supplement: S1 Text — (DOCX) [file pgen.1005134.s009.docx]

**Supporting Information**

**Reference**

86. Guzman LM, Belin D, Carson MJ, Beckwith J. Tight regulation, modulation, and high-level expression by vectors containing the arabinose PBAD promoter. J Bacteriol. 1995;177: 4121-4130.

**Materials and methods**

Growth conditions

Strains were grown at 37°C in minimal medium (M9) supplemented with glucose (0.4%) and MgSO_4_ (1 mM). Amino acids (0.5 mM), thiamine (0.2 µg/mL) and nicotinic acid (12.5 µg/mL) were added as required.

Probe of the oligomeric state of IscU proteins in solution

Analytical gel filtration experiments were performed using a Superdex 75 10/300 GL column (GE Healthcare). The column was equilibrated with buffer A (0.1 M Tris-HCl, pH 8, 50 mM NaCl). Ovalbumin (43 kDa), chymotrypsinogen A (25 kDa) and ribonuclease A (13.7 kDa) were used as molecular standards for the mass calibration. Purified IscU_WT_ and IscU_IM_ (3 mg in 500 µL buffer A) were loaded using a static loop (1 mL) and were eluted with buffer A.

Elution profile of CyaY, IscS, IscU_WT_ and IscU_IM_ proteins on QFF column

1 mg of purified protein CyaY, IscS, IscU_WT_ or IscU_IM_ was loaded onto a 1-mL anion exchange column (QFF) (GE Healthcare) equilibrated with buffer A (50 mM Tris-HCl, pH 8). Samples were eluted with a gradient of buffer A containing 1 M NaCl.

Kinetics of Fe-S formation

To assess kinetics of cluster formation on IscU_WT_ or IscU_IM_, absorbance at 420 nm were measured as a function of time. 25 µM IscU_WT_ or IscU_IM_ was incubated anaerobically with 100 µM Fe(SO_4_)_2_(NH_4_)_2_, 2 mM DTT, with or without 25 µM CyaY, in 50 mM Tris-HCl pH 8. Subsequently, 25 µM IscS and 250 µM L-cysteine were added to start the reaction.
