# Supplementary material for: Turning Escherichia coli into a Frataxin-Dependent Organism
Source: PLoS Genet. 2015 May 21;11(5):e1005134. doi: 10.1371/journal.pgen.1005134 (PMC4440780; doi:10.1371/journal.pgen.1005134)
Supplement: S2 Table — (DOCX) [file pgen.1005134.s011.docx]

**S2 Table.** Oligonucleotides used in this study.

| **Primer name** | **Sequence (5’-3’)^a^** |
| --- | --- |
| Construction of the *iscU_IM_* allele |  |
| IscU-UPBamH1 | CCGGGGATCCCTTTAGGTCGTTTTACTACTGAAGA |
| IscU_I108M_-DO | TTCTGCCAGCATAGAACAGTGAATTTTCACCGGCG |
| IscU_I108M_-UP | CTGTTCTATGCTGGCAGAAGACGCGATCAAAGCCG |
| IscU-DOXbaI | CCGGTCTAGATAACATTCTCAGGGCTGCGATCCGC |
| Cloning of *iscU* and *iscU_IM_* in pBAD24 vector |  |
| NcoI-IscU | CCGGCCATGGCTTACAGCGAAAAAGTTATCGAC |
| HindIII-IscU | CCGGAAGCTTTTATTTTGCTTCACGTTTGCTTTT |
| IscU_I108M__for | CGGTGAAAATTCACTGTTCTATGCTGGCAGAAGACGCGATCAAAGCCGC |
| IscU_I108M__rev | TTTGATCGCGTCTTCTGCCAGCATAGAACAGTG AATTTTCACCGGCGGC |
| Cloning of *iscU* and *iscU_IM_* in pET21a+ vector |  |
| NdeI-IscU | CGGCCATATGGCTTACAGCGAAAAAGTTATCG |
| HindIII-IscU | GGCGAAGCTTTTTTGCTTCACGTTTGCTTTTATAG |

^a^ Underlining indicates the recognition sites of restriction enzymes.
