# Supplementary material for: Turning Escherichia coli into a Frataxin-Dependent Organism
Source: PLoS Genet. 2015 May 21;11(5):e1005134. doi: 10.1371/journal.pgen.1005134 (PMC4440780; doi:10.1371/journal.pgen.1005134)
Supplement: S3 Table — (DOCX) [file pgen.1005134.s012.docx]

**S3 Table.** Bacterial strain and plasmids used in this study.

| **Strain or plasmid** | **Relevant genotype** | **Reference or source** |
| --- | --- | --- |
| *E. coli* K-12 strains |  |  |
| BR668 | *lacIpoZ ∆*(*Mlu*) λ -P*_iscR_-lacZ* *∆iscU::kan ∆cyaY::spec* | This study |
| Plasmids |  |  |
| pBAD24 | Cloning vector, Amp^R^ | [86] |
| pIscU | pBAD24 expressing IscU | This study |
| pIscU_IM_ | pBAD24 expressing IscU_I108M_ | This study |
